# Bromine radical release from a nickel-complex facilitates the activation of alkyl boronic acids: a boron selective Suzuki–Miyaura cross coupling†

**DOI:** 10.1039/d4sc04196h

**Published:** 2024-09-26

**Authors:** Monica Oliva, Serena Pillitteri, Johannes Schörgenhumer, Riku Saito, Erik V. Van der Eycken, Upendra K. Sharma

**Affiliations:** a Laboratory for Organic & Microwave-Assisted Chemistry (LOMAC), Department of Chemistry, University of Leuven (KU Leuven) Celestijnenlaan 200F B-3001 Leuven Belgium; b Department of Chemistry, University of Zurich Winterthurerstrasse 190 CH-8057 Zurich Switzerland; c Department of Chemistry and Centre for Sustainable Chemistry, Ghent University Krijgslaan 281 (S3) 9000 Ghent Belgium; d Peoples' Friendship University of Russia (RUDN University) Miklukho-Maklaya Street 6 117198 Moscow Russia; e Department of Chemistry and Biochemistry, University of Missouri-St. Louis, One University Boulevard St. Louis MO 63121 USA sharmauk@umsl.edu

## Abstract

In this study, without utilizing any exogenous activator or strong oxidants, we successfully employed inactivated and easily accessible alkyl boronic acids (BAs) as coupling partners in a photocatalyzed Suzuki–Miyaura reaction under batch and continuous-flow conditions. Detailed mechanistic studies suggest a unique BA activation pathway, *via* a plausible radical transfer event between a bromine radical (formed *in situ via* a photo-induced homolysis of the Ni–Br bond) and the empty p-orbital on the boron atom. Subsequently, the necessity to tune the BA oxidation potential by means of hydrogen-bonding interaction with solvents or Lewis acid–base type interactions is replaced by a novel halogen radical transfer (XRT) mechanism. The mechanistic hypothesis has been supported by both control experiments and DFT calculations.

## Abstract 2

The release of bromine radicals from a nickel complex *via* a homolytic cleavage under visible light allows the activation of alkyl boronic acids, which participate in a Suzuki–Miyaura cross-coupling process under batch and continuous flow settings.

## Graphical abstract

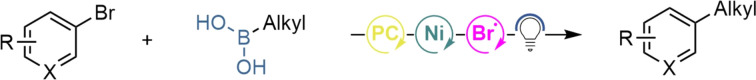

Over the past decades, developments in nickel-catalysed cross-coupling reactions have led to the discovery of an entirely new chemical space, with significant implications for the formation of novel C(sp^2^)–C(sp^3^) bonds.^1,2^ This is due to the unique reactivity of nickel, which is attributed to a more stable open shell configuration than its second and third row counterparts, allowing the metal to undergo not just two-electron pathways, but also single-electron transfer processes.^3–5^

With the advent of photoredox catalysis, the merging of nickel chemistry with single-electron transfer (SET) events came as no surprise, revolutionizing the way bond disconnections are designed.^6,7^ A new set of coupling partners has been unlocked to forge C(sp^2^)–C(sp^3^) bonds, without the need of strong stoichiometric oxidants and avoiding high-energy light sources, thus marking a significant milestone in the field of cross-coupling chemistry. Several examples in the literature unveiled the remarkable properties associated with this metal, including its capability to be excited by visible light, which opens up a whole new range of reactivity. Notably, as reported by Molander's group,^8^ nickel complexes can undergo an energy transfer mechanism with a photocatalyst or can be excited by visible light and extrude a halogen radical (Fig. 1a)^8–10^ or an aryl radical,^11^ can be involved in C–O reductive elimination^12^ and amine to Ni-electron transfer^13^ or can be employed with the double role of both the metal and photocatalyst.^14–16^ Interestingly, homolysis of the Ni–Br bond with consequent extrusion of one bromine radical has been frequently exploited in photocatalyzed reactions:^8–10,17^ thanks to the *in situ* formed bromine radical's capability to abstract hydrogen atoms from alkyl feedstocks *via* a HAT mechanism.

**Fig. 1:**
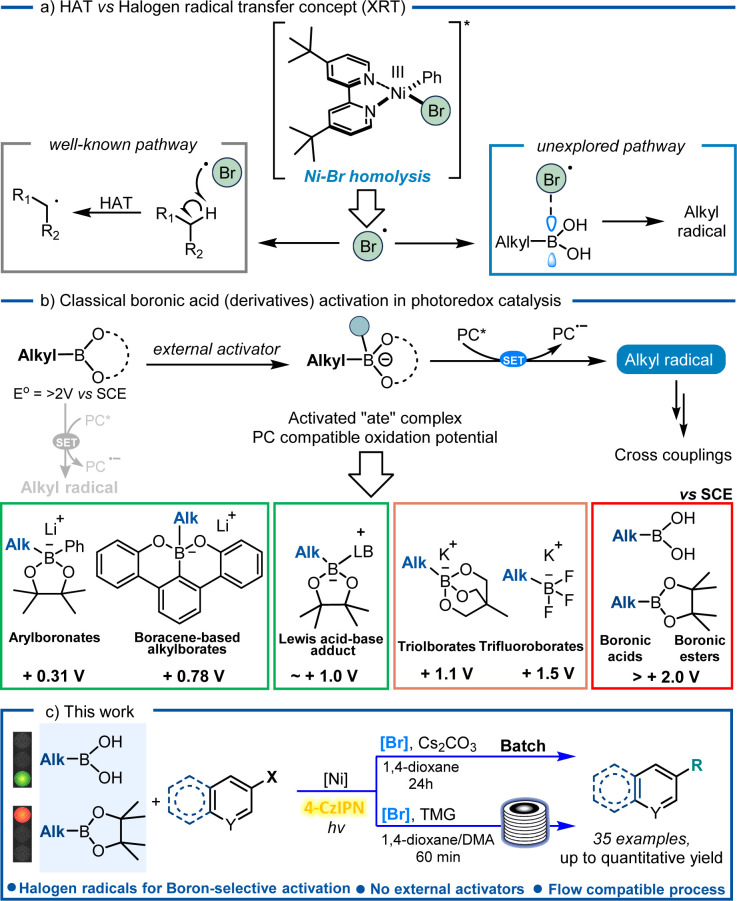
(a) Background on Suzuki–Miyaura cross-coupling; (b) excited Ni-complexes extrude a Br radical which can accomplish beyond HAT; (c) progress in boronic acid derivative activation in photoredox catalysis.

Boronic acids are one of the most ubiquitous classes of reagents in modern organic synthesis, including their alkyl counterparts, which find extensive applications in medicinal chemistry (*e.g.* ixazomib, tavaborole, and crisaborole),^18^ chemosensing,^19^ material chemistry,^20^ and biomedical engineering,^21^ which contributes to their commercial availability. Despite the use of boronic acids as coupling partners in traditional Suzuki–Miyaura couplings, the application of their alkyl ester counterpart has been widely explored in the last few decades for photocatalyzed synthetic strategies. Interestingly, a quinoline–boronic ester complex has been shown to react effectively for radical generation in a Ni/photoredox-catalyzed C(sp^2^)–C(sp^3^) cross-coupling reaction.^22^ Moreover, also alkyl boranes have recently shown to be suitable precursors for metallophotoredox reactions, thus expanding the coupling partners' portfolio at disposition for the incorporation of sp^3^ hybridized carbons. Nonetheless, alkyl boronic acids still face a limited application in this field.^23^ Following our desire to use alkyl boronic acids as valid alternatives to their ester and negatively charged tetrahedral borate salts as radical precursors (Fig. 1b), we employed them in a Suzuki–Miyaura reaction under visible-light conditions. Despite our previous finding on BA activation *via* means of Lewis base additives, solvents or the substrate itself,^24^ herein we observed a reactivity which was governed by halogen radicals (Br˙) extruded from the Ni-complex, hereby proposing an alternative mechanistic pathway towards their activation. The reaction was selective towards free boronic acids as compared to boronic esters and even HAT prone solvent (1,4-dioxane), and the results are consistently supported by DFT calculations (Fig. 1c).

Based on our previous experience on the activation of alkyl boronic acids *via* amide-based solvents towards redox active species, we started the investigation for the coupling reaction with cyclopentyl boronic acid (1a) and 4-bromobenzoate (2a) using NiCl_2_·glyme, dtbbpy, (Ir[dF(CF_3_)ppy]_2_(dtbpy))PF_6_, Cs_2_CO_3_ and dimethylacetamide (DMA) as solvent (Table S1, ESI†). We reasoned that, following visible-light excitation, the photocatalyst in its triplet state oxidises the DMA–boronic acid complex.^24^ The resultant radical can then be trapped by the Ni(ii) complex generated by the oxidative addition of Ni(0) on 4-bromobenzoate, resulting in a Ni(iii) species containing both the alkyl and aromatic components. After reductive elimination, the desired compound (3a) is formed, while Ni(i) and the reduced photocatalyst undergo SET to regenerate the respective Ni-catalyst and resume the cycle. We were pleased to obtain the desired product 3a in 41% yield (entry 9, Table S1, ESI†). Surprisingly, solvent screening investigations (detailed optimization studies in the ESI†) found that the desired coupled compound could be obtained not only with amide-based solvents, but also with solvents which are not known to form Lewis acid–base adducts with the vacant p-orbital of the boron atom (see Table S1† in the ESI).^24^ This encouraged us to continue our research with the prospect of a novel activation mode towards radical generation in mind. In addition, we were pleased to observe that the reaction performs well with the 4CzIPN, thus avoiding the necessity to use expensive iridium-based photocatalysts to perform the coupling under batch conditions. Having excluded an underlying thermal mechanism and the indispensability of light and Cs_2_CO_3_ (Table 1, entries 4 and 5), we were able to synthesize 3a with 78% yield (Table 1, entry 3). NiBr_2_·dtbbpy gave the best yield compared to other Ni catalysts (Table 1, entry 2 and ESI†), and this led us to suspect the involvement of Br anions or radicals in the reaction mixture. To test our hypothesis, we added a catalytic amount of tetrabutylammonium bromide (TBABr) as a Br source to the standard conditions resulting in an increased yield (90%) of 3a (Table 1, entry 1).

**Table 1:** Optimization of the reaction conditions^a^

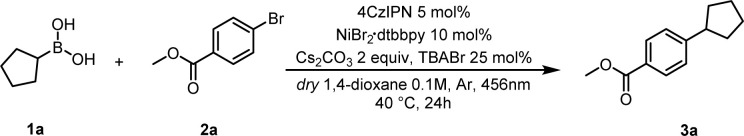		
Entry	Deviation from standard conditions	Yield
1	None	90%
2	With NiCl_2_·dtbbpy without TBABr	41%
3	Without TBABr	78%
4	Without light	N.R.
5	Without base	N.R.
6	Without light and photocatalyst, at 40 °C	N.R.

a Reaction conditions: 1a (0.15 mmol), 2a (0.1 mmol), 4CzIPN (0.005 mmol), NiBr_2_dtbbpy (0.01 mmol), Cs_2_CO_3_ (0.2 mmol), TBABr (0.025 mmol), 1,4-dioxane (1 mL), Ar, 40 °C, irradiated by blue light (456 nm). Reactor used: PhotoRedOx Box Duo; EvoluChem™©.

## Flow chemistry optimization

Merging photoredox catalysis with continuous-flow technology has proven to be an invaluable tool for the fast and effective production of molecular structures for both academic and industrial applications.^25^ To begin with, we conducted a batch screening to determine which soluble organic base could be an effective substitute for Cs_2_CO_3_.

Fortunately, 1,1,3,3-tetramethylguanidine (TMG) showed promising results when combined with the Ir-photocatalyst and NiBr_2_·dtbbpy, delivering the desired product in 68% yield (Table S8, ESI†). In contrast to batch optimization (Table 1), the addition of TBABr resulted in a lower yield (43%). This anomaly could be attributed to the plausible coordination between the nickel catalyst and the employed base, thus affecting its reactivity under modified conditions.^26,27^ With these results in hands, a careful solvent optimization was performed, as the formation of TMG·HBr salt resulted in flow channel clogging. Eventually three factors were crucial to obtain optimal yields: (a) a 1 : 4 mixture of DMA and 1,4-dioxane, (b) 40 °C temperature obtained by simply turning off the fan, and (c) 3 equivalents of TMG resulting in 84% yield (Table 2, entry 5).

**Table 2:** Optimization of the reaction conditions in continuous flow^a^

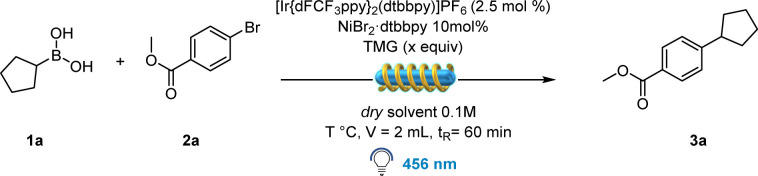				
Entry	TMG (equiv.)	Solvent	*T* (°C)	Yield
1	2	1,4-Dioxane	30	Clogged
2	2	DMA	30	49%
3	2	DMA/1,4-dioxane	30	69%
4	2	DMA/1,4-dioxane	40	76%
5	3	DMA/1,4-dioxane	40	84%

a Selected entries. Reaction conditions: 1a (0.15 mmol), 2a (0.1 mmol), PC (0.0025 mmol), NiBr_2_·dtbbpy (0.01 mmol), TMG, solvent (1 mL in 1 : 4 ratio), irradiated by 456 nm light, isolated yield. Reactor used: PhotoRedOx Box Duo; EvoluChem™© with flow insert.

## Scope and limitations

Having established the optimal conditions for this protocol, we focused our attention on the scope of the alkyl boronic acids. Notably, the procedure is amenable for the synthesis of C(sp^2^)–C(sp^3^) bonds generating both secondary and primary alkyl radicals (Table 3).

**Table 3:** Scope of alkyl boronic acids and different electrophiles in batch and continuous flow: reaction conditions in the case of haloarene and triflate scope: 1 (0.3 mmol), 2 (0.2 mmol), 4CzIPN (0.01 mmol), NiBr_2_·dtbbpy (0.02 mmol), Cs_2_CO_3_ (0.4 mmol), TBABr (0.05 mmol), 1,4-dioxane (2 mL), Ar, 40 °C, irradiated by 456 nm light. In the case of acyl chloride scope: 1 (0.45 mmol), 2 (0.3 mmol), [Ir(dFCF_3_ppy)_2_(bpy)]PF_6_ (0.006 mmol), NiCl_2_·glyme (0.012 mmol) dtbbpy (0.012 mmol), Cs_2_CO_3_ (0.6 mmol), THF (3 mL), Ar, 40 °C, irradiated by 456 nm light. Reaction conditions in continuous flow: 1a (1.5 mmol), 2a (1 mmol), [Ir(dFCF_3_ppy)_2_(dtbbpy)]PF_6_ (0.025 mmol), NiBr_2_·dtbbpy (0.1 mmol), TMG (3 mmol), 1,4-dioxane (8 mL), DMA (2 mL), 40 °C, irradiated by 456 nm light. Reactor used: Vapourtec easy-Photochem E-series

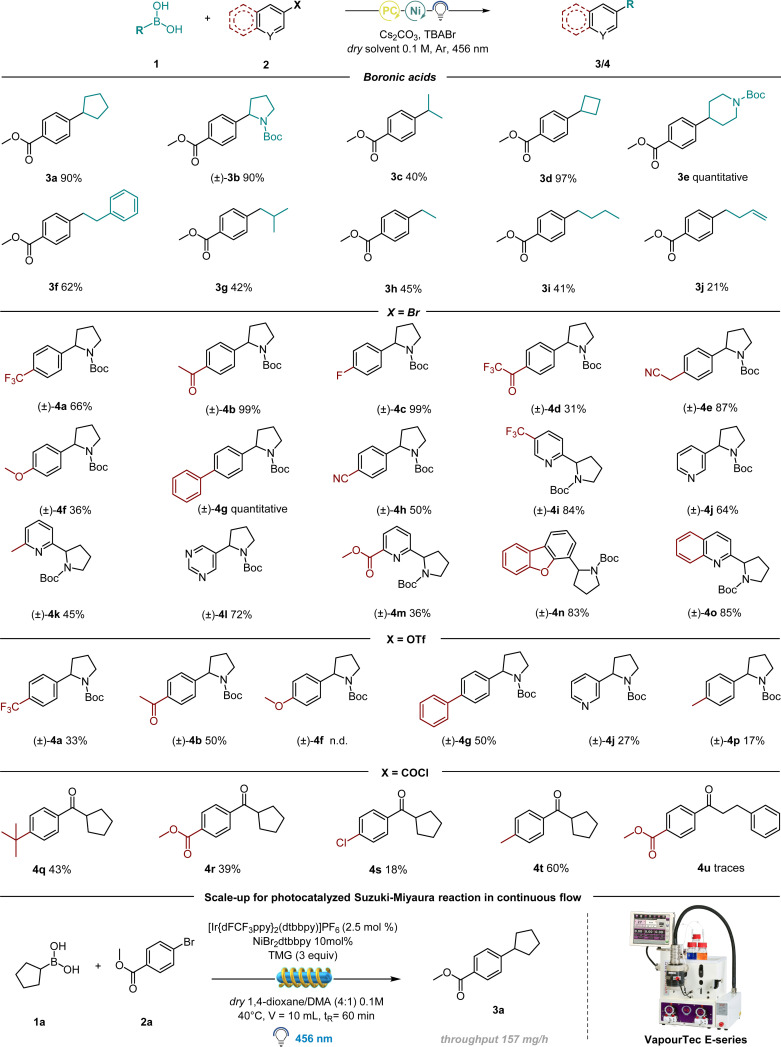

Highly successful reaction partners 1-*N*-Boc-pyrrolidin-2-ylboronic acid and 1-*N*-Boc-piperidine-4-boronic acid provided overall the best yield, delivering 3b and 3e respectively in 90% and quantitative yield. Smaller strained rings as cyclobutyl boronic acid also delivered the desired coupling product in excellent yield (97%, 3d). Acyclic primary derivatives could be successfully synthesized as well, although in moderate yields (41–45%, molecules 3g, 3h and 3i), while the synthesis of molecule 3f resulted in 62% yield, probably thanks to the presence of a benzyl group on the fragment responsible for radical stabilization and thus higher yield. We then turned our attention towards the scope of various electrophilic coupling partners (Table 3) with 1-*N*-Boc-pyrrolidin-2-yl boronic acid as a preferred radical precursor. Electron poor rings performed well under the given conditions delivering the desired product in good to excellent yields. Different electron withdrawing groups have been tested: fluorine-containing molecules have been successfully synthesized *e.g.*4a (66% yield), 4c (99% yield) and 4d (31% yield). Nitrile-containing scaffolds effectively delivered the desired product (4e, 87% and 4h, 50%). Next, different molecular structures containing biologically relevant heterocyclic scaffolds underwent the developed protocol, delivering satisfying yields: dibenzofuran 4n (83% yield), quinoline 4o (85% yield) and pyrimidine 4l (72%). The reaction was further attempted with *p*-OMe bromobenzene, delivering a moderate yield (4f). The reaction was therefore further performed in the presence of electron rich aryl iodides, proving to be more effective (see ESI† for further information). Finally, different pyridine-containing molecules have been successfully synthesized, with both electron-withdrawing and electron-donating groups on the ring (4i–4k and 4m, 36–84%).

Having established the feasibility of the developed protocol using bromo arenes as the electrophilic partner, we turned our attention to test different electrophiles. As shown in Table 3, molecules 4a, 4b, 4g, and 4j could be successfully synthesized starting from their corresponding triflates as the starting material, albeit in moderate yields. This could be possibly due to a less efficient oxidative addition of the employed Ni catalyst onto the C-OTf bond compared to the C–Br bond. Remarkably, one example with an electron-rich ring as in molecule 4p could be synthesized using -OTf as an electrophilic partner, while the corresponding bromo benzene failed. Similarly, benzoyl chlorides were tested as well, although a deviation from the standard condition was necessary: by employing THF as a solvent. Further, the use of catalytic additive TBABr was forbidden, as the released bromine radicals underwent the HAT mechanism with the solvent, rather than activating BAs towards radical formation. To totally suppress the formation of the HAT side product, NiCl_2_·glyme was used as the catalyst. We were pleased to observe the formation of the desired products 4q, 4r, 4s, and 4t, even in the absence of Br sources. These results showed the presumable ability of Cl radicals to activate BAs, although with lower efficiency compared to Br (see ESI†).

In order to perform this C(sp^2^)–C(sp^3^) coupling at a higher scale in continuous-flow, we employed a Vapourtec E-series flow reactor with a reaction volume of 10 mL. We carried out our investigation on a 1 mmol scale and gladly the optimized conditions performed well in the automated Vapourtec system, converting 100% of the starting material and delivering the desired compound 3a with a throughput of 157 mg h^−1^ (Table 3).

## Mechanistic studies

**Fig. 2:**
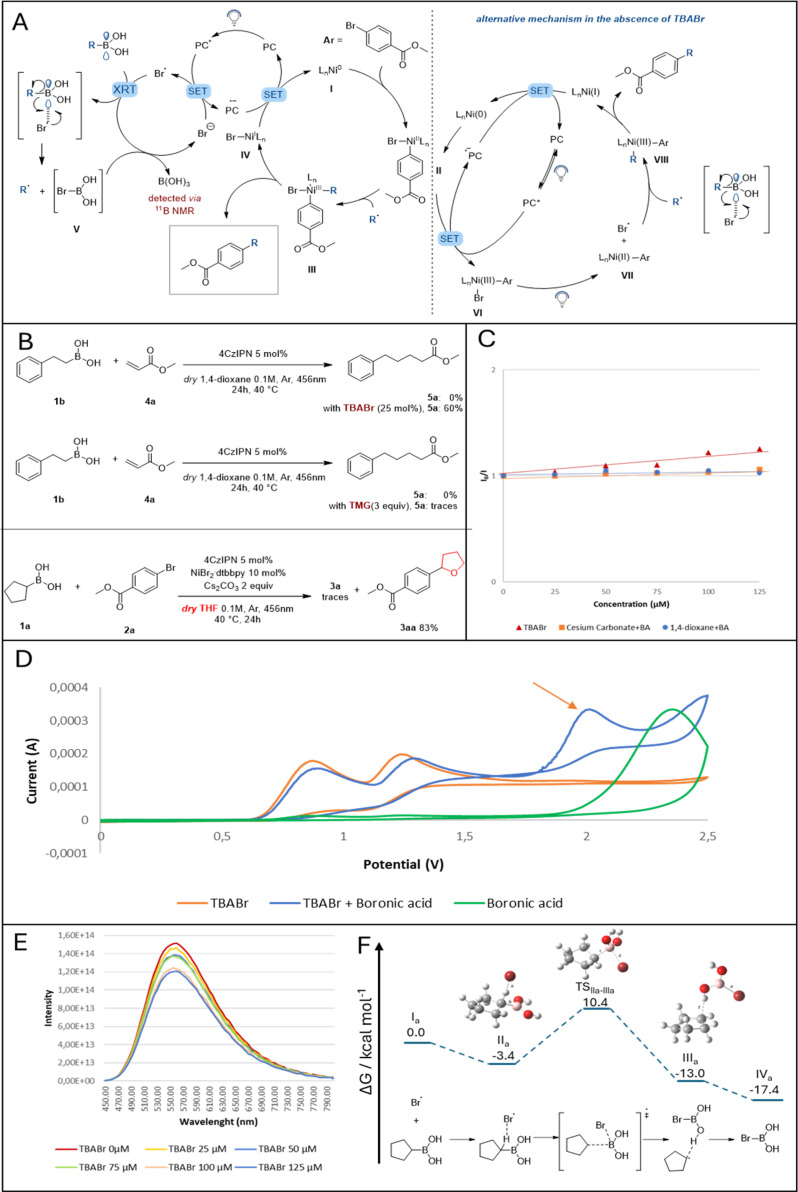
A) Proposed mechanisms in the presence of TBABr (on the left) and in the absence of TBABr (on the right). (B) Radical trapping experiment: Br radicals engage in HAT with the employed solvent. (C) Stern–Volmer plotting different reagents to evaluate BA activation. (D) Cyclic voltammetry experiments and (E) fluorescence quenching. (F) Free energy diagram for the activation pathway of the cyclopentylboronic acid by a bromine radical. Calculations were performed on the IEFPCM (1,4-dioxane)-B3LYP-GD3BJ/6-311+G(d,p) level of theory, energies are relative to the uncoordinated starting compounds (I). For further computational details, please refer to the ESI.†

Looking at the partners engaged in the developed photo-catalyzed Suzuki–Miyaura batch reaction, 1,4-dioxane and/or Cs_2_CO_3_ can be assigned as plausible activation reagents for the selected alkyl boronic acid *via* a Lewis acid–base type interaction. Different mechanistic experiments were carried out to investigate their role in the reaction, as shown in Fig. 2C and ESI.† While inorganic bases such as NaOH,^28^ NaOMe^29^ and K_3_PO_4_ (ref. 30) have been demonstrated to interact with the empty p-orbital on the boron atom of benzyl and alkyl boronic acids (forming a light–sensitive complex), Cs_2_CO_3_ has not yet demonstrated similar reactivity.^31^ Therefore, in order to evaluate its role in the reaction, first, ^11^B NMR measurements were done on a mixture between phenethylboronic acid and Cs_2_CO_3_ in various concentrations, revealing the formation of the boronate complex (Fig. S3, ESI†). This complex was then evaluated for photoactivity *via* fluorescence quenching and cyclovoltammetry experiments. Both experiments demonstrated the complex's inactivity towards energy transfer or single-electron transfer events with the photocatalyst due to inaccessible redox potential (Fig. S8, ESI†).

Next, the ability of 1,4-dioxane to complex cyclopentyl boronic acid was investigated. Despite the fact that ^1^H NMR spectra revealed the formation of a weak hydrogen bond between the solvent and the boronic acid's hydrogens (Fig. S4, ESI†), thus supporting a hypothetical Lewis acid–base interaction between 1,4-dioxane and BA, a solution of these two reactants did not show any photocatalyst quenching (Fig. S9, ESI†). Moreover, cyclovoltammetry experiments on the same solution did not show the formation of any redox-active complex. After ruling out the potential of both solvent and base to form any transient photoactive “ate” complex, we shifted our attention towards the nickel catalyst and the likelihood of releasing bromine radicals in the reaction mixture. As previously indicated (Fig. 1a), Ni(iii) complexes are prone to extrude halogen radicals after visible-light irradiation which are consequently engaged in the HAT mechanism with inert alkyl feedstocks.^32^ A simple switch in our solvent system, from 1,4-dioxane to tetrahydrofuran, confirmed the formation of bromine radicals by the isolation of molecule 3aa (Fig. 2B).^8^ To our surprise, 1,4-dioxane did not show any HAT activation in the presence of BAs.

With this preliminary information in hand, we proposed a plausible reaction mechanism as shown in Fig. 2A (on the right): after oxidative addition of Ni(0) into the bromo arene, the resulting Ni(ii) complex II (*E*_1/2_[Ni^II^/Ni^III^] = +0.65 V *vs.* SCE in CH_3_CN, Fig. S11, ESI†) undergoes SET with the excited photocatalyst (*E*_1/2_[*Ir^III^/Ir^II^] = +1.21 V *vs.* SCE in CH_3_CN). The oxidized Ni(iii) complex VI can be excited by blue-light and extrudes one bromine radical.^33^ The latter interacts with the empty p-orbital on the boron atom, releasing the desired alkyl radical which then can be trapped by Ni(ii) VII. The consequent Ni(iii) complex VIII, after reductive elimination, forms the desired compound and Ni(i). The cycle is closing by regenerating both the photocatalyst and the metal catalyst after SET between the Ni(i) complex and the reduced photocatalyst.

To elucidate the role of Br anions in the reaction mixture (Table 1, entry 1), TBABr was employed as an external additive. A simple control experiment of radical trapping was carried out: TBABr and 4CzIPN were introduced in a mixture of phenethylboronic acid 1b and methyl acrylate 4a in 1,4-dioxane. As shown in Fig. 2B, when no additive is present, the desired product formation was not observed. In contrast, 60% of the expected product 5a were formed in the presence of TBABr. This strongly suggests the ability of TBABr to form alkyl radicals from BAs. To further investigate the reactivity of TBABr under photocatalytic conditions, both cyclovoltammetry (CV) experiments and Stern–Volmer quenching studies were performed. As shown in Fig. 2D, CV analysis of a mixture between TBABr and cyclopentylboronic acid results in the formation of a new peak in the curve, yet its redox potential is too high to engage in SET with the photocatalyst. On one hand, this result could suggest the exclusion of the formation of a photoactive “ate” complex resulting from the interaction between boronic acid and the added bromine anions and support the hypothesis of bromine radicals involved in BA activation. Quenching experiments, on the other hand, confirmed the ability of TBABr to quench the excited state photocatalyst and thus release bromine radicals in the reaction mixture (Fig. 2F and B).^17^

With this information, we proposed a complementary mechanism active in the presence of TBABr (Fig. 2A, on the left): the excited state of the photocatalyst is quenched by the bromine anions derived from TBABr to form bromine radicals. The latter interacts with the empty p-orbital on the boronic acid (*via* a plausible radical transfer strategy)^34^ to release an alkyl radical, which can then be trapped by Ni(ii) species II derived by the oxidative addition of a bromoarene. Ultimately, the formed Ni(iii) complex III undergoes reductive elimination delivering the desired C(sp^2^)–C(sp^3^) coupling molecule, while the cycle is closed by SET between the reduced photocatalyst and Ni(i) species IV. We postulated the formation of compound V as a consequence of the interaction between a bromine radical and BA. Due to the anticipated high instability of this complex, bromine anions should be liberated from it to enter the photocatalyst cycle, eventually generating boric acid, which was detected by ^11^B NMR. This assumption is further supported by the fact that only a catalytic amount of TBABr is required for the reaction.

While the Ni-catalytic cycle for the reaction is mechanistically well established, we set out to investigate the activation mechanism of the boronic acid by DFT calculations using cyclopentylboronic acid and a bromine radical as starting points. Other activation approaches *via* ionic pathways and various (pre)complexation possibilities were investigated but did not deliver any viable result (See ESI† for more information). As shown in Fig. 2F, the approach of the bromine radical was calculated to proceed *via* an interesting intermediate IIa with stabilizing coordination (Δ*G* = −3.4 kcal mol^−1^) to the alkyl scaffold by a hydrogen-bonding interaction *ipso* to the boronic acid moiety (distance 1.8701 Å). The found transition geometry TS_IIa–IIIa_ for the subsequent substitution shows the C–B bond already significantly elongated (1.5785 Å *vs.* 1.7713 Å). The corresponding free energy of 10.4 kcal mol^−1^ relative to the starting compounds (I) is adequate for the applied experimental conditions. The substitution results in bromoboronic acid coordinated to the secondary radical in an intermediary complex IIIa (distance 2.0191 Å). After removal of the alkyl radical either directly by subsequent reaction with the Ni catalyst or by intermediary coordinative replacement by a Lewis base such as the solvent (more details can be found in the ESI†), the clearly exothermic pathway ends in the bromoboronic acid IVa (Δ*G* = −17.4 kcal mol^−1^). Based on our additional computational efforts, we hypothesize that acid IVa is then converted in the presence of residual water in a single step, leading to the energetically stable boric acid, whose formation was confirmed by ^11^B-NMR spectroscopy (for details see ESI†). The byproduct, hydrogen bromide, would be immediately quenched by the basic reaction medium and hence liberate a bromide anion to re-enter the photocatalytic cycle.

In conclusion, in this study, a novel photocatalyzed Suzuki–Miyaura reaction was carried out using both primary and secondary alkyl boronic acids as radical precursors for the construction of C(sp^2^)–C(sp^3^) bonds under mild reaction conditions both in batch and continuous flow. Surprisingly, a unique activation strategy for BAs has been discovered, *via* a plausible radical transfer event between a bromine radical (formed *in situ via* a photo-induced homolysis of the Ni–Br bond) and the empty p-orbital on the boron atom. With this approach, we have shown a convenient method which can overcome the necessity to match the redox potential of BAs used as radical precursors and the employed photocatalyst. Consequently, the necessity to tune the BA oxidation potential by means of hydrogen bonding interaction with solvents or Lewis acid–base type interactions is replaced by a novel halogen radical transfer (XRT) mechanism. The mechanistic hypothesis has been supported by both control experiments and DFT calculations.

## Data availability

All experimental and computational data can be found in the manuscript and in the ESI.† Raw data are available from the authors upon request.

## Author contributions

The authors confirm contributions to the paper as follows: study conception and design: M. O., U. K. S.; methodology and experiments: M. O., S. P. R. S.; DFT calculations: J. S.; manuscript preparation: M. O., S. P., J. S., U. K. S., E. V. d. E.

## Conflicts of interest

There are no conflicts to declare.

## Supplementary Material

SC-OLF-D4SC04196H-s001
