# Supplementary material for: Bromine radical release from a nickel-complex facilitates the activation of alkyl boronic acids: a boron selective Suzuki–Miyaura cross coupling
Source: Chem Sci. 2024 Sep 26;15(42):17490–7. doi: 10.1039/d4sc04196h (PMC11450759; doi:10.1039/d4sc04196h)
Supplement: SC-015-D4SC04196H-s001 [file SC-015-D4SC04196H-s001.pdf]

Supplementary Information (SI) for Chemical Science.
This journal is © The Royal Society of Chemistry 2024

Supporting information

Bromine Radical Release from Nickel-Complex Facilitates the Activation of Alkyl Boronic Acids: A Boron Selective Suzuki-Miyaura Cross Coupling

*Monica Oliva,[a] Serena Pillitteri,[a] Johannes Schörgenhumer,[b] Riku Saito,[c] Erik V. Van der Eycken [a,d] and Upendra K. Sharma [a,e,]**

[a] Laboratory for Organic & Microwave-assisted Chemistry (LOMAC)
Department of Chemistry, KU Leuven Celestijnenlaan 200F, 3001 Leuven, Belgium

[b] Department of Chemistry, University of Zurich
Winterthurerstrasse 190, CH-8057 Zurich, Switzerland

[c] Department of Chemistry and Centre for Sustainable Chemistry, Ghent University, Krijgslaan 281 (S3), 9000 Ghent, Belgium

[d] People's Friendship University of Russia (RUDN University),
Miklukho-Maklaya street 6, RU-117198 Moscow, Russia.

[e] Department of Chemistry and Biochemistry, University of Missouri-St. Louis, One University Boulevard, St. Louis, MO 63121, USA. E-mail: sharmauk@umsl.edu

Contents

1. General information 2
2. Reaction optimization 3
3. $NiBr_2$·dtbbpy synthesis and characterization 6
4. Mechanistic investigations 7
   4.1 NMR Experiments 7
   4.2 Stern-Volmer Emission Quenching Experiments 10
   4.3 Cyclovoltammetry experiment 13
   4.4 Miscellaneous 15
      Bromine effect on the reaction kinetics 15
      Giese-type addition 16
5. Photocatalyzed Suzuki-Miyaura reaction 16
      General procedure using haloarenes and triflates as substrates 16
      Tested substrates excluded from the study using bromo arene as starting material in batch 17
      General procedure using acylchlorides as substrates 17
6. Spectral data for new compounds 18
7. DFT calculations 53
   7.1. General information 53
   7.2 Results 54
   7.3 Energies and coordinates of calculated structures 58
References 73

1. General information

MATERIALS

Unless otherwise noted, all commercially available compounds were used as received without further purification.

INSTRUMENTATIONS

NMR spectra were recorded on a BRUKER Avance III HD 400 using the solvent peak as internal reference for the chemical shifts d of the reported ^{1}H and ^{13}C nuclei ($CDCl_3$: δ (1H) = 7.26, δ (^{13}C) = 77.16. Multiplicities of signals are reported as s (singlet), d (doublet), t (triplet), q (quartet), quint (quintet), sept (septet), m (multiplet) and combinations of these multiplicities. Coupling constants J are reported in Hz. All coupling constants J reported refer to JHH couplings, if not stated otherwise. Spectra of ^{13}C nuclei were recorded ^{1}H-decoupled. The applied solvents are specified in the descriptions of the corresponding experiments.

Product isolation was performed using silica (60, F254, Merck™), and TLC analysis was performed using Silica on aluminium foils TLC plates (F254, Supelco Sigma-Aldrich™) with visualization under ultraviolet light (254 nm) and appropriate TLC staining, mainly potassium permanganate and Seebach staining solution.

Cyclic voltammetry measurements were performed with Metrohm PGSTAT20 potentiostat/galvanostat equipped with a glassy carbon electrode as working electrode and platinum wire as counter electrode. Ag/AgCl (Silver wire in an aqueous 3 M KCl electrolyte) was used as pseudo reference electrode. A 0.1 M solution of tetrabutylammonium hexafluorophosphate was applied as supporting electrolyte. The scan rate was set to 150 mV/s. Solutions were degassed with argon prior to measurement and experiments were performed under an atmosphere of argon.

Stern-Volmer experiments were obtained on a FLS 920, Edinburgh Fluorescence Spectrometer.

Photochemical experiments were performed magnetically stirred in 10 mL glass test tubes with screw caps equipped with silicon septa. The tubes were irradiated with a blue Kessil light (456 nm, 40W) in an EvoluChem™ PhotoRedOx Box. To maintain a constant reaction temperature of 40°C, the fan of the setup was turned off (Figure S1).

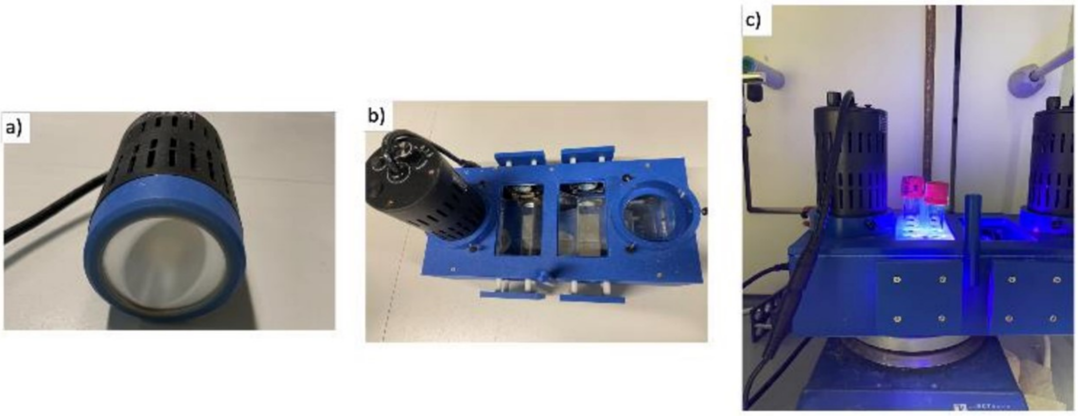

Figure S1: a) Kessil light; b,c) Set up using EvoluChem™ PhotoRedOx Box.

2. Reaction optimization

To a 10 mL reaction vial equipped with a stir bar was added methyl 4-bromobenzoate, **1a** (21.5 mg, 0.1 mmol, 1 equiv), cyclopentylboronic acid, **2a** (17.1 mg, 1.5 equiv), the appropriate photocatalyst (0.0025 mmol, 0.025 equiv if metal based; 0.005 mmol, 0.05 equiv if organic dye), the appropriate nickel complex (from 0.005 mmol, 0.05 equiv to 0.01 mmol, 0.1 equiv) and cesium carbonate (from 0.1 mmol, 1 equiv to 0.3 mmol, 3 equiv). The vial was sealed with a cap containing a TFE-lined silicone septa and was evacuated and purged with argon three times via an inlet needle. The vial was then charged with an appropriate anhydrous solvent (0.1M, 1 mL). After this, the vial was irradiated in the PhotoRedox BOX reactor with Kessil light 456 nm. The temperature of the reaction was maintained at approximately 40 °C. After 18 h, the solvent was evaporated and 3,4,5-trimethoxybenzaldehyde used as internal standard for ^{1}H NMR analysis.

Table S1: Solvent screening for photoredox-catalyzed Suzuki-Miyaura reaction

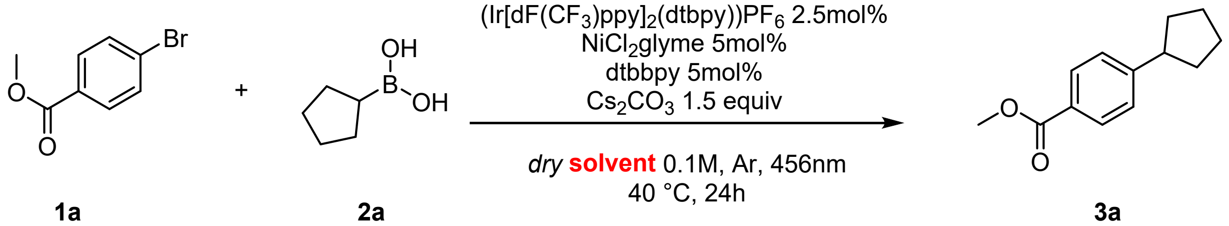

1a **2a** **3a**

Entry	Solvent	Yield%
**1**	**DMA** Dimethylacetamide	41
**2**	**DMF** Dimethylformamide	38
**3**	**DMSO** Dimethyl sulfoxide	60
**4**	**DCM** Dichloromethane	23
**5**	**DCE** Dichloroethane	23
**6**	**PhCl** Chlorobenzene	33
**7**	**CH_3Cl** Chloroform	n.r.
**8**	Acetone	18
**9**	1,4-dioxane	41
**10**	**MeOH** Methanol	n.r.
**11**	Toluene	25
**12**	**NMP** N-Methyl-2-pyrrolidone	17

* The amide based solvents (DMA, DMF, DMSO) are known to interact with BAs.

Table S2: Base screening for photoredox-catalyzed Suzuki-Miyaura reaction

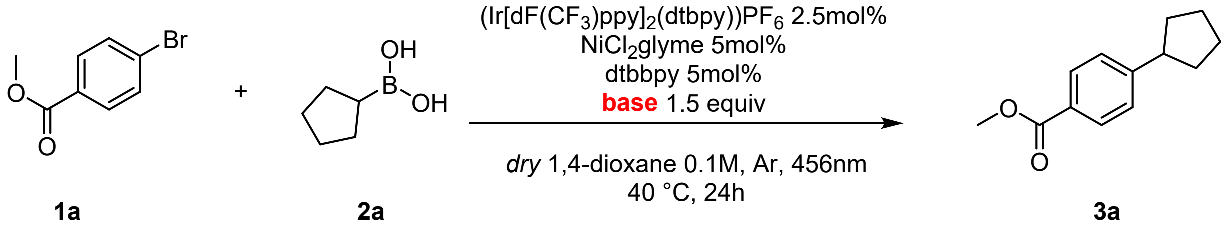

1a **2a** **3a**

Entry	Base	Yield%
**1**	Cs_2CO_3	41
**2**	Na_2CO_3	n.r.
**3**	K_2CO_3	n.r.
**4**	$CaCO_3$	n.r.
**5**	CsF	n.r.
**6**	CsOAc	n.r.
**7**	CsBr	traces
**8**	Na_2HPO_4	n.r.
**9**	K_3PO_4	38[a]
**10**	Cs_2CO_3 + 18-Crown-6	5[a]

a. Isolated yield.

Table S3: Optimization of Cs_2CO_3 equivalents

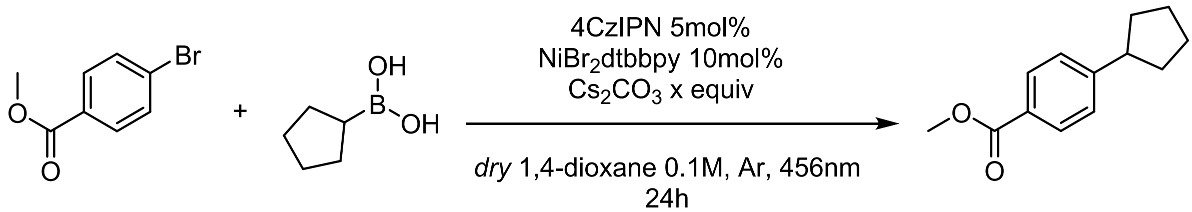

1a **2a** **3a**

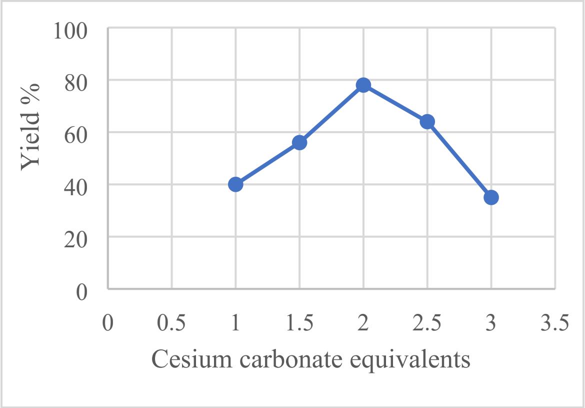

Entry	Equiv	Yield%*
**1**	1	40
**2**	1.5	56
**3**	2	78
**4**	2.5	64
**5**	3	35

* GC-MS yield using 3,4,5-trimethoxy benzene as internal standard.

Table S4: Ligand screening for photoredox-catalyzed Suzuki-Miyaura reaction

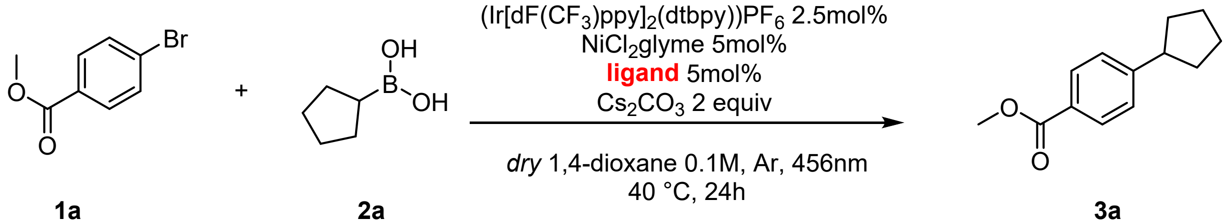

1a **2a** **3a**

Entry	Ligand	Yield%
**1**	dtbbpy	41
**2**	4,4′-Dimethyl-2,2′-dipyridyl	n.r.
**3**	2,2′-Bipyridyl	n.r.
**4**	neocuproine	14
**5**	phenantridine	n.r.
**6**	phenanthroline	20
**7**	bathophenanthroline	25

Table S5: Nickel catalyst screening for photoredox-catalyzed Suzuki-Miyaura reaction

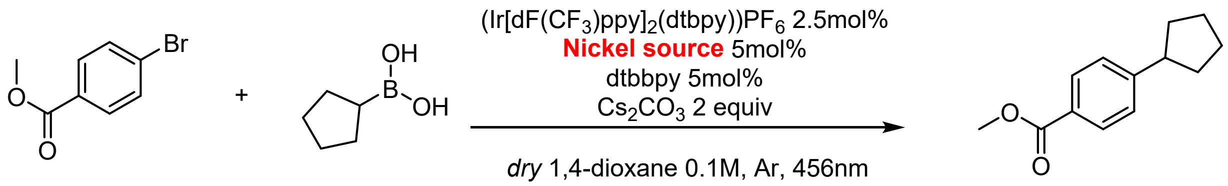

1a **2a** **3a**

Entry	Nickel catalyst	Yield%
**1**	$NiCl_2$ glyme	41
**2**	$NiCl_2 \cdot xH_2O$	44[a]
**3**	$NiBr_2$ glyme	63[a]
**4**	$NiBr_2 \cdot xH_2O$	65
**5**	$NiCl_2$ glyme + $ZnBr_2$	65
**6**	$NiBr_2$ glyme 10mol%	64[a]
**7**	$NiBr_2$ glyme 20mol%	68[a]

a. Isolated yield.

Table S6: Photocatalyst screening for photoredox-catalyzed Suzuki-Miyaura reaction

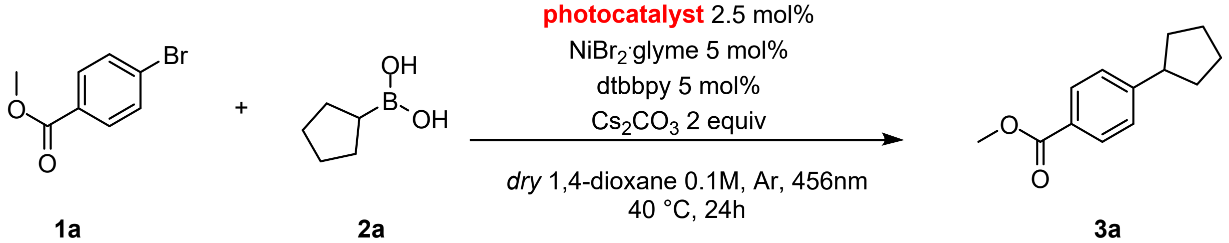

1a **2a** **3a**

Entry	Photocatalyst	Yield%
**1**	9-Mesityl-3,6-di-tert-butyl-10-phenylacridinium tetrafluoroborate	traces
**2**	9-Mesityl-methylacridinium perchlorate	Traces
**3**	4CzIPN 3mol%[a]	60
**4**	4CzIPN 5mol%[c]	78[b]
**5**	$[Ir(dtbbpy)(ppy)_2]PF_6$	53
**6**	$[Ir(dFCF_3ppy)_2(ppy)]PF_6$	57

**7**	(Ir[dF(CF_3)ppy]$_2$(dtbbpy))PF_6	63[b]

a. Nickel glyme **b.** isolated yield **c.** Nickel source 10 mol %, dtbbpy 10 mol%

Table S7: Additive screening for photoredox-catalyzed Suzuki-Miyaura reaction

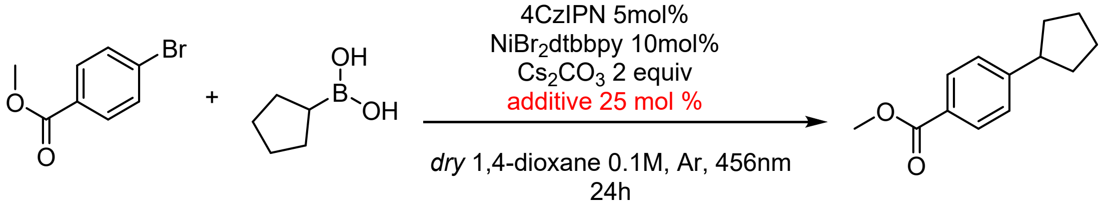

1a **2a** **3a**

**Entry**	**Additive**	**Yield%**
**1**	TBAI	n.r.
**2**	TBABr	90
**3**	TBACl	19
**4**	$ZnBr_2$	26
**5**	LiBr	26

Table S8: Organic base for photoredox-catalyzed Suzuki-Miyaura reaction

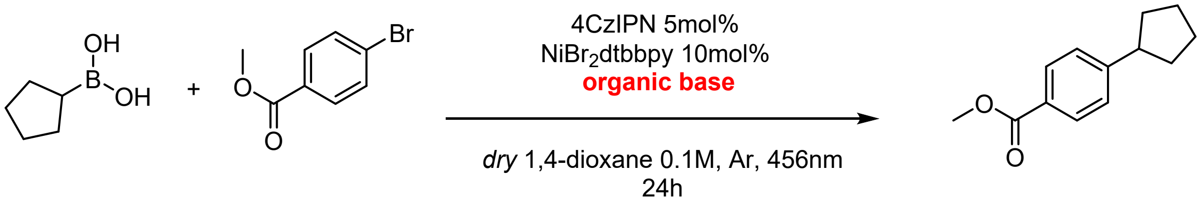

**Entry**	**Base**	**Yield%**
**1**	2,6-lutidine	n.d.
**2**	Et_3N	n.d.
**3**	3-quinoclidinol	65[*b]
**4**	Quinoclidinol	20[b]
**5**	DBU	10[c]
**6**	DIPEA	15[c]
**7**	TMG	68[c,d]
**8**	NaOtBu	40[c]
**9**	DABCO	n.d. (s.m.)
**10**	CyNMe	n.d.

a. 1 equiv. **b.** 2 equiv. **c.** 1.5 equiv **d.** (Ir[dF(CF_3)ppy]$_2$(dtbbpy))PF_6 *NMR yield 3,4,5-trimethoxybenzaldehyde used as internal standard.

3. $NiBr_2$·dtbbpy synthesis and characterization

To 250 mL round bottom flask, 5.0 g of nickel(II) bromide trihydrate (18.2 mmol, 1.0 equiv) and 9.95 g of 4,4'-di-tert-butyl-2,2'-bipyridine (36.3 mmol, 2.0 equiv) were added to 100 mL absolute ethanol (20 mL/g). The resulting green suspension was stirred under air at 23°C for 16h. The resulting solution was then concentrated to ~ 10 mL and 90 mL tert-butyl methyl ether (MTBE, 18 mL/g) was slowly added over 1h. The resulting suspension was aged with stirring for 1h then filtered, washed with 20 mL MTBE and dried under vacuum to yield 13.4 g of green solids (97% yield).

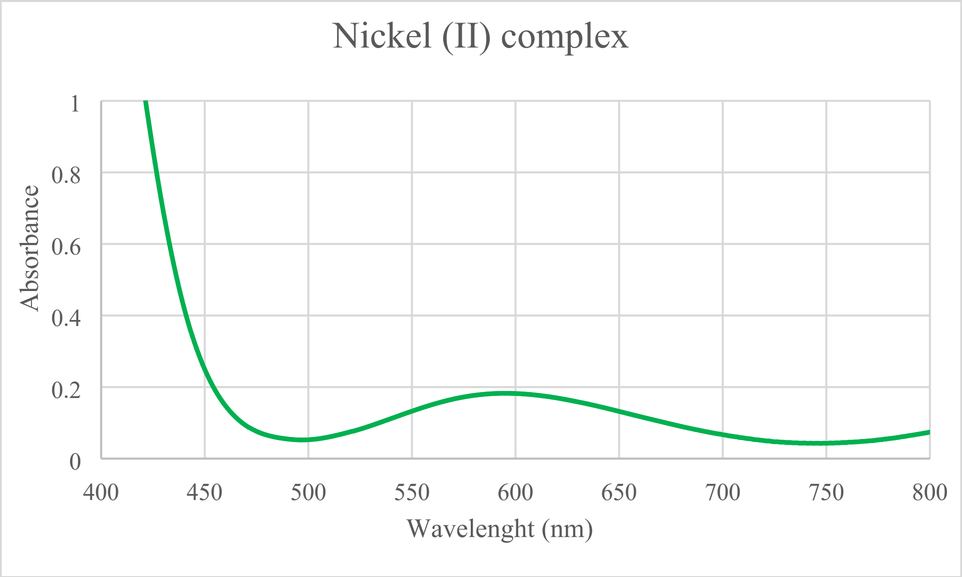

Figure S2. UV absorption spectrum for $NiBr_2$·dtbbpy solution (5mg/mL DMA).

4. Mechanistic investigations

4.1 NMR Experiments

Probing the role of Cs_2CO_3 in the reaction: To a 5.0 mL reaction vial equipped with a magnetic stirring bar phenethylboronic acid (15.0 mg, 0.10 mmol) and appropriate amount of Cs_2CO_3 were added. After performing three-cycles vacuum-argon, 0.5 mL of d3-Acetonitrile were added. The reaction mixtures were stirred for 4h, filtered and then transferred to a quartz NMR tube for ^{11}B NMR analysis.

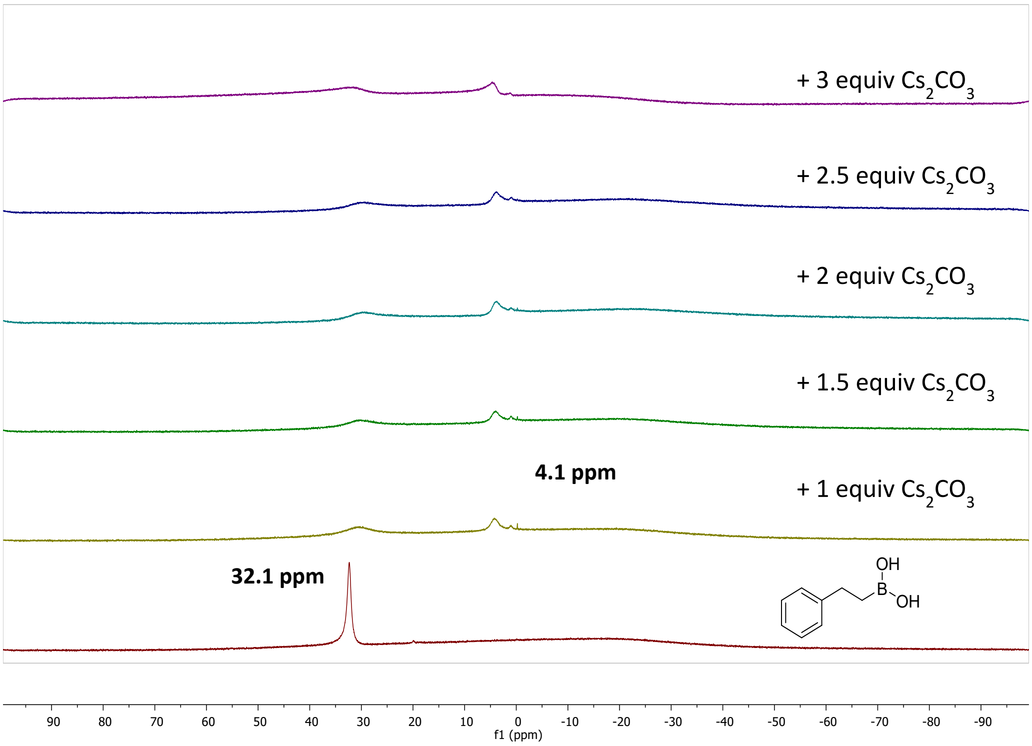

Figure S3: ^{11}B NMR analysis between phenethylboronic acid and Cs_2CO_3.

From Figure S3, it is evident that a new species is formed at 4.1 ppm after adding Cs_2CO_3, while the signal's intensity at 32.1 ppm decreases. Increasing the base amount does not result in an increased intensity of the new boron complex species at 4.1 ppm.

Complex between 1,4-dioxane and boronic acid: As shown in Figure S4, by increasing the amount of 1,4-dioxane, the signal of the protons bonded to the boronic acid's oxygens shifts from 4.1ppm to 4.8ppm. This indicates hydrogen bonding interactions between the boronic acid and the solvent.

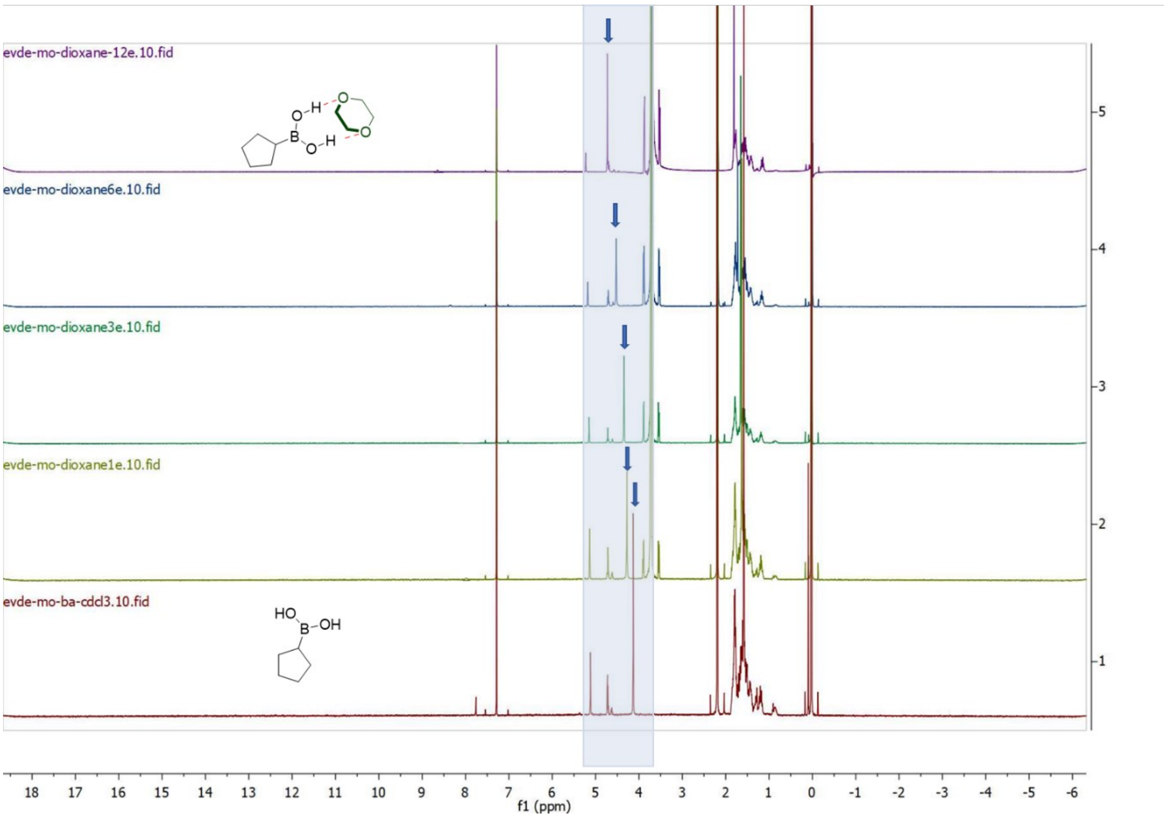

Figure S4: ^{1}H NMR analysis between cyclopentyl boronic acid and increasing amounts of 1,4-dioxane.

Detecting the presence of $B(OH)_3$ in the reaction mixture

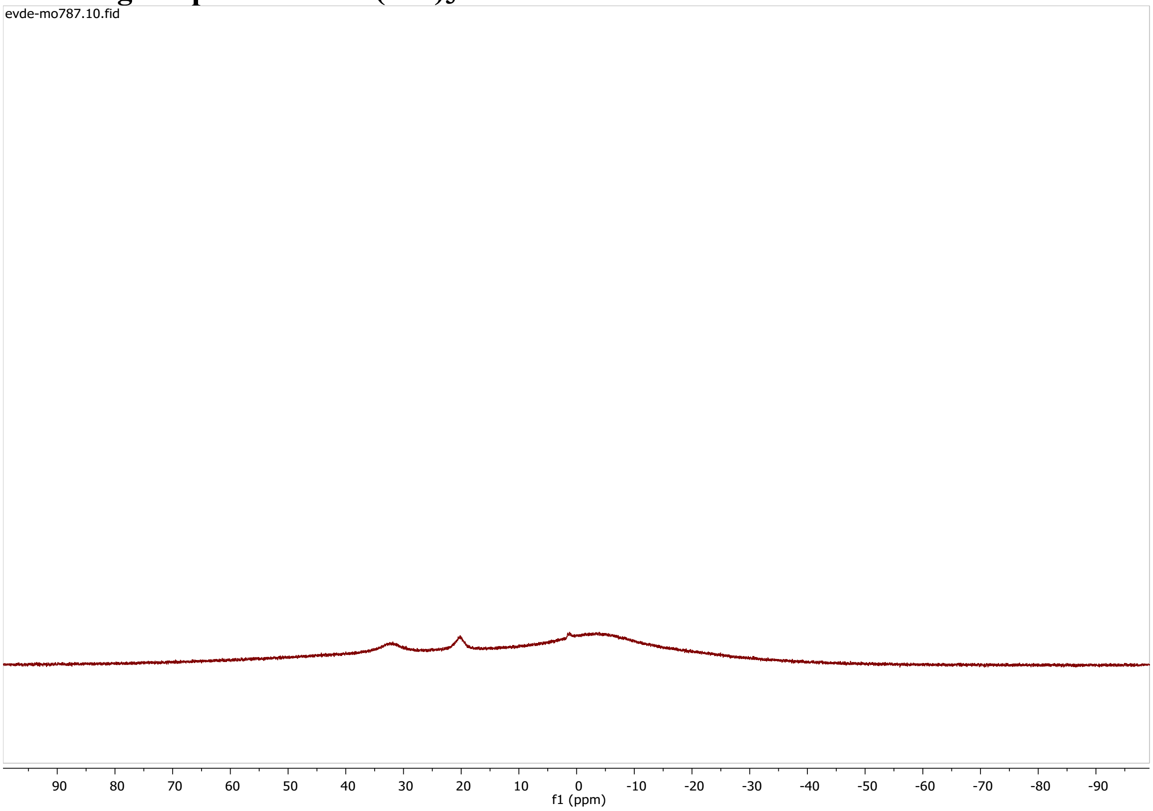

Figure S5: ^{11}B NMR spectrum. $B(OH)_3$ detection at 20 ppm. Broad signal in the spectrum arising from the tube.

4.2 Stern-Volmer Emission Quenching Experiments

Fluorescence measurements were obtained using UV-Quartz cuvettes (10 mm pathlength) obtained from Hellma Analytics (Cat#: 111-10-40) with home-made silicon septa. Excitation was performed at 435 nm; fluorescence spectra were obtained from 450- 800 nm at medium speed (excitation slit width: 2.5 nm; emission slit width: 5 nm). The relative intensity I_0/I was calculated as a function of quencher concentration, where I_0 is the luminescence intensity in the absence of quencher, while I is the intensity in the presence of the quencher. Before each measurement, the solutions were degassed for 60 seconds and kept under nitrogen atmosphere.

The following stock solutions were prepared:

4CzIPN solution (1.78 mM): To an oven-dried vial 4CzIPN (4.2 mg, 5.35 µmol) and acetonitrile were added (3 mL).

TBABr solution (3.10 mM): To an oven-dried 2-dram vial TBABr (6.00 mg, 18.6 µmol) and acetonitrile were added (6.00 mL).

$NiBr_2$·dtbbpy solution (2.01 mM): To an oven-dried vial Ni(II) complex (5mg, 10 µmol) and acetonitrile were added (5.00 mL).

Cs_2CO_3 + BA solution (3.10 mM): To an oven-dried vial Cs_2CO_3 (5.8 mg, 18.6 µmol), ciclopentylboronic acid (2.0 mg, 18.6 µmol) and acetonitrile were added (6.00 mL). The solution was stirred for one hour and filtered before the measurement.

1,4-dioxane + BA solution (3.10 mM): To an oven-dried vial 1,4-dioxane (6 µL, 74.4 µmol), ciclopentylboronic acid (2.0 mg, 18.6 µmol) and acetonitrile (6.00 mL) were added.

For each experiment, a 3 mL solution of 4CzIPN in acetonitrile 25µM has been prepared with increasing concentrations of quenchers.

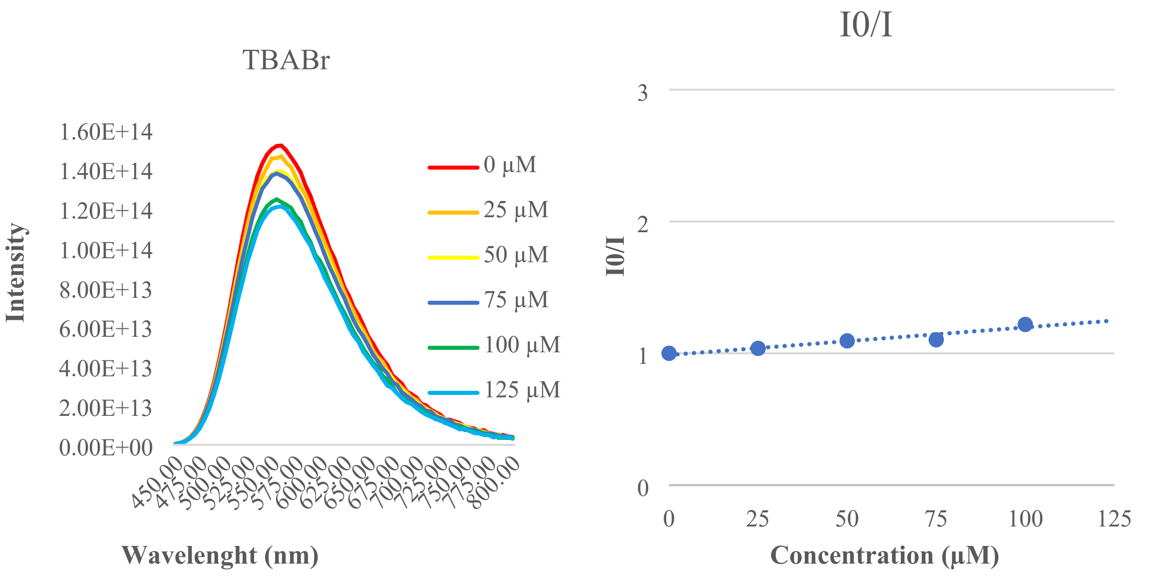

Figure S6: Fluorescence of 4CzIPN with TBABr.

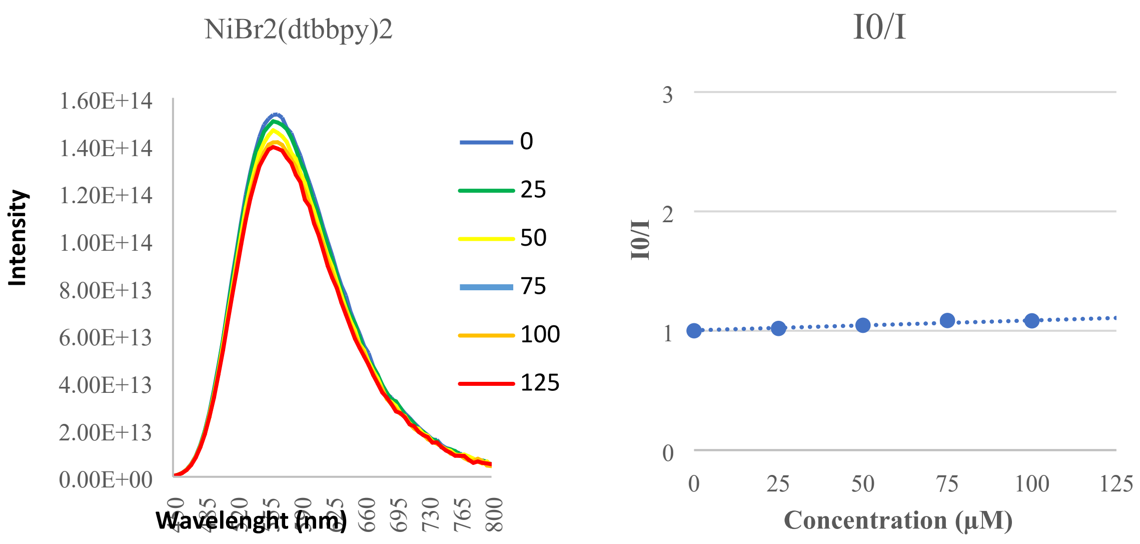

Figure S7: Fluorescence of 4CzIPN with $NiBr_2(dtbbpy)_2$.

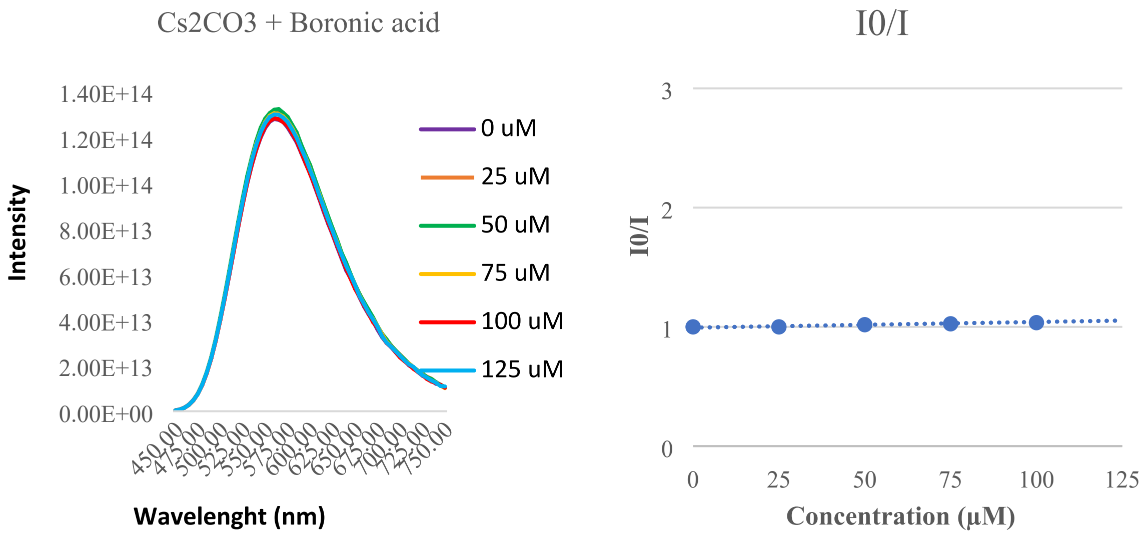

Figure S8: Fluorescence of 4CzIPN with Cs_2CO_3 and cyclopentyl boronic acid.

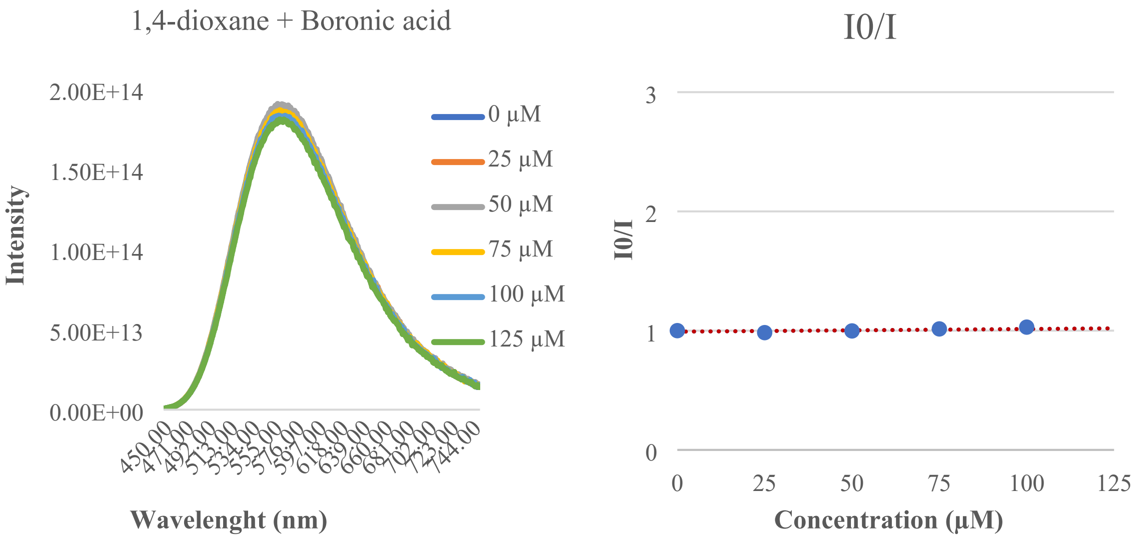

Figure S9: Fluorescence of 4CzIPN with 1,4-dioxane and cyclopentyl boronic acid.

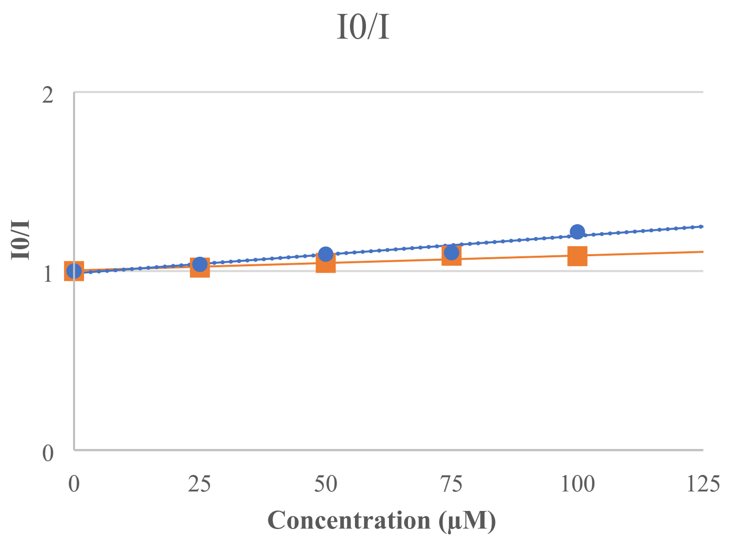

Figure S10: Stern Volmer plot for TBABr and $NiBr_2(dtbbpy)_2$ quenching experiments.

4.3 Cyclovoltammetry experiment

Cyclic voltammetry measurements were performed with Metrohm PGSTAT20 potentiostat/galvanostat equipped with a glassy carbon electrode as working electrode and platinum wire as counter electrode. Ag/AgCl (Silver wire in an aqueous 3 M KCl electrolyte) was used as pseudo reference electrode. A 0.1 M solution of tetrabutylammonium hexafluorophosphate was applied as supporting electrolyte. The scan rate was set to 150 mV/s. Solutions were degassed with argon prior to measurement and experiments were performed under an atmosphere of argon.

TBABr + boronic acid: Into an oven-dried vial nBu$_4$NPF$_6$ (1.0 mmol, 387 mg), phenethylboronic acid (0.1 mmol, 15 mg) and TBABr (0.25 mmol, 81 mg) were added. Three cycles vacuum-argon were applied. After the addition of *dry* acetonitrile (10 mL), the suspension was stirred for one hour, poured in the electrochemical cell, and degassed prior the measurement. The scan rate is 150 mV/s, ranging from 0 V to 2.5 V.

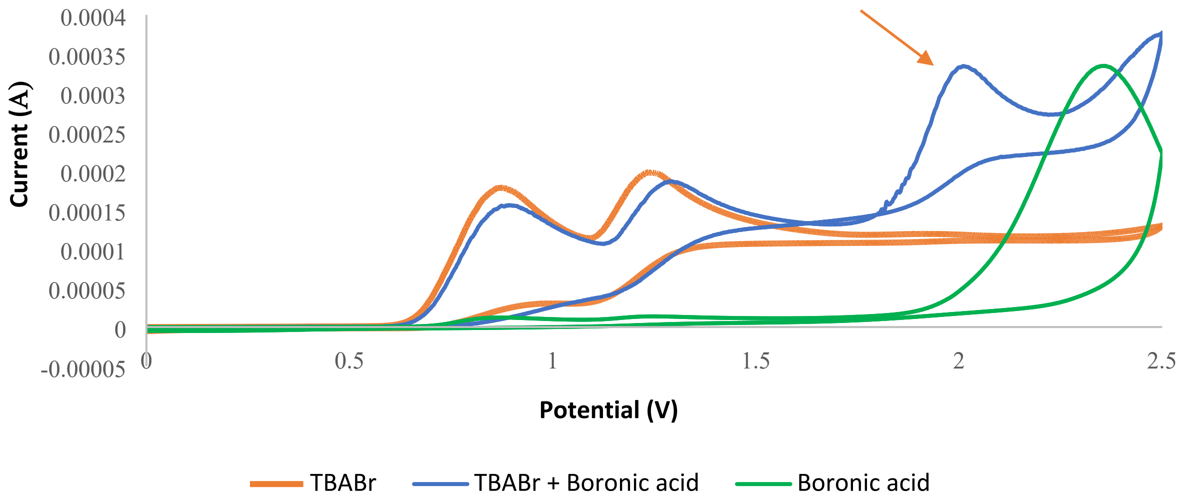

Cs_2CO_3 + boronic acid: Into an oven-dried vial were added *n*Bu_4NPF_6 (1.0 mmol, 387 mg), phenethylboronic acid (0.1 mmol, 15 mg) and Cs_2CO_3 (0.25 mmol, 81.4 mg). After applying three cycles vacuum-argon, *dry* acetonitrile (10 mL) was added, and the suspension was stirred for one hour. It was then filtered, poured in the electrochemical cell, and degassed prior the measurement. The scan rate is 150 mV/s, ranging from 0 V to 2.5 V.

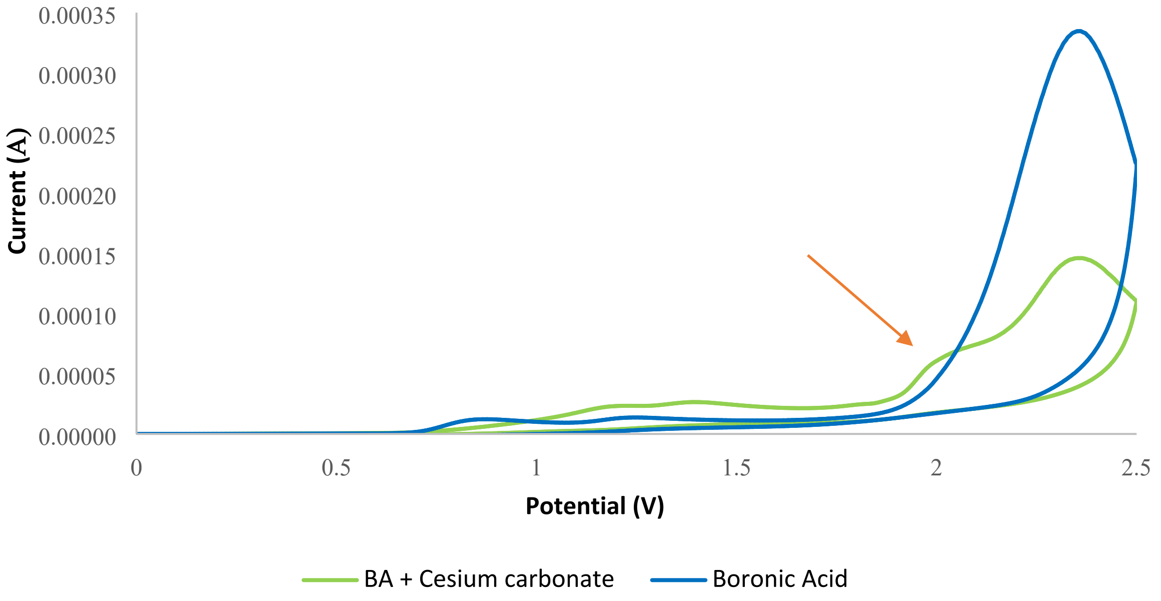

1,4-dioxane + boronic acid: Acetonitrile (10 mL) containing 1.0 mmol nBu_4NPF_6 (387 mg), 0.1 mmol phenethylboronic acid (15 mg) and 0.4 mmol of 1,4-dioxane (34 µL) were poured into the electrochemical cell. The scan rate is 150 mV/s, ranging from 0 V to 2.5 V.

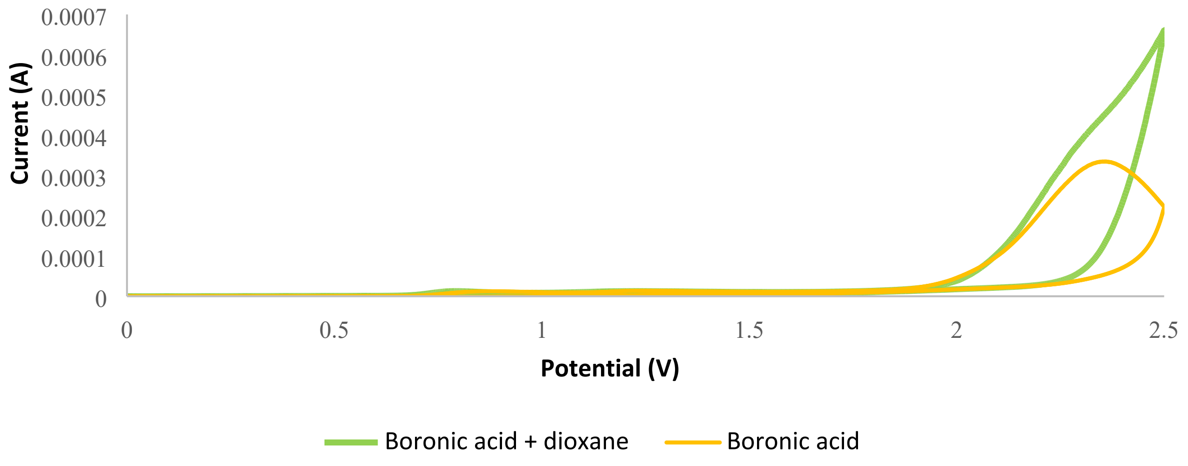

The same measurements performed using 1,4-dioxane as the major solvent (in a mixture with ACN in a ration of 7:3) did not lead to conclusive observations. The peak of the boronic acid could not be clearly detected, and the addition of Cs_2CO_3 did not lead to an appreciable observation of new or shifted peaks.

As shown in the first two graphs reported above, when mixing phenethyl boronic acid respectively with TBABr and Cs_2CO_3, a new peak is forming around 2, suggesting the formation of a complex between the boronic acid and the additive employed. Nonetheless, its redox potential lies out the potential window of 4CzIPN, thus excluding the possibility of SET mechanism between the new boronic acid species and the photocatalyst.

As a consequence, the cyclicvoltammetry data shown above suggest the positive interaction between TBABr and BA for radical generation.

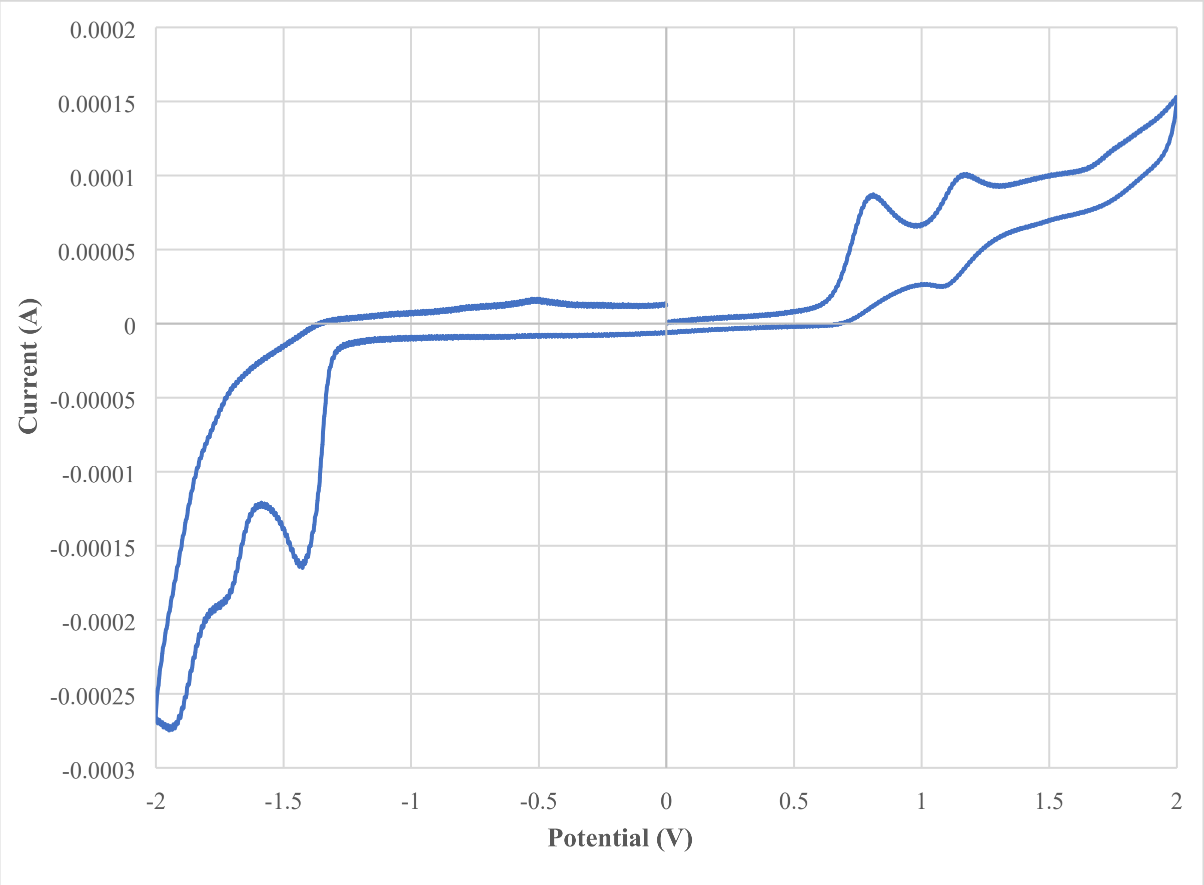

Figure S11: (dtbbpy)Ni(*o*-tolyl)Br ciclovoltammetry spectrum in CH_3CN.

Cyclic voltammogram of (dtbbpy)Ni(o-tolyl)Br shows an irreversible first oxidation at 0.65 V versus SCE in CH_3CN which corresponds to the NiII/NiIII redox couple and an irreversible first reduction at – 1.3 V *versus* SCE in CH_3CN which corresponds to the NiI /NiII redox couple. Remaining peaks could not be assigned due to the irreversible nature of the first oxidation and reduction.

4.4 Miscellaneous

Bromine effect on the reaction kinetics

To an oven-dried 10 mL screw-capped vial equipped with a stirring bar were added cyclopentylboronic acid (34.2 mg, 0.3 mmol), methyl 4-bromobenzoate (43 mg, 0.2 mmol), 4CzIPN (7.9 mg, 0.01 mmol), $NiBr_2$·dtbbpy (9.7 mg, 0.02 mmol) Cs_2CO_3 (130.3 mg, 0.4 mmol) TBABr (16.1 mg, 0.05 mmol). The vial was capped, and three cycles vacuum/argon were performed before adding anhydrous degassed 1,4-dioxane (0.2 mL, 0.1 M). The reaction

mixture was stirred for 24 hours under 456 nm light irradiation using PhotoBOX reactor (Kessil light, LED power = 100%, fan speed = absent, stirring = 600 rpm). Different portions of the crude mixture at different times were collected. Product yields were measured by GC-MS analysis of the crude mixture in the presence of internal standard (3,4,5-trimethoxybenzene). The same experiment was also conducted without TBABr to observe bromine anion effect on reaction progress.

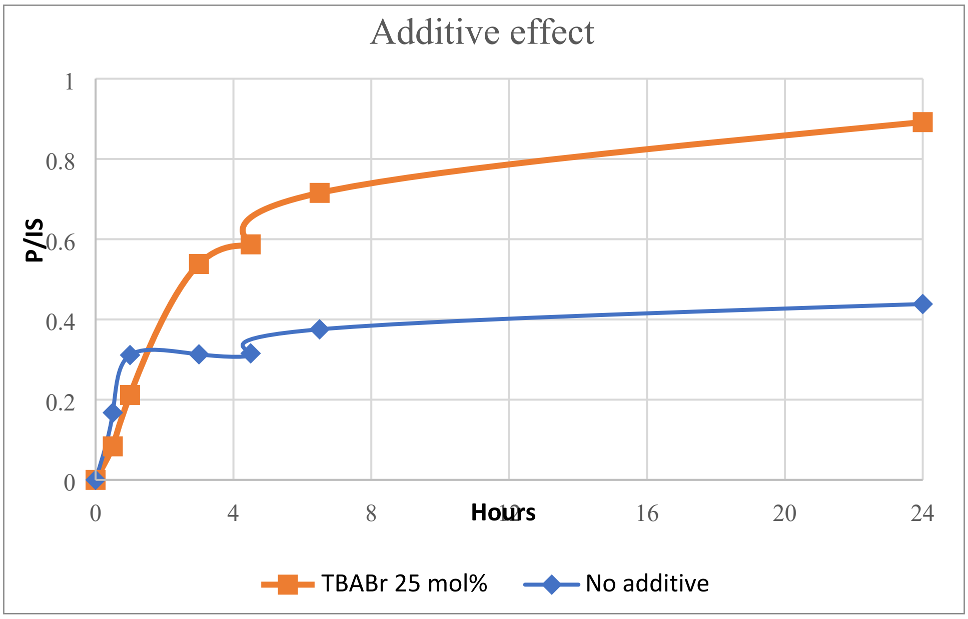

Figure S12: bromo anion effect on reaction's kinetics. P = product, IS = internal standard

These preliminary results show the benefits on the reaction kinetics by using TBABr as additive.

Giese-type addition

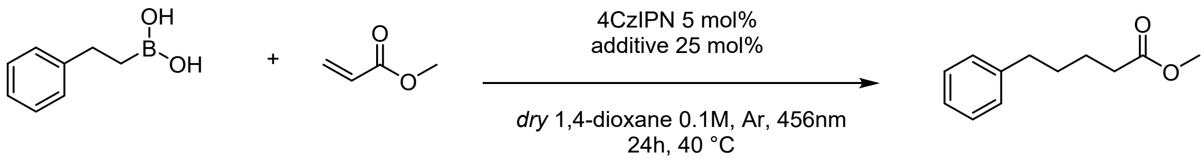

Entry	Solvent	Additive	Isolated yield%
1	1,4-dioxane	-	0
2	1,4-dioxane	TBABr	60

To further confirm the involvement of TBABr in radical formation from alkyl boronic acid, a Giese-type addition was performed. As shown in entry 2, no product is observed in the absence

of TBABr. This suggests a possible radical transfer mechanism from bromine radical and boronic acid with subsequent radical formation.

5. Photocatalyzed Suzuki-Miyaura reaction

Reaction in the presence of electron rich aryl iodides

When performing the reaction with electron rich aryl bromides, a moderate yield was observed. Due to the difficult oxidative addition in the presence of electron-rich aryl bromides, the reaction was attempted with electron-rich aryl iodides.

The reaction proved to be equally effective using aryl iodides, and especially in the case of electron rich species, good yields could be obtained.

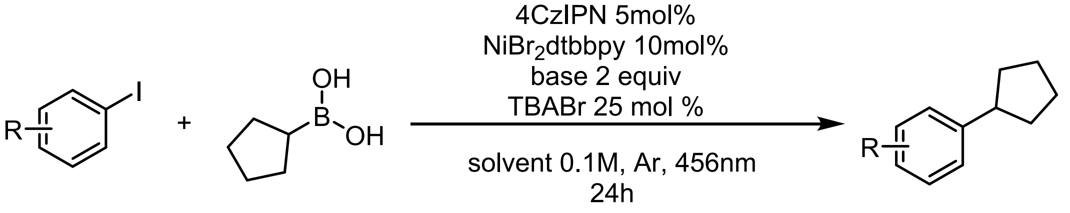

1a **2a** **3a**

Aryl iodide	Yield (%)
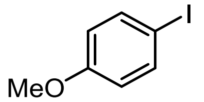	70%
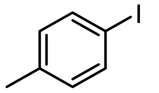	70%

1-cyclopentyl-4-methoxybenzene

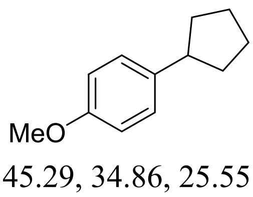

^{1}H NMR (600 MHz, Chloroform-*d*) δ 7.17 (d, *J* = 8.6 Hz, 2H), 6.84 (d, *J* = 8.7 Hz, 2H), 3.79 (s, 3H), 2.95 (m, 1H), 1.84 – 1.76 (m, 2H), 1.72 – 1.64 (m, 3H), 1.60 – 1.51 (m, 3H).

^{13}C NMR (151 MHz, $CDCl_3$) δ 157.77, 138.69, 128.05, 113.77, 55.40, 45.29, 34.86, 25.55.

1-cyclopentyl-4-methylbenzene

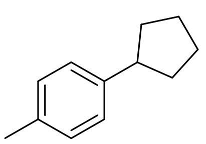

^{1}H NMR (600 MHz, Chloroform-d) δ 7.15 (d, J = 8.0 Hz, 1H), 7.11 (d, J = 7.9 Hz, 1H), 3.01 – 2.93 (m, 1H), 2.33 (s, 3H), 2.09 – 2.04 (m, 2H), 1.84 – 1.78 (m, 2H), 1.72 – 1.65 (m, 2H), 1.62 – 1.55 (m, 2H).

^{13}C NMR (151 MHz, CDCl3) δ 143.59, 135.23, 129.05, 127.11, 45.72, 34.82, 25.64, 21.10.

General procedure using haloarenes and triflates as substrates

To a 10 mL reaction vial equipped with a stir bar were added bromobenzoate or triflate (0.2 mmol, 1 equiv), alkyl boronic boronic acid, (0.3 mmol, 1.5 equiv), 4CzIPN (8 mg, 0.01 mmol, 0.05 equiv), $NiBr_2{\cdot}dtbbpy$ (9.7 mg, 0.02 mmol, 0.1 equiv), Cs_2CO_3 (130 mg, 0.4 mmol, 2 equiv), and TBABr (16 mg, 0.05 mmol, 0.25 equiv). The vial was sealed with a cap containing a TFE-lined silicone septa and was evacuated and purged with argon three times via an inlet needle. The vial was then charged with an appropriate anhydrous solvent (0.1M, 2 mL. After this, the vial was irradiated in the PhotoRedox BOX reactor equipped with one Kessil light 456 nm. The temperature of the reaction was maintained at approximately 40 °C. After 24 h, the solvent was evaporated and purification via flash columns chromatography performed.

Tested substrates excluded from the study using bromo arene as starting material in batch

Failed substrates

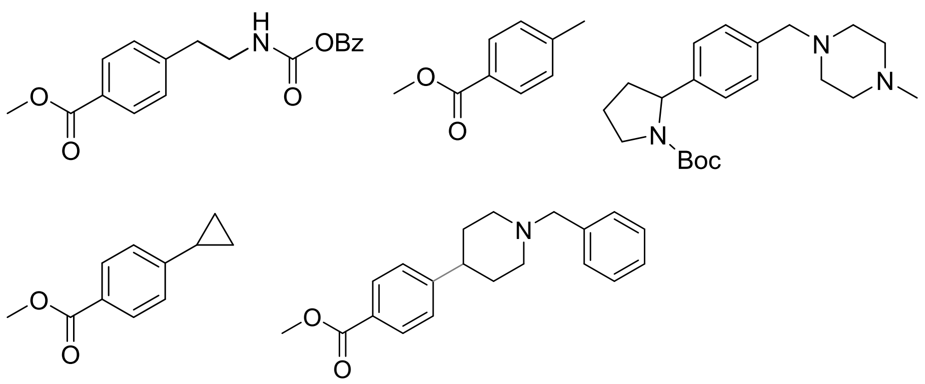

Poor performing substrates

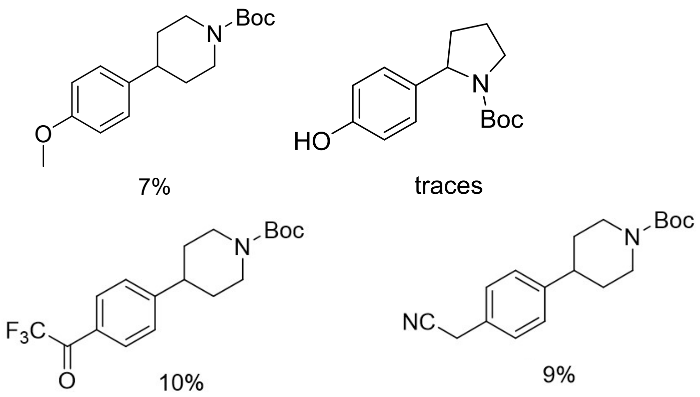

Failing tested boronic esters

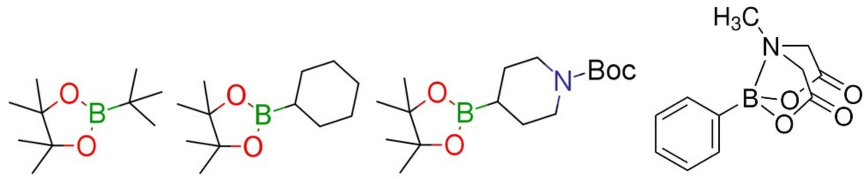

General procedure using acylchlorides as substrates

To a 10 mL reaction vial equipped with a stir bar were added acyl chloride (if solid) (0.3 mmol, 1 equiv), alkyl boronic boronic acid, (0.45 mmol, 1.5 equiv),), $[Ir(dFCF_3ppy)_2(bpy)]PF_6$ (6 mg, 0.006 mmol, 0.02 equiv), $NiCl_2$·glyme (2.6 mg, 0.012 mmol, 0.04 equiv) dtbbpy (3.2 mg, 0.012 mmol, 0.04 equiv), Cs_2CO_3 (195 mg, 0.6 mmol, 2 equiv), The vial was sealed with a cap containing a TFE-lined silicone septa and was evacuated and purged with argon three times via an inlet needle. The vial was then charged with anhydrous THF (0.1M, 3 mL and acyl chloride (if liquid). After this, the vial was irradiated in the PhotoRedox BOX reactor equipped with one Kessil light 456 nm. The temperature of the reaction was maintained at approximately 40 °C. After 24 h, the solvent was evaporated and purification via flash columns chromatography performed.

6. Spectral data for new compounds

Methyl 4-cyclopentylbenzoate (3a)

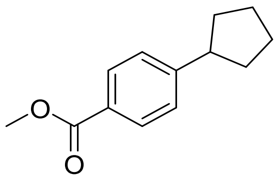

The product was isolated by flash chromatography (2% ethyl acetate/heptane) as a yellow oil (37 mg, 90%). The spectroscopic data of this compound are consistent with the data reported in the literature.[1]

^{1}H NMR (400 MHz, Chloroform-*d*) δ 7.95 (d, *J* = 8.4 Hz, 2H), 7.30 (d, *J* = 8.2 Hz, 2H), 3.90 (s, 3H), 3.12 – 2.95 (m, 1H), 2.14 – 2.02 (m, 2H), 1.87 – 1.78 (m, 2H), 1.74 – 1.67 (m, 2H), 1.67 – 1.56 (m, 2H).

^{13}C NMR (101 MHz, Chloroform-*d*) δ 167.32, 152.34, 129.73, 127.76, 52.06, 46.10, 34.62, 25.70.

Tert-butyl 2-(4-methoxycarbonylphenyl)pyrrolidine-1-carboxylate (3b)

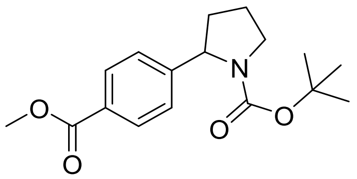

The product was isolated by flash chromatography (10% ethyl acetate/heptane) as a yellow oil (55 mg, 90%). The product was obtained as a mixture of two rotamers (R1:R2 = 2.3:1). The spectroscopic data of this compound are consistent with the data reported in the literature.[2]

^{1}H NMR (600 MHz, Chloroform-*d*) δ 7.97 (d, *J* = 8.3 Hz, **2H**, R1 + R2), 7.23 (d, *J* = 8.4 Hz, **2H**, R1 + R2), 4.97 (brm, R2), 4.79 (brm, R1) (**1H** for R1 + R2), 3.90 (s, **3H**, R1 + R2), 3.65 – 3.52 (m, **2H**, R1 + R2), 2.40 – 2.27 (m, **1H**, R1 + R2), 1.96 – 1.76 (m, **3H**, R1 + R2), 1.44 (s, R2), 1.16 (s, R1) (**9H** for R1 and R2).

^{13}C NMR (151 MHz, Chloroform-*d*) δ 167.16, 154.56, 150.75, 129.95, 129.75, 128.65, 125.61, 79.63, 61.35, 60.73, 52.16, 47.51, 47.28, 36.07, 28.26, 23.40, 22.82.

Methyl 4-isopropylbenzoate (3c)

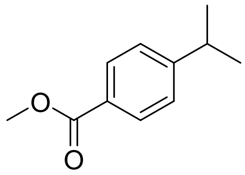

The product was isolated by flash chromatography (2% ethyl acetate/heptane) as a yellow oil (14 mg, 40%). The spectroscopic data of this compound are consistent with the data reported in the literature.[3]

^{1}H NMR (400 MHz, Chloroform-*d*) δ 7.96 (d, J = 8.4 Hz, 2H), 7.29 (d, J = 8.2 Hz, 2H), 3.90 (s, 3H), 2.96 (p, J = 6.9 Hz, 1H), 1.27 (d, J = 7.0 Hz, 6H).

^{13}C NMR (101 MHz, Chloroform-*d*) δ 167.32, 154.45, 129.87, 127.91, 126.61, 52.09, 34.39, 23.84.

Methyl 4-cyclobutylbenzoate (3d)

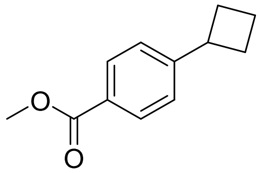

The product was isolated by flash chromatography (3% ethyl acetate/heptane) as a white solid (37 mg, 97%). The spectroscopic data of this compound are consistent with the data reported in the literature.[1]

^{1}H NMR (400 MHz, Chloroform-*d*) δ 7.96 (d, J = 8.9 Hz, 2H), 6.81 (d, J = 8.9 Hz, 2H), 4.78 – 4.63 (m, 1H), 3.87 (s, 3H), 2.55 – 2.36 (m, 2H), 2.27 – 2.09 (m, 2H), 1.95 – 1.80 (m, 1H), 1.78 – 1.66 (m, 1H).

^{13}C NMR (101 MHz, Chloroform-*d*) δ 167.02, 161.55, 131.71, 122.54, 114.67, 71.85, 51.95, 30.63, 13.43.

1,1-Dimethylethyl 4-[4-(methoxycarbonyl)phenyl]-1-piperidinecarboxylate (3e)

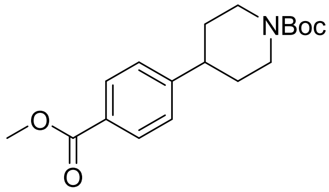

The product was isolated by flash chromatography (10% ethyl acetate/heptane) as a light-yellow solid (64 mg, 99%). The spectroscopic data of this compound are consistent with the data reported in the literature.[4]

^{1}H NMR (400 MHz, Chloroform-*d*) δ 7.97 (d, *J* = 8.4 Hz, 2H), 7.26 (d, *J* = 8.4 Hz, 2H), 4.25 (brs, 2H), 3.89 (s, 3H), 2.80 (t, *J* = 12.8 Hz, 2H), 2.70 (tt, *J* = 12.2, 3.6 Hz, 1H), 1.86 – 1.78 (m, 2H), 1.62 (qd, *J* = 12.6, 4.3 Hz, 2H), 1.47 (s, 9H).

^{13}C NMR (101 MHz, Chloroform-*d*) δ 167.11, 154.91, 151.17, 130.00, 128.45, 126.95, 79.66, 52.13, 42.92, 33.00, 28.59.

Methyl 4-(2-phenylethyl)benzoate (3f):

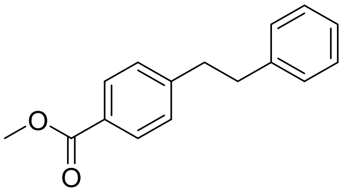

The reaction was performed on a 0.1 mmol scale and the product was isolated by flash chromatography (3% ethyl acetate/heptane) as a yellow oil (15 mg, 62%). The spectroscopic data of this compound are consistent with the data reported in the literature.[5]

^{1}H NMR (400 MHz, Chloroform-*d*) δ 8.01 – 7.95 (m, 2H), 7.32 – 7.16 (m, 7H), 3.93 (s, 3H), 3.05 – 2.90 (m, 4H).

^{13}C NMR (101 MHz, Chloroform-*d*) δ 167.28, 147.30, 141.26, 129.82, 129.68 – 128.53 (m), 128.07, 126.22, 52.12, 38.02, 37.59.

Methyl 4-(2-methylpropyl)benzoate (3g)

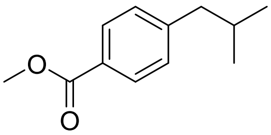

The product was isolated by flash chromatography (2% ethyl acetate/heptane) as a yellow liquid (16 mg, 42%). The spectroscopic data of this compound are consistent with the data reported in the literature.[6]

^{1}H NMR (400 MHz, Chloroform-*d*) δ 7.95 (d, *J* = 8.3 Hz, 2H), 7.21 (d, *J* = 8.4 Hz, 2H), 3.90 (s, 3H), 2.52 (d, *J* = 7.2 Hz, 2H), 1.89 (hept, *J* = 6.8 Hz, 1H), 0.91 (s, 3H), 0.89 (s, 3H).

^{13}C NMR (101 MHz, Chloroform-*d*) δ 167.37, 147.42, 129.60, 129.25, 127.83, 52.08, 45.54, 30.26, 22.45.

Methyl 4-ethylbenzoate (3h)

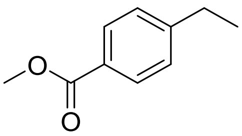

The product was isolated by flash chromatography (2% ethyl acetate/heptane) as a yellow liquid (15 mg, 45%). The spectroscopic data of this compound are consistent with the data reported in the literature.[7]

^{1}H NMR (400 MHz, Chloroform-*d*) δ 7.96 (d, *J* = 8.3 Hz, 2H), 7.26 (d, *J* = 8.5 Hz, 1H), 3.90 (s, 3H), 2.70 (q, *J* = 7.6 Hz, 2H), 1.26 (t, *J* = 7.6 Hz, 3H).

^{13}C NMR (101 MHz, Chloroform-*d*) δ 167.34, 149.88, 131.86, 131.26, 129.84, 128.02, 127.78, 52.09, 29.09, 15.35.

Methyl 4-butylbenzoate (3i)

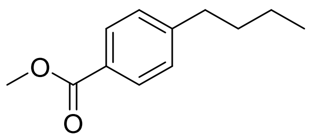

The product was isolated by flash chromatography (2% ethyl acetate/heptane) as a yellow liquid (16 mg, 41%). The spectroscopic data of this compound are consistent with the data reported in the literature.[7]

^{1}H NMR (400 MHz, Chloroform-*d*) δ 7.95 (d, *J* = 8.3 Hz, 2H), 7.24 (d, *J* = 8.3 Hz, 2H), 3.90 (s, 3H), 2.88 – 2.52 (m, 2H), 1.77 – 1.48 (m, 2H), 1.35 (dq, *J* = 14.6, 7.3 Hz, 2H), 0.93 (t, *J* = 7.4 Hz, 3H).

^{13}C NMR (101 MHz, Chloroform-*d*) δ 167.35, 148.62, 129.75, 128.57, 127.75, 52.08, 35.84, 33.40, 22.45, 14.03.

Methyl 4-(but-3-en-1-yl)benzoate (3j)

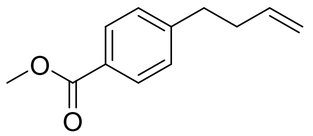

The product was isolated by flash chromatography (2% ethyl acetate/heptane) as a yellow oil (8 mg, 21%). The spectroscopic data of this compound are consistent with the data reported in the literature.[8]

^{1}H NMR (400 MHz, Chloroform-*d*) δ 8.00 – 7.95 (m, 2H), 7.30 – 7.25 (m, 2H), 5.85 (ddt, *J* = 16.9, 10.2, 6.6 Hz, 1H), 5.10 – 4.98 (m, 2H), 3.93 (s, 3H), 2.79 (dd, *J* = 8.7, 6.8 Hz, 2H), 2.45 – 2.38 (m, 2H).

^{13}C NMR (101 MHz, Chloroform-*d*) δ 167.31, 147.50, 137.63, 129.81, 128.64, 128.01, 115.51, 52.12, 35.51, 35.21, 29.85.

1,1-Dimethylethyl 2-[4-(trifluoromethyl)phenyl]-1-pyrrolidinecarboxylate (4a)

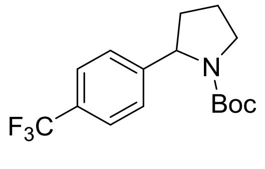

The product was isolated by flash chromatography (from 5% to15 % ethyl acetate/heptane) as a yellow oil (42 mg, 66%). The product was obtained as a mixture of two rotamers (R1:R2 =

2:1). The spectroscopic data of this compound are consistent with the data reported in the literature.[9]

^{1}H NMR (400 MHz, Chloroform-*d*) δ 7.55 (d, *J* = 8.2 Hz, **2H**, R1+R2), 7.28 (d, *J* = 8.0 Hz, **2H**, R1+R2), 4.98 (brm, R2), 4.81 (brm, R1) (**1H** for R1+R2), 3.68-3.52 (m, **2H**, R1+R2), 2.41-2.27 (m, **1H**, R1+R2), 1.94-1.70 (m, **3H**, R1+R2), 1.45 (s, R2) and 1.18 (s, R1) (**9H**, R1+R2).

^{13}C NMR (101 MHz, Chloroform-*d*) δ 156.63, 149.15, 128.91 (d, *J* = 32.3 Hz), 125.48 (d, *J* = 60.1 Hz), 120.86, 118.45, 79.70, 61.17 and 60.64 (R1 + R2) 47.53 and 47.28 (R1 + R2), 36.07 and 35.12 (R1 + R2), 28.59 and 28.25 (R1 + R2), 23.67 and 23.33 (R1 + R2).

***tert*-butyl 2-(4-acetylphenyl)pyrrolidine-1-carboxylate (4b)**

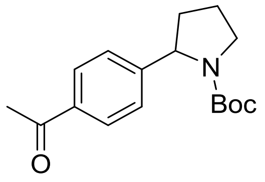

The product was isolated by flash chromatography (from 5% to 15% ethyl acetate/heptane) as a yellow oil (57 mg, 99%). The product was obtained as a mixture of two rotamers (R1:R2 = 2:1). The spectroscopic data of this compound are consistent with the data reported in the literature.[10]

^{1}H NMR (600 MHz, Chloroform-*d*) δ 7.89 (d, *J* = 8.3 Hz, **2H**, R1 + R2), 7.25 (d, *J* = 8.3 Hz, **2H**, R1 + R2), 4.95 (brm, R2), 4.80 (brm, R1) (**1H** for R1 + R2), 3.68- 3.48 (m, **2H**, R1 + R2), 2.58 (s, **3H**, R1 + R2), 2.37 - 2.28 (m, **1H**, R1 + R2), 1.91 - 1.76 (m, **3H**, R1 + R2), 1.44 (s, R2), 1.16 (s, R1) (**9H** for R1 + R2).

^{13}C NMR (151 MHz, Chloroform-*d*) δ 197.86, 154.52, 150.91, 135.83, 128.54, 125.74, 79.61, 61.27 and 60.78 (R1 +R2), 47.52 and 47.24 (R1 +R2), 36.00, 28.57 and 28.25 (R1 +R2), 26.69, 23.35 and 22.79 (R1 +R2).

***tert*-butyl 2-(4-fluorophenyl)pyrrolidine-1-carboxylate (4c)**

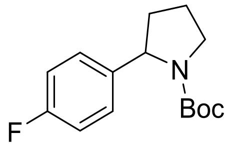

The product was isolated by flash chromatography (from 5% to 10% ethyl acetate/heptane) as a yellow oil (53 mg, 99%). The product was obtained as a mixture of two rotamers (R1:R2 = 2:1). The spectroscopic data of this compound are consistent with the data reported in the literature.[11]

^{1}H NMR (400 MHz, Chloroform-*d*) δ 7.16 – 7.09 (m, **2H**, R1 + R2), 7.02 – 6.91 (m, **2H**, R1 + R2), 4.92 (brm, R2), 4.74 (brm, R1) (**1H** for R1 + R2), 3.64 – 3.62 (m, **2H**, R1 + R2), 2.36 – 2.26 (m, **1H**, R1 + R2), 1.94 – 1.74 (m, **3H**, R1 + R2), 1.46 (s, R2), 1.22 – 1.18 (m, R1) (**9H** for R1 and R2).

^{13}C NMR (101 MHz, Chloroform-*d*) δ 162.95, 160.53, 154.66, 127.05, 115.15 and 114.94 (R1 + R2), 79.47, 60.86, 47.22, 36.23 and 35.57 (R1 + R2), 32.07, 29.84, 22.83.

^{19}F NMR (400 MHz, Chloroform-*d*) – 116.87.

***tert*-butyl 2-(4-2,2,2-trifluoroacetophenone)pyrrolidine-1-carboxylate (4d)**

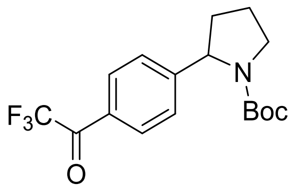

The product was isolated by flash chromatography (from 5% to 10% ethyl acetate/heptane) as a yellow oil (21 mg, 31%). The product was obtained as a mixture of two rotamers (R1:R2 = 1.5:1).

^{1}H NMR (400 MHz, Chloroform-*d*) δ 8.01 (d, *J* = 8.0 Hz, **2H**, R1 + R2), 7.35 (d, *J* = 8.4 Hz, **2H**, R1 + R2), 4.99 – 4.97 (brm, R2), 4.84 – 4.81 (brm, R1) (**1H** for R1 + R2), 3.66 – 3.63 (m, **2H**, R1 + R2), 2.41 - 2.34 (m, **1H**, R1 + R2), 1.93 – 1.78 (m, **3H**, R1 + R2), 1.45 (s, R2), 1.17 (s, R1) (**9H** for R1 and R2).

^{13}C NMR (101 MHz, Chloroform-*d*) δ 180.05 (q, *J* = 34.8 Hz), 154.31, 153.80, 152.84, 132.61, 130.54, 130.31, 128.44, 126.18, 121.07, 118.17, 115.27, 112.38, 79.81, 61.23 and 60.75 (R1 + R2), 47.21, 35.85 and 34.72 (R1 + R2), 28.55 and 28.44 (R1 + R2), 23.68 and 23.32 (R1 + R2).

^{19}F NMR (400 MHz, Chloroform-*d*) – 71.35.

1,1-Dimethylethyl 2-(4-cyanomethyl)-1-pyrrolidinecarboxylate (4e)

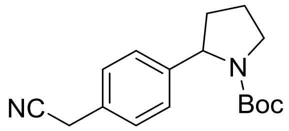

The product was isolated by preparative TLC (20% ethyl acetate/heptane) as a yellow oil (50mg, 87%). The product was obtained as a mixture of two rotamers (R1:R2 = 1.5:1).

^{1}H NMR (600 MHz, Chloroform-*d*) δ 7.27 – 7.25 (m, **2H**, R1 + R2), 7.18 (d, J = 7.9 Hz, **2H**, R1 + R2), 4.93 (brm, R2), 4.77 (brm, R1) (**1H** for R1 + R2), 3.72 (d, J = 12.6 Hz, **2H**), 3.65 – 3.46 (m, **2H**, R1 + R2), 2.40 – 2.24 (m, **1H**, R1 + R2), 1.91 – 1.83 (m, **2H**, R1 + R2), 1.80 – 1.74 (m, **1H**, R1 + R2), 1.45 (s, R2), 1.19 (s, R1) (**9H** for R1 and R2).

^{13}C NMR (151 MHz, Chloroform-*d*) δ 154.64, 145.42, 128.14, 127.90, 126.43, 118.11, 79.52, 60.80 (d, J = 77.2 Hz), 47.21, 36.12, 28.32, 23.41.

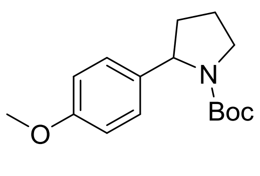

***tert* - Butyl - 2 - (4 - methoxyphenyl) pyrrolidine – 1 -carboxylate (4f)**

The product was isolated by flash chromatography eluting with 5% EtOAc/hexanes to afford the title compound as a colorless oil (20 mg, 36% yield). The product was obtained as a mixture of two rotamers (R1:R2 = 2:1).

^{1}H NMR (400 MHz, $CDCl_3$): δ 7.08 (d, J = 8.3 Hz, **2H**, R1 + R2), 6.83 (d, J = 8.3 Hz, **2H**, R1 + R2), 4.90 (br. m, R2), 4.71 (br. m, R1) (**1H** for R1 + R2), 3.79 (s, **3H** for R1 + R2)), 3.66–3.42 (m, **2H**, R1 + R2), 2.34–2.17 (m, **1H**, R1 + R2), 1.95–1.74 (m, **1H**, R1 + R2), 1.45 (br. s, R2), 1.20 (br. s, R1) (**9H** for R1 and R2).

^{13}C NMR (101 MHz, $CDCl_3$): δ 158.34 (R1 + R2), 154.78 (R1 + R2), 137.43 (R2), 136.40 (R1), 126.72 (R1 + R2), 113.88 (R1), 113.56 (R2), 79.25 (R1 + R2), 60.86 (R2), 60.25 (R1), 55.40 (R1 + R2), 47.39 (R1), 47.12 (R2), 36.19 (R2), 35.01 (B), 28.65 (B), 28.35 (A), 23.59 (B), 23.27 (A).

The spectroscopic properties of this compound are consistent with data reported in the literature.

***tert*-butyl 2-([1,1'-biphenyl]-4-yl)pyrrolidine-1-carboxylate (4g)**

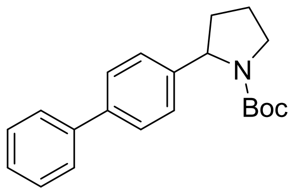

The product was isolated by flash chromatography (from 10% to 15% ethyl acetate/heptane) as a white solid (65 mg, quantitative yield). The product was obtained as a mixture of two rotamers (R1:R2 = 2:1). The spectroscopic data of this compound are consistent with the data reported in the literature.[12]

^{1}H NMR (400 MHz, Chloroform-*d*) δ 7.67 – 7.49 (m, **4H**, R1 + R2), 7.47 – 7.39 (m, **2H**, R1 + R2), 7.37 – 7.28 (m, **1H**, R1 + R2), 7.28 – 7.20 (m, **2H**, R1 + R2), 5.07 – 4.96 (brm, R2), 4.89 – 4.76 (brm, R1) (**1H** for R1 + R2), 3.73 – 3.48 (m, **2H**, R1 + R2), 2.43 – 2.23 (m, **1H**, R1 + R2), 2.02 – 1.78 (m, **3H**, R1 + R2), 1.47 (s, R2), 1.21 (s, R1) (**9H** for R1 and R2).

^{13}C NMR (151 MHz, Chloroform-*d*) δ 154.74, 144.38, 141.03, 139.50, 128.85, 127.32, 127.24 (d, J = 3.9 Hz), 127.10, 126.92, 126.13, 125.91, 79.40, 72.58, 67.23, 66.53, 61.21, 60.59, 47.21, 36.13, 34.98, 28.50 (d, J = 57.6 Hz), 23.37.

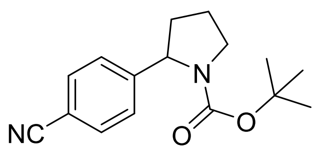

tert-Butyl 2-(4-cyanophenyl)pyrrolidine-1-carboxylate (4h)

The product was isolated by flash chromatography eluting with 7% EtOAc/hexanes to afford the title compound as a foam (27 mg, 50% yield). The product was obtained as a mixture of two rotamers (R1:R2 = 1.5:1).

^{1}H NMR (400 MHz, CDCl3) δ 7.60 (d, J = 8.0 Hz, **2H**, R1 + R2), 7.28 (d, J = 8.0 Hz, **2H**, R1 + R2), 4.94 (brm, R2), 4.78 (brm, R1) (**1H** for R1 + R2), 3.64-3.60 (m, **2H**, R1 + R2),

2.38-2.36 (m, **1H**, R1 + R2), 1.89-1.87 (m, **2H**, R1 + R2), 1.77- 1.75 (m, **1H**, R1 + R2), 1.45 (s, R2), 1.17 (s, R1) (**9H** for R1 and R2).

^{13}C NMR (101 MHz, CDCl3) δ 154.6, 154.3, 150.9, 149.9, 132.4, 132.3, 126.4, 119.1, 110.5, 79.8, 61.3, 60.8, 47.6, 47.3, 36.0, 34.9, 4

The spectroscopic properties of this compound are consistent with data reported in the literature.

***tert*-butyl 2-(5-(trifluoromethyl)pyridin-2-yl)pyrrolidine-1-carboxylate (4i)**

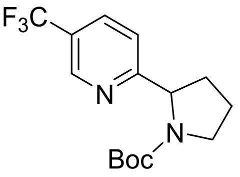

The product was isolated by preparative TLC (30% ethyl acetate/heptane) as a yellow oil (54 mg, 85%). The product was obtained as a mixture of two rotamers (R1:R2 = 1.5:1). The spectroscopic data of this compound are consistent with the data reported in the literature.[13]

^{1}H NMR (600 MHz, Chloroform-*d*) δ 8.80 (s, **1H**, R1 + R2), 7.86 (t, *J* = 9.7 Hz, **1H**, R1 + R2), 7.31 (t, *J* = 8.3 Hz, **1H**, R1 + R2), 5.07 – 5.00 (brm, R2), 4.93 – 4.91 (brm, R1) (**1H** for R1 + R2), 3.69 – 3.49 (m, **2H**, R1 + R2), 2.46 – 2.29 (m, **1H**, R1 + R2), 2.08 – 1.94 (m, **1H**, R1 + R2), 1.91 (q, *J* = 6.8 Hz, **2H**, R1 + R2), 1.45 (s, R2), 1.20 (s, R1) (**9H** for R1 + R2).

^{13}C NMR (151 MHz, Chloroform-*d*) δ 168.06, 166.92, 154.84, 154.45, 146.45, 146.22, 133.76, 133.51, 124.98, 124.76, 120.09, 119.61, 79.85, 62.88 and 62.31 (R1+R2), 47.63 and 47.28 (R1+R2), 34.39 and 33.13 (R1+R2), 28.61 and 28.31 (R1+R2), 24.05 and 23.41 (R1+R2).

^{19}F NMR (400 MHz, Chloroform-*d*) – 62.19.

***tert*-butyl 2-(pyridine-3-yl)pyrrolidine-1-carboxylate (4j)**

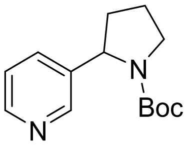

The product was isolated by flash chromatography (from 10% to 20% ethyl acetate/heptane) as a pale-yellow liquid (32 mg, 64%). The product was obtained as a mixture of two rotamers (R1:R2 = 2:1). The spectroscopic data of this compound are consistent with the data reported in the literature.

^{1}H NMR (400 MHz, Chloroform-*d*): δ 8.47 (s, **1H**, R1 + R2), 8.52 – 8.42 (m, **1H**, R1 + R2), 7.52 –7.46 (m, **1H**, R1 + R2), 7.23 (dd, J = 7.9, 4.8 Hz, **1H**, R1 + R2), 5.13 – 4.92 (m, R2), 4.85 –4.70 (m, R1) (**1H** for R1 + R2), 3.71 – 3.37 (m, **2H**, R1 + R2), 2.57 – 2.24 (m, **1H**, R1 + R2), 2.05 – 1.68 (m, **3H**, R1 + R2), 1.45 (s, R2), 1.20 (s, R2) (**9H** for R1 + R2).

^{13}C NMR (101 MHz, Chloroform-*d*): δ 154.2,147.9, 147.7, 147.3, 140.3, 139.2, 133.0, 132.9, 123.1, 79.5, 59.1, 58.7, 47.1, 47.0, 35.8, 34.5, 28.3, 28.31, 23.4, 23.2.

***tert*-butyl 2-(6-methylpyridin-2-yl)pyrrolidine-1-carboxylate (4k)**

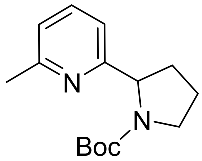

The product was isolated by flash chromatography (from 10% to 20% ethyl acetate/heptane) as a yellow solid (24 mg, 45%). The product was obtained as a mixture of two rotamers (R1:R2 = 2:1). The spectroscopic data of this compound are consistent with the data reported in the literature.[14]

^{1}H NMR (400 MHz, Chloroform-*d*) δ 7.49 (t, *J* = 7.7 Hz, **1H**, R1 + R2), 6.95 (dd, *J* = 17.4, 7.7 Hz, **2H**, R1 + R2), 5.03 – 4.92 (brm, R2), 4.89 – 4.76 (brm, R1) (**1H** for R1 + R2), 3.69 – 3.53 (m, **2H**, R1 + R2), 2.51 (s, **3H**, R1 + R2), 2.40 – 2.27 (m, **1H**, R1 + R2), 1.94 – 1.78 (m, **3H**, R1 + R2), 1.45 (s, R2), 1.19 (s, R1) (**9H** for R1 + R2).

^{13}C NMR (101 MHz, Chloroform-*d*) δ 163.36, 157.60, 154.74, 136.57, 121.11, 116.39, 79.36, 63.09 and 62.45 (R1+R2), 47.47 and 47.14 (R1+R2), 34.43, 28.65 and 28.30 (R1+R2), 24.54, 23.22.

***tert*-Butyl-2-(pyrimidin-5-yl)pyrrolidine-1-carboxylate (4l)**

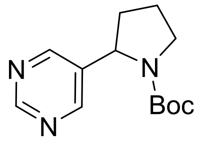

The product was isolated by flash chromatography (from 10% to 15% ethyl acetate/heptane) as a yellow solid (36 mg, 72%). The product was obtained as a mixture of two rotamers (R1:R2 = 1.4:1). The spectroscopic data of this compound are consistent with the data reported in the literature.[15]

^{1}H NMR (400 MHz, Chloroform-*d*) δ 9.08 (s, **1H**, R1 + R2), 8.56 (s, **2H**, R1 + R2), 4.91 (brs, R2), 4.82 – 4.68 (brm, R1), (**1H** for R1 + R2), 3.70 – 3.37 (m, **2H**, R1 + R2), 2.45 – 2.25 (m, **1H**, R1 + R2), 1.99 – 1.77 (m, **3H**), 1.42 (s, R2), 1.20 (s, R1) (**9H** for R1 + R2).

^{13}C NMR (101 MHz, Chloroform-*d*) δ 157.48, 154.76, 137.91, 136.94, 80.24, 57.40, 56.95, 47.21, 35.70, 34.33, 29.77, 29.43, 28.38, 23.83, 23.51.

***tert*-butyl 2-(6-methylpyridinecarboxilate-2-yl)pyrrolidine-1-carboxylate (4m)**

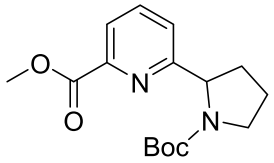

The product was isolated by flash chromatography (from 10% to 30% ethyl acetate/heptane) as a yellow solid (22 mg, 36%). The product was obtained as a mixture of two rotamers (R1:R2 = 1.5:1).

^{1}H NMR (600 MHz, Chloroform-*d*) δ 7.97 – 7.89 (m, **1H**, R1 + R2), 7.79 – 7.67 (m, **1H**, R1 + R2), 7.32 (d, *J* = 7.9 Hz, **1H**, R1 + R2), 5.09 – 5.02 (brm, R2), 4.98 – 4.95 (brm, R1), (**1H** for R1 + R2), 3.98 – 3.85 (m, **3H**, R1 + R2), 3.63 – 3.43 (m, **2H**, R1 + R2), 2.42 – 2.25 (m, **1H**, R1 + R2), 1.98 – 1.91 (m, **1H**, R1 + R2), 1.88 – 1.75 (m, **2H**, R1 + R2), 1.40 (s, R2), 1.13 (s, R1) (**9H** for R1 + R2).

^{13}C NMR (151 MHz, Chloroform-d) δ 165.88, 164.59, 163.34, 154.57 (d, J = 21.9 Hz), 147.48 (d, J = 39.8 Hz), 137.22, 123.16 (d, J = 11.9 Hz), 122.70, 79.56, 62.89, 62.21, 52.83 (d, J = 19.6 Hz), 47.27 (d, J = 44.5 Hz), 34.54, 33.18, 28.34 (d, J = 47.1 Hz), 23.73, 23.19.

HRMS (ESI$^+$): [M+H]$^+$ calculated for $\mathbf{C_{16}H_{23}N_2O_4}$ 307,1652 found: 307,1640.

***tert*-butyl 2-(dibenzo[b,d]furan-2-yl)pyrrolidine-1-carboxylate (4n)**

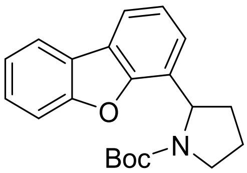

The product was isolated by flash chromatography (from 5% to 15% ethyl acetate/heptane) as a pale-yellow solid (56 mg, 83%). The product was obtained as a mixture of two rotamers (R1:R2 = 2.3:1).

^{1}H NMR (600 MHz, Chloroform-*d*) δ 7.95 (d, *J* = 7.7 Hz, **1H**, R1 + R2), 7.83 (d, *J* = 7.6 Hz, **1H**, R1 + R2), 7.61 – 7.56 (m, **1H**, R1 + R2), 7.48 – 7.43 (m, **1H**, R1 + R2), 7.37 – 7.31 (m, **1H**, R1 + R2), 7.29 (t, *J* = 7.5 Hz, **1H**, R1 + R2), 7.25 – 7.19 (m, **1H**, R1 + R2), 5.56 – 5.51 (brm, R2), 5.40 – 5.36 (brm, R1) (**1H** for R1 + R2), 3.81 – 3.57 (m, **2H**, R1 + R2), 2.46 (dq, *J* = 22.9, 15.4, 11.4 Hz, **1H**, R1 + R2), 2.12 – 1.89 (m, **2H**, R1 + R2), 1.83 (dd, *J* = 6.7, 3.5 Hz, **1H**, R1 + R2), 1.47 (s, R2), 1.08 (s, R1) (**9H** for R1 + R2).

^{13}C NMR (151 MHz, Chloroform-*d*) δ 156.21, 154.68, 153.17, 129.07, 124.38, 124.15, 123.73, 122.79, 122.66, 120.77, 119.05, 118.91, 111.82, 79.31, 79.01, 56.61, 47.07, 34.58, 28.69, 28.19, 23.98, 23.72.

HRMS (ESI$^+$): [M+Na]$^+$ calculated for $\mathbf{C_{21}H_{23}NaNO_3}$ 360,1570 found: 360,1560.

***tert*-Butyl 2-(2-quinolinyl)pyrrolidine-1-carboxilate (4o)**

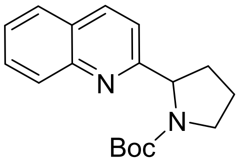

The product was isolated by flash chromatography (from 10% to 30% ethyl acetate/heptane) as a yellow solid (76 mg, 85%). The product was obtained as a mixture of two rotamers (R1:R2 = 2.3:1). The spectroscopic data of this compound are consistent with the data reported in the literature.[16]

^{1}H NMR (400 MHz, Chloroform-*d*) δ 8.14 – 8.08 (m, **1H**, R1 + R2), 8.03 (d, *J* = 8.5 Hz, **1H**, R1 + R2), 7.83 – 7.74 (m, **1H**, R1 + R2), 7.72 – 7.66 (m, **1H**, R1 + R2), 7.54 – 7.46 (m, **1H**, R1 + R2), 7.32 (d, *J* = 8.5 Hz, **1H**, R1 + R2), 5.16 (brs, R2), 5.07 – 4.98 (brmm, R1) (**1H** for R1 + R2), 3.70 (q, *J* = 10.7, 8.8 Hz, **2H**, R1 + R2), 2.55 – 2.35 (m, **1H**, R1 + R2), 2.07 – 1.83 (m, **3H**, R1 + R2), 1.46 (s, R2), 1.11 (s, R1), (**9H** for R1 + R2).

^{13}C NMR (151 MHz, Chloroform-*d*) δ 164.45, 154.78, 147.62, 136.63, 129.64, 129.05, 127.65, 127.19, 126.09, 117.85, 79.57, 63.78, 63.09, 47.69, 34.80, 29.83, 28.65, 23.71.

HRMS (ESI$^+$): [M+H]$^+$ calculated for $\mathbf{C_{18}H_{23}N_2O_2}$ 299,1754 found: 299,1759.

***tert*-butyl 2-(p-tolyl)pyrrolidine-1-carboxylate (4p)**

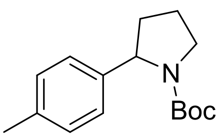

The product was isolated by flash chromatography (from 10% to 20% ethyl acetate/heptane) as a pale-yellow liquid (8 mg, 17%). The product was obtained as a mixture of two rotamers (R1:R2 = 2:1). The spectroscopic data of this compound are consistent with the data reported in the literature.[14]

^{1}H NMR (500 MHz, Chloroform-*d*) δ 7.10 (d, J = 7.6 Hz, **2H**, R1 + R2), 7.04 (d, J = 8.1 Hz, **2H**, R1 + R2), 5.00 – 4.67 (m, **1H**, R1 + R2), 3.69 – 3.42 (m, **2H**, R1 + R2), 2.32 (m, **4H**), 1.96 – 1.74 (m, **3H**), 1.45 (s, R2), 1.19 (s, R1) (**9H** for R1 + R2).

^{13}C NMR (125 MHz, Chloroform-*d*) δ 154.7, 142.0, 135.9, 129.0, 128.7, 125.4, 79.2, 61.0, 60.6, 47.0, 36.0, 34.8, 28.5, 28.2, 23.1, 21.0.

(4-(tert-Butyl)phenyl)(cyclopentyl)methanone (4q)

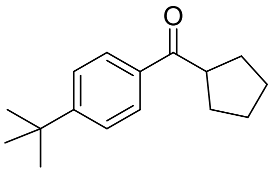

The product was isolated by flash chromatography (5% ethyl acetate/heptane) as a colourless oil (30 mg, 0.13 mmol, 43% yield). The spectroscopic data of this compound are consistent with the data reported in the literature.[17]

^{1}H NMR (500 MHz, Chloroform-*d*) δ 7.95 (d, J = 8.5 Hz, **2H**), 7.50 (d, J = 8.5 Hz, **2H**), 3.76-3.70 (m, **1H**), 1.96-1.92 (m, **4H**), 1.79-1.64 (m, **4H**), 1.37 (s, **9H**).

^{13}C NMR (125 MHz, Chloroform-*d*) δ 202.5, 156.4, 134.3, 128.4, 125.5, 46.3, 35.1, 31.1, 30.0, 26.3.

Methyl 4-(cyclopentanecarbonyl)benzoate (4r)

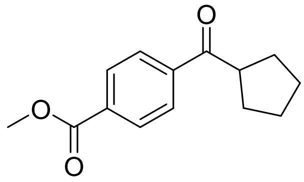

The product was isolated by flash chromatography (5% ethyl acetate/heptane) as a white solid (27 mg, 0.12 mmol, 39% yield). The spectroscopic data of this compound are consistent with the data reported in the literature.[18]

^{1}H NMR (400 MHz, Chloroform-*d*) δ 8.13 – 8.11 (m, **2H**), 8.03 – 8.00 (m, **2H**), 3.95 (s, **3H**), 3.72 (ddd, J = 15.7, 8.5, 7.2 Hz, **1H**), 1.98 – 1.88 (m, **4H**), 1.76 – 1.64 (m, **4H**).

^{13}C NMR (101 MHz, Chloroform-*d*) δ 202.4, 166.5, 140.4, 133.7, 129.9, 128.5, 52.5, 46.9, 30.0, 26.4.

(4-Chlorophenyl)(cyclopentyl)methanone (4s)

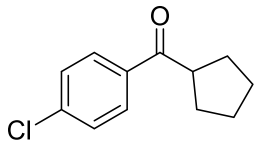

The product was isolated by flash chromatography (3% ethyl acetate/heptane) as a colourless oil (11 mg, 0.05 mmol, 18% yield). The spectroscopic data of this compound are consistent with the data reported in the literature.[19]

^{1}H NMR (600 MHz, Chloroform-*d*) δ 7.91 (d, J 8.7 Hz, **2H**), 7.42 (d, J 8.8 Hz, **2H**), 3.66 (quintet, J 8.1 Hz, **1H**), 1.72 (m, **2H**), 1.91 (m, **4H**), 1.66 (m, **2H**).

^{13}C NMR (150 MHz, Chloroform-*d*) δ 201.5, 139.1, 135.2, 129.9, 128.8, 46.3, 29.9, 26.3.

cyclopentyl(p-tolyl)methanone (4t)

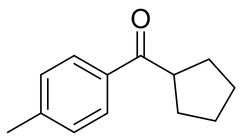

The product was isolated by flash chromatography (3% ethyl acetate/heptane) as a colourless oil (34 mg, 0.18 mmol, 60% yield). The spectroscopic data of this compound are consistent with the data reported in the literature.[20]

^{1}H NMR (600 MHz, CDCl3) δ 7.88 (d, J = 7.8 Hz, **2H**), 7.25 (d, J = 7.8 Hz, **2H**), 3.72– 3.67 (m, **1H**), 2.41 (s, **3H**), 1.95 – 1.86 (m, **4H**), 1.76 – 1.69 (m, **2H**), 1.67 – 1.61 (m, **2H**).

^{13}C NMR (151 MHz, CDCl3) δ 202.5, 143.4, 134.4, 129.2, 128.6, 46.2, 30.0, 26.3, 21.6.

^{1}H NMR spectrum of **3a**

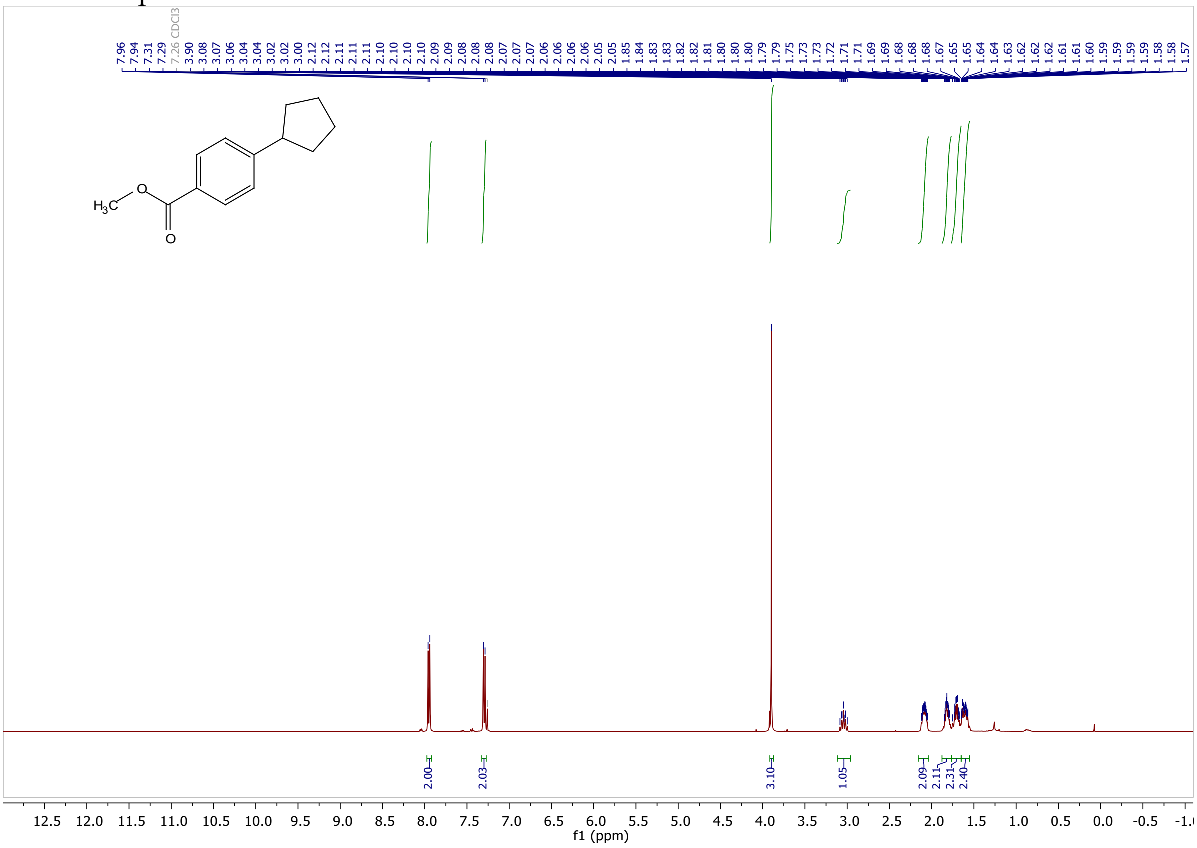

^{13}C NMR spectrum of **3a**

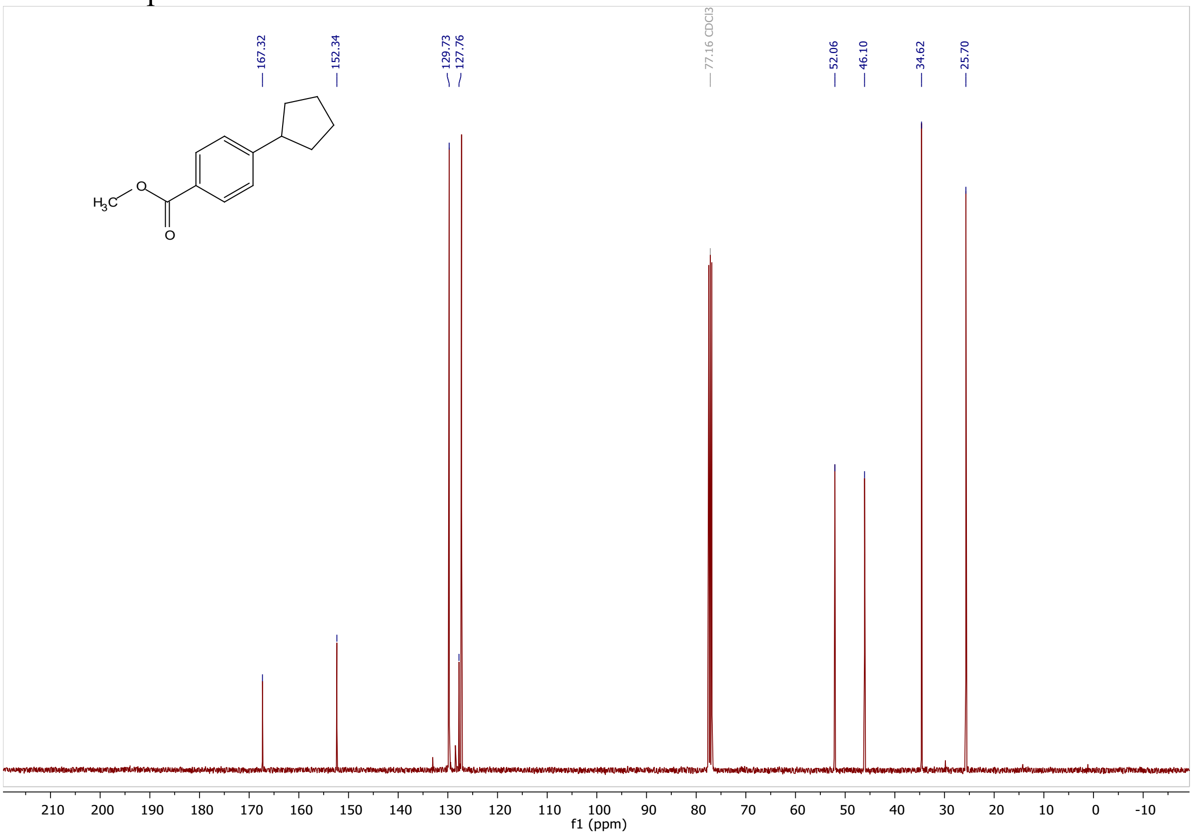

^{1}H NMR spectrum of **3b**

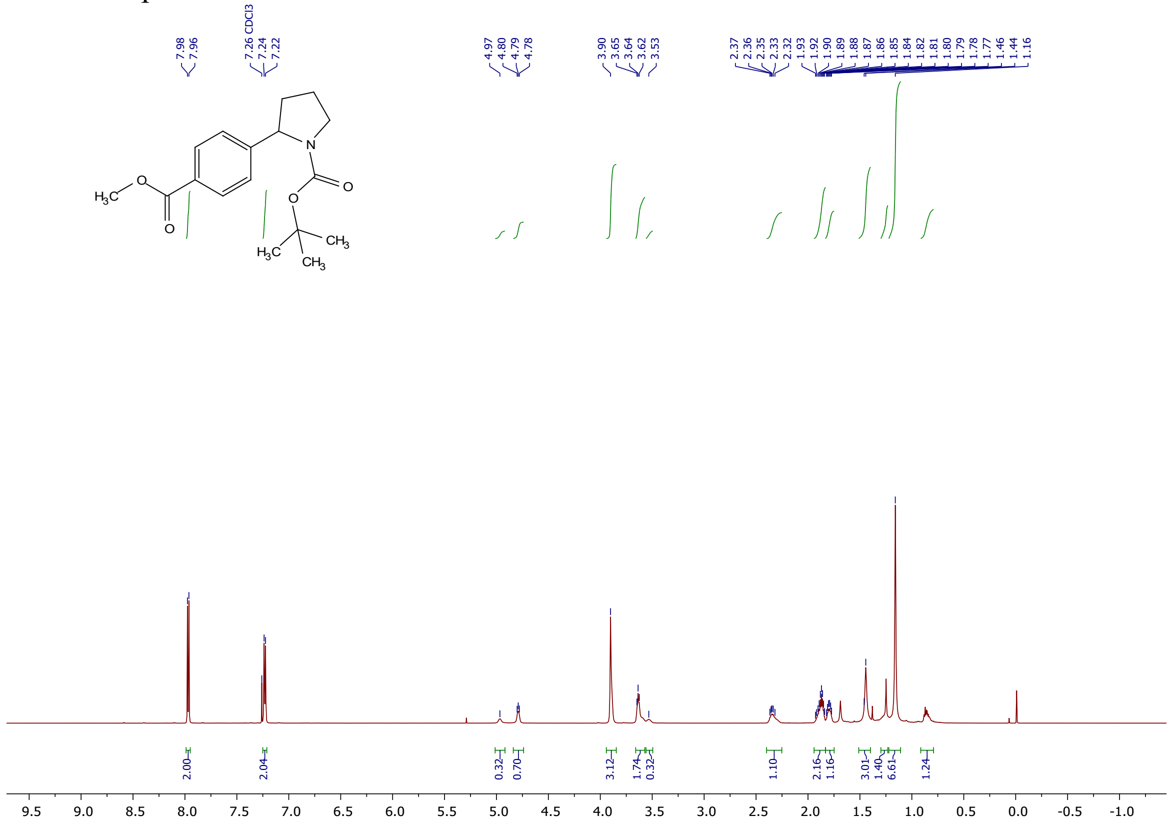

^{13}C NMR spectrum of **3b**

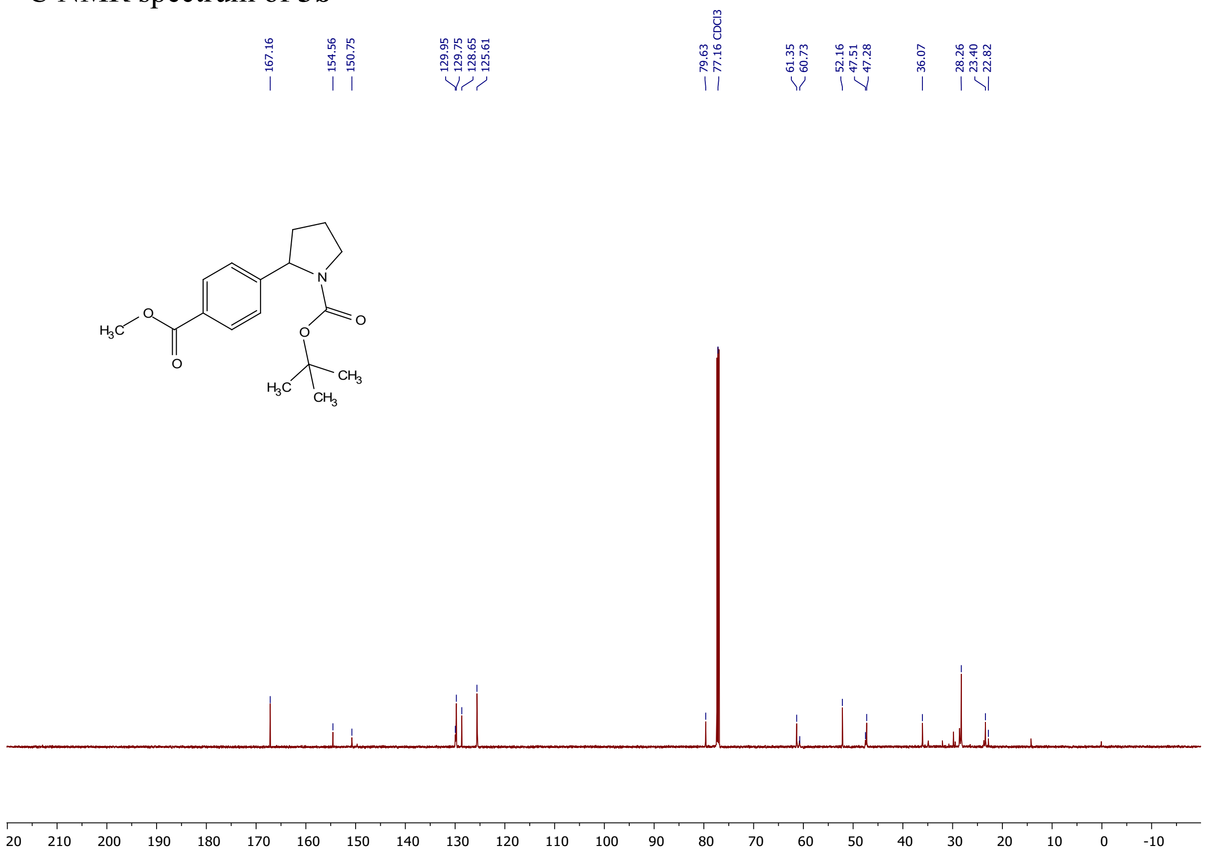

^{1}H NMR spectrum of **3c**

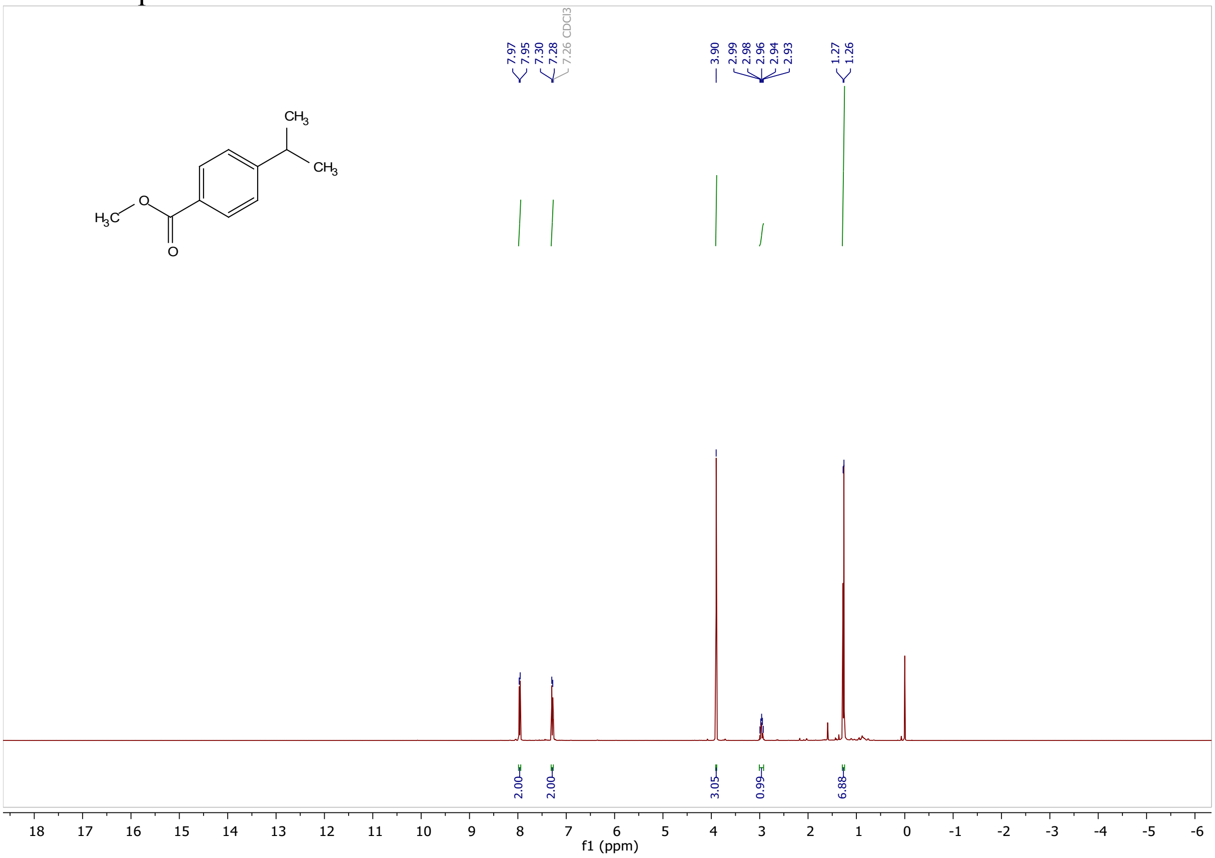

^{13}C NMR spectrum of **3c**

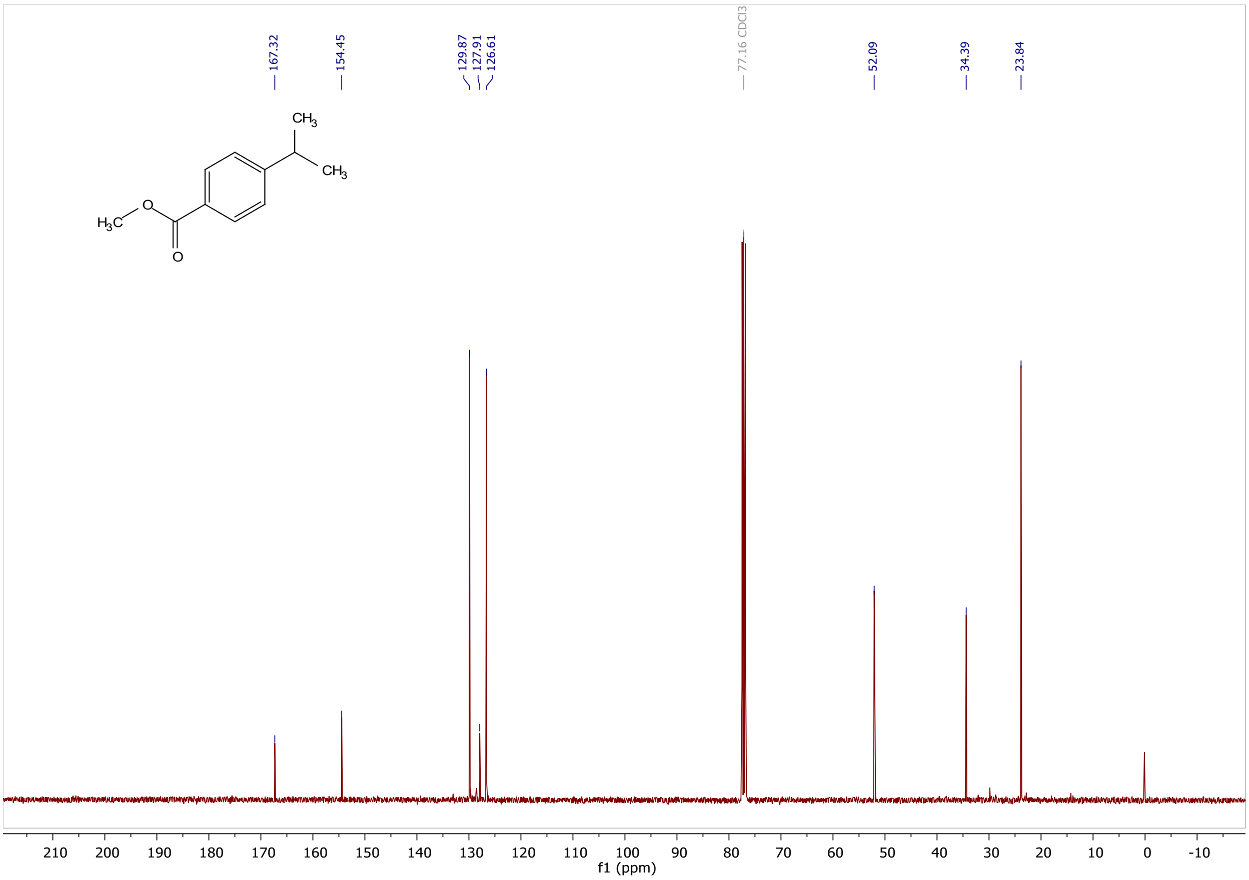

^{1}H NMR spectrum of **3d**

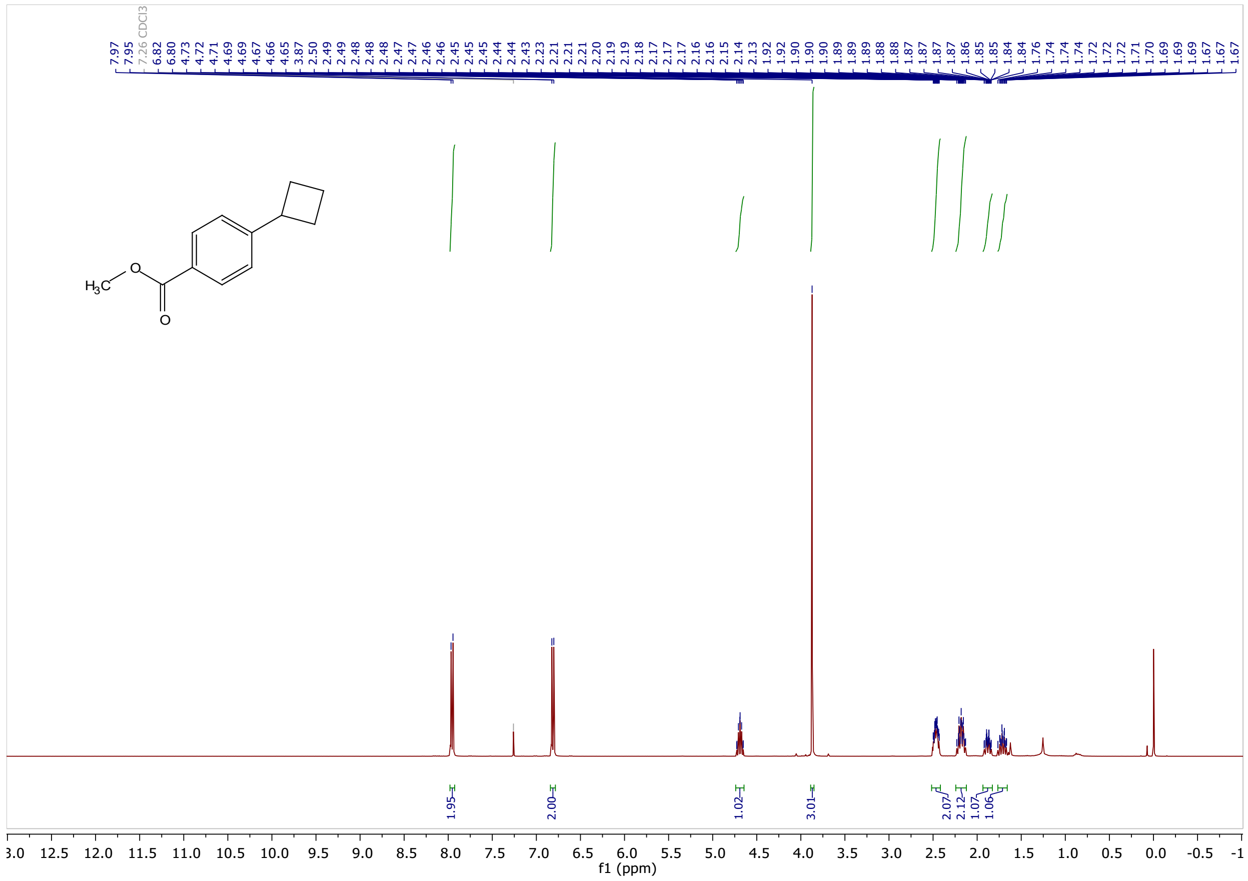

^{13}C NMR spectrum of **3d**

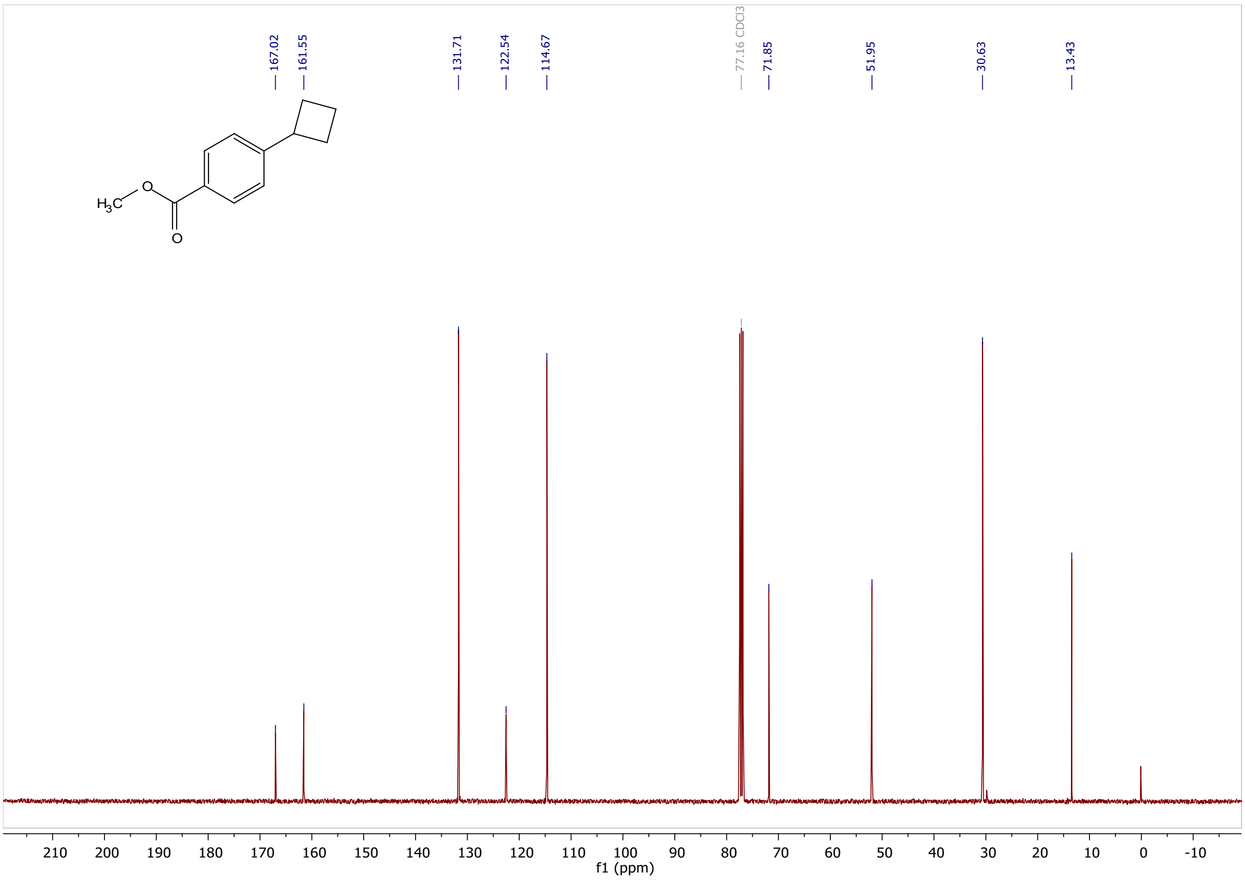

^{1}H NMR spectrum of **3e**

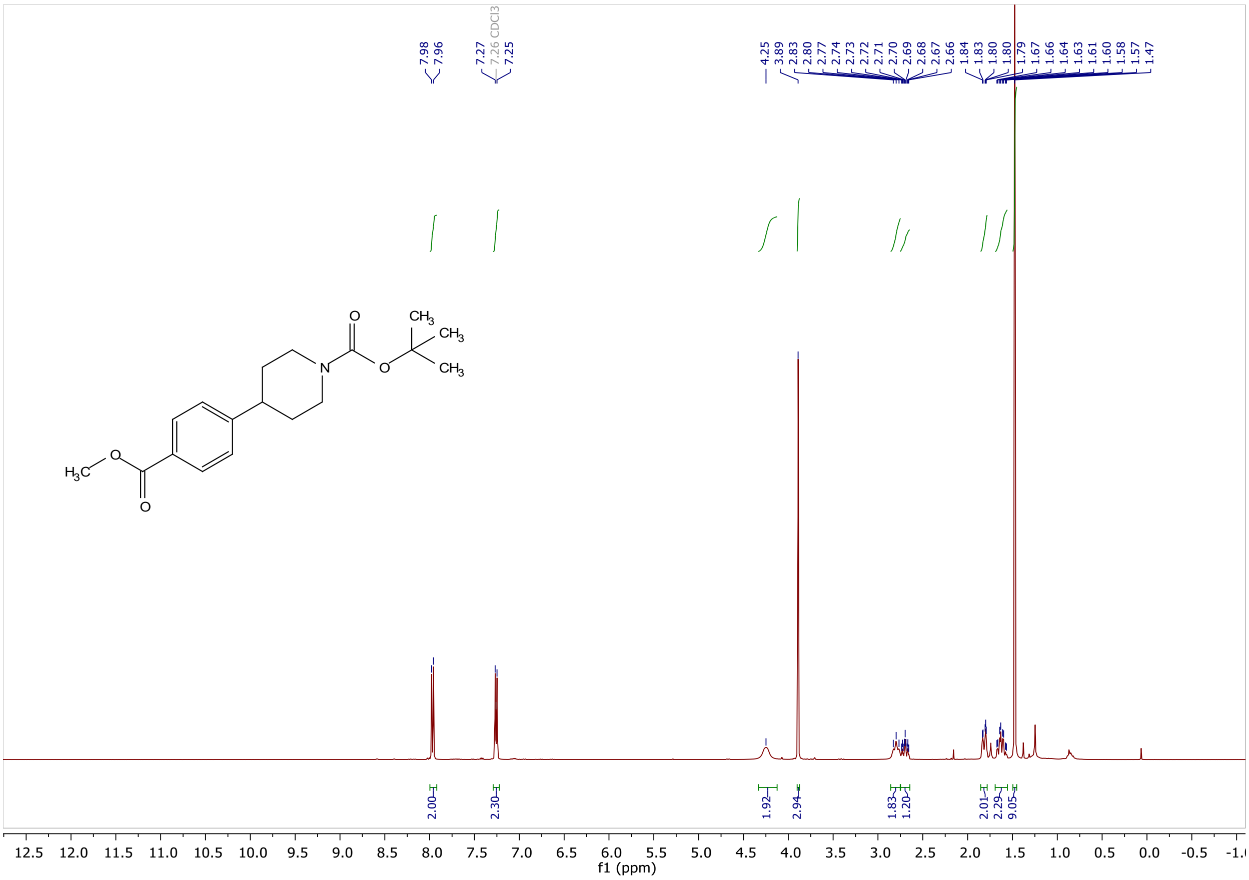

^{13}C NMR spectrum of **3e**

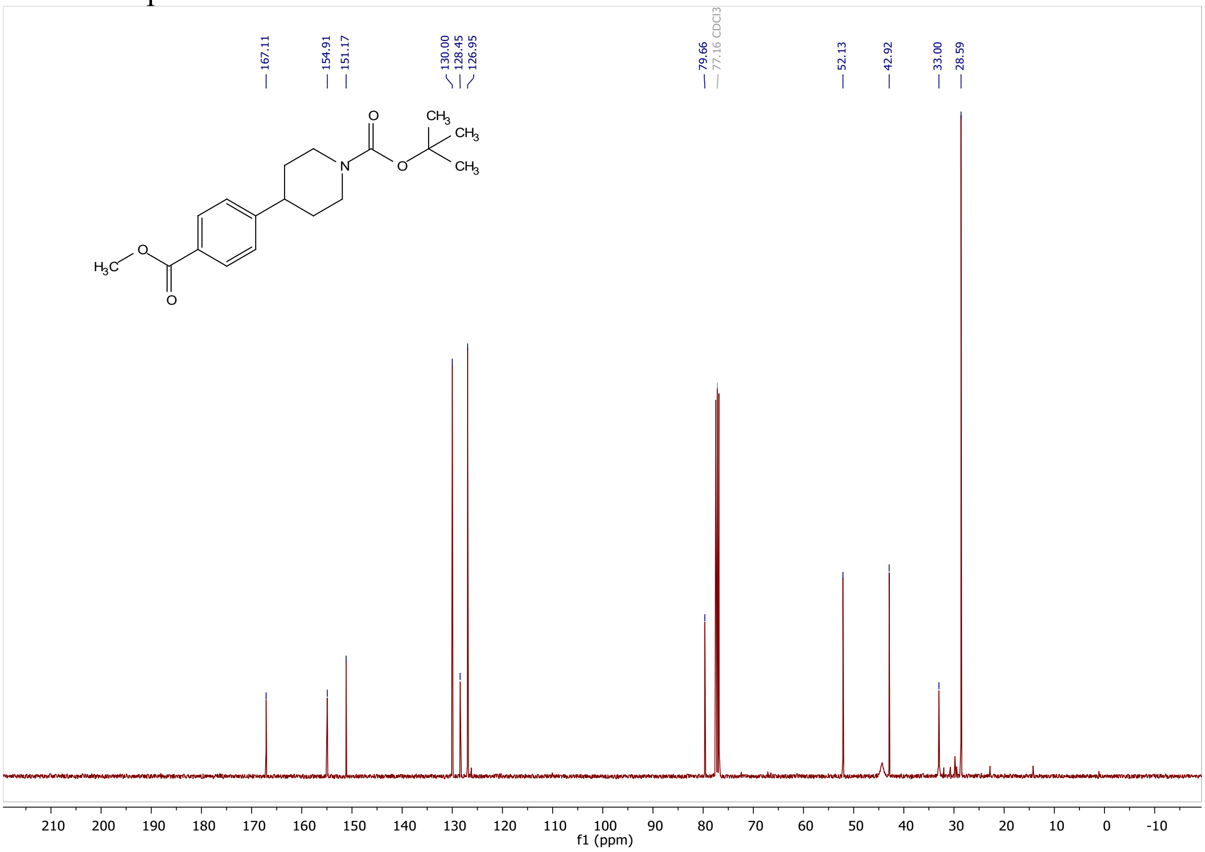

^{1}H NMR spectrum of **3f**

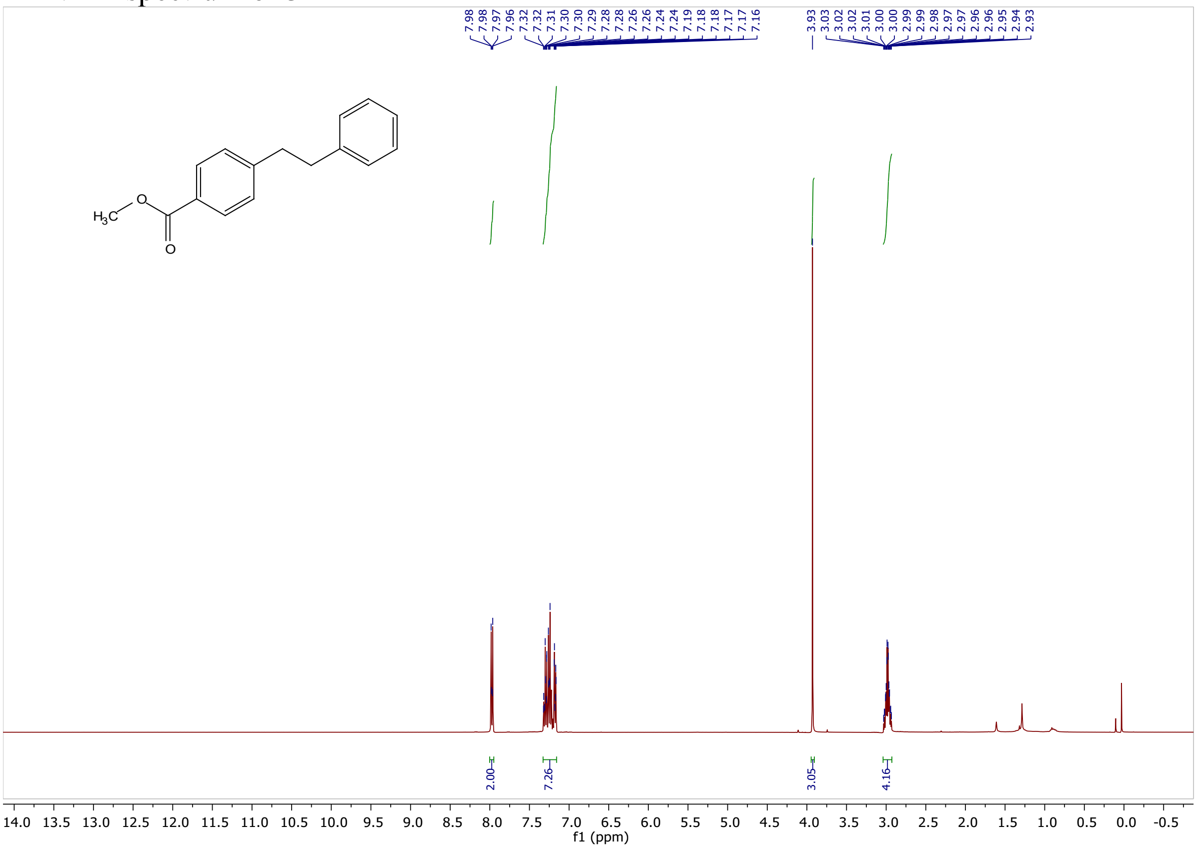

^{13}C NMR spectrum of **3f**

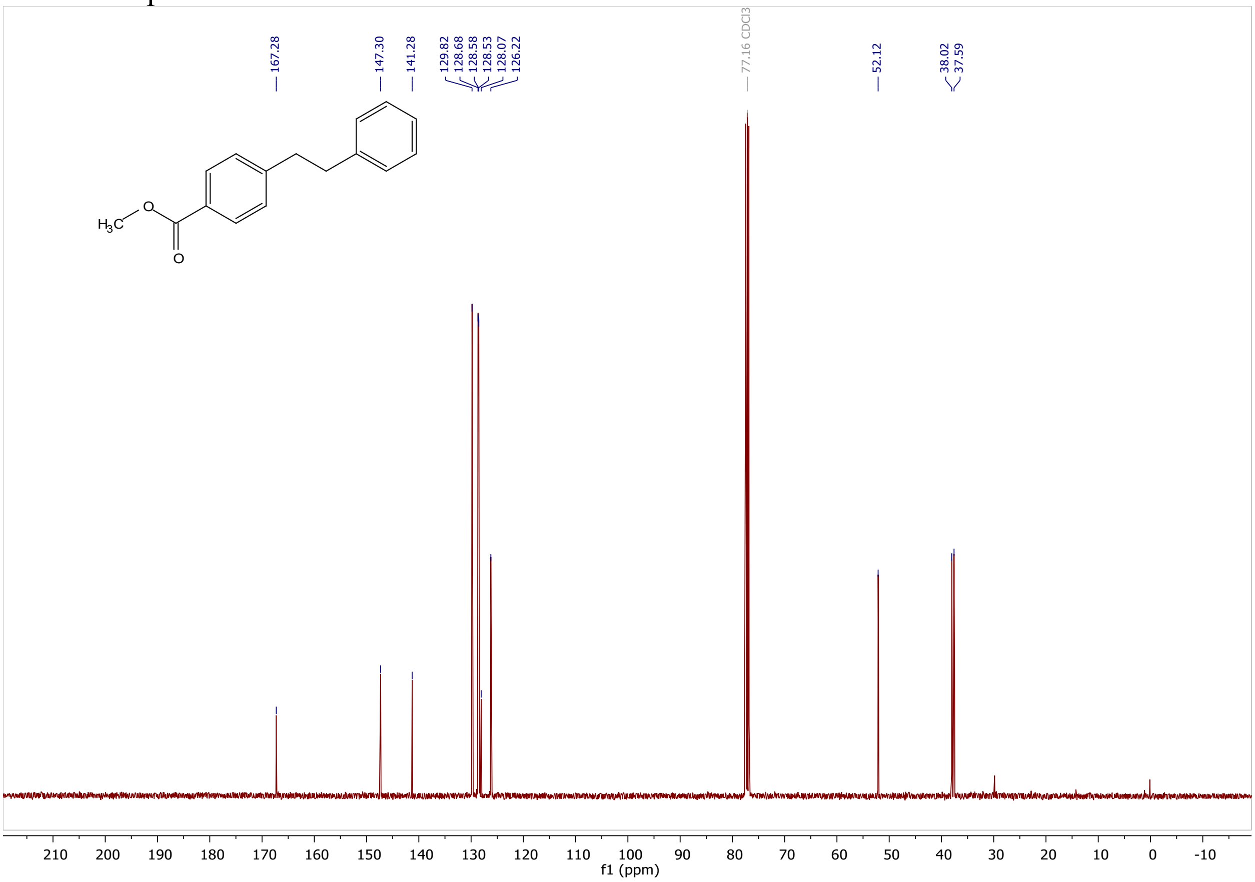

^{1}H NMR spectrum of **3g**

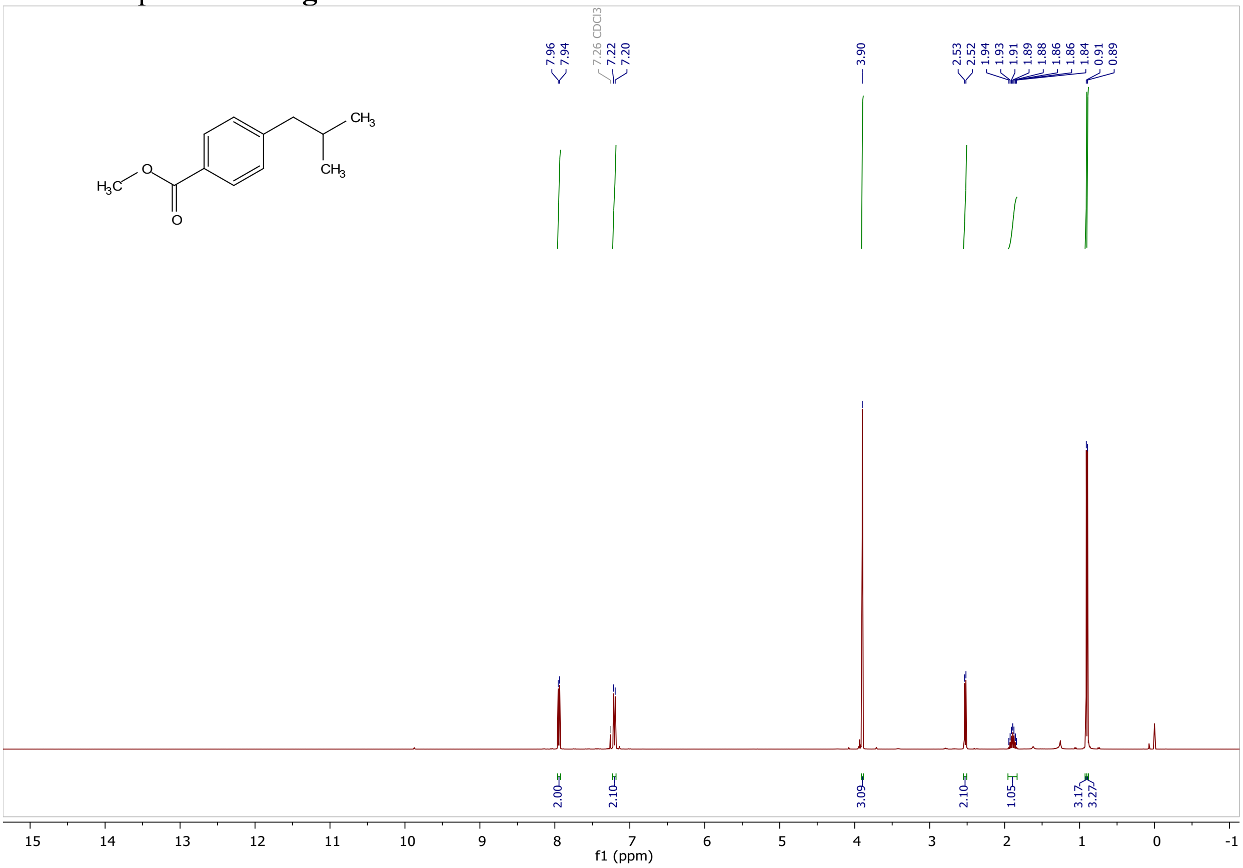

^{13}C NMR spectrum of **3g**

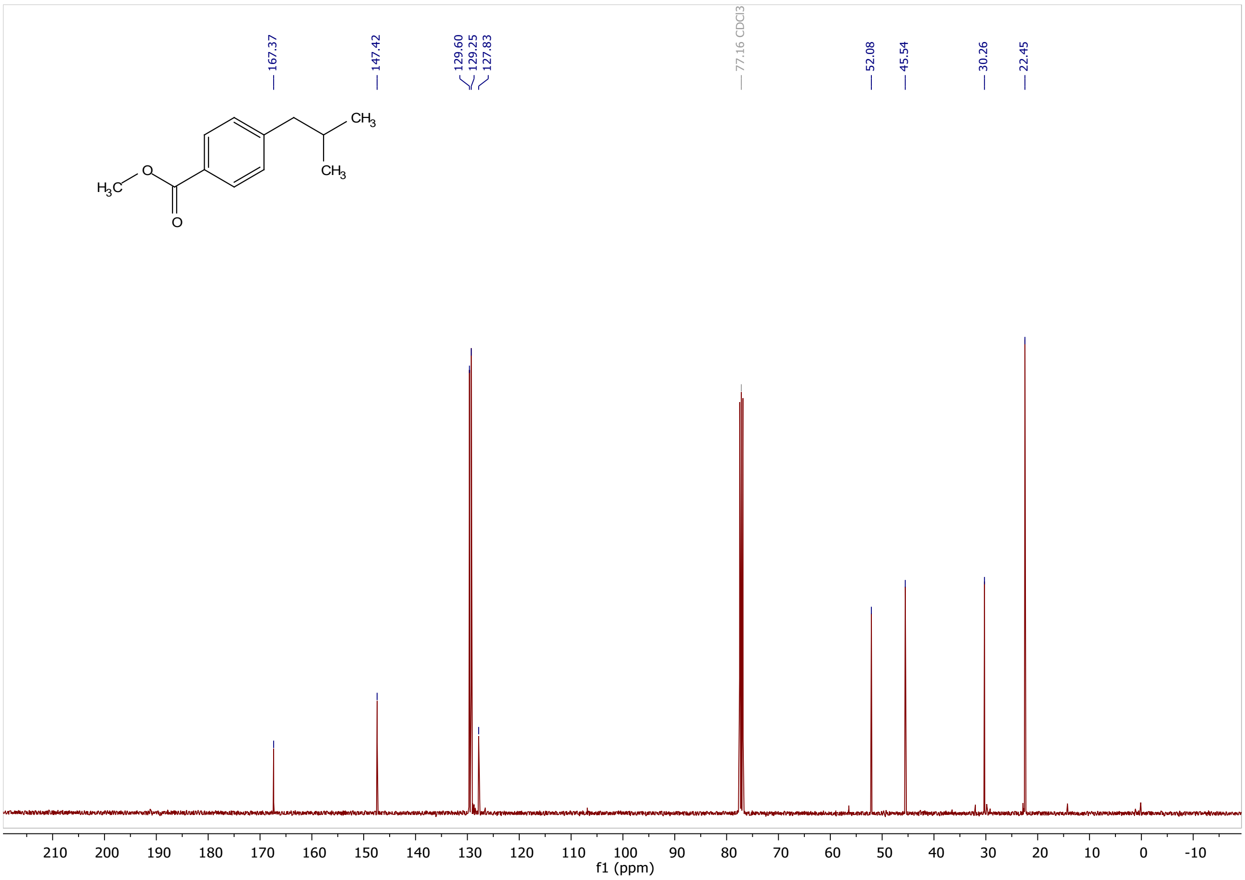

^{1}H NMR spectrum of **3h**

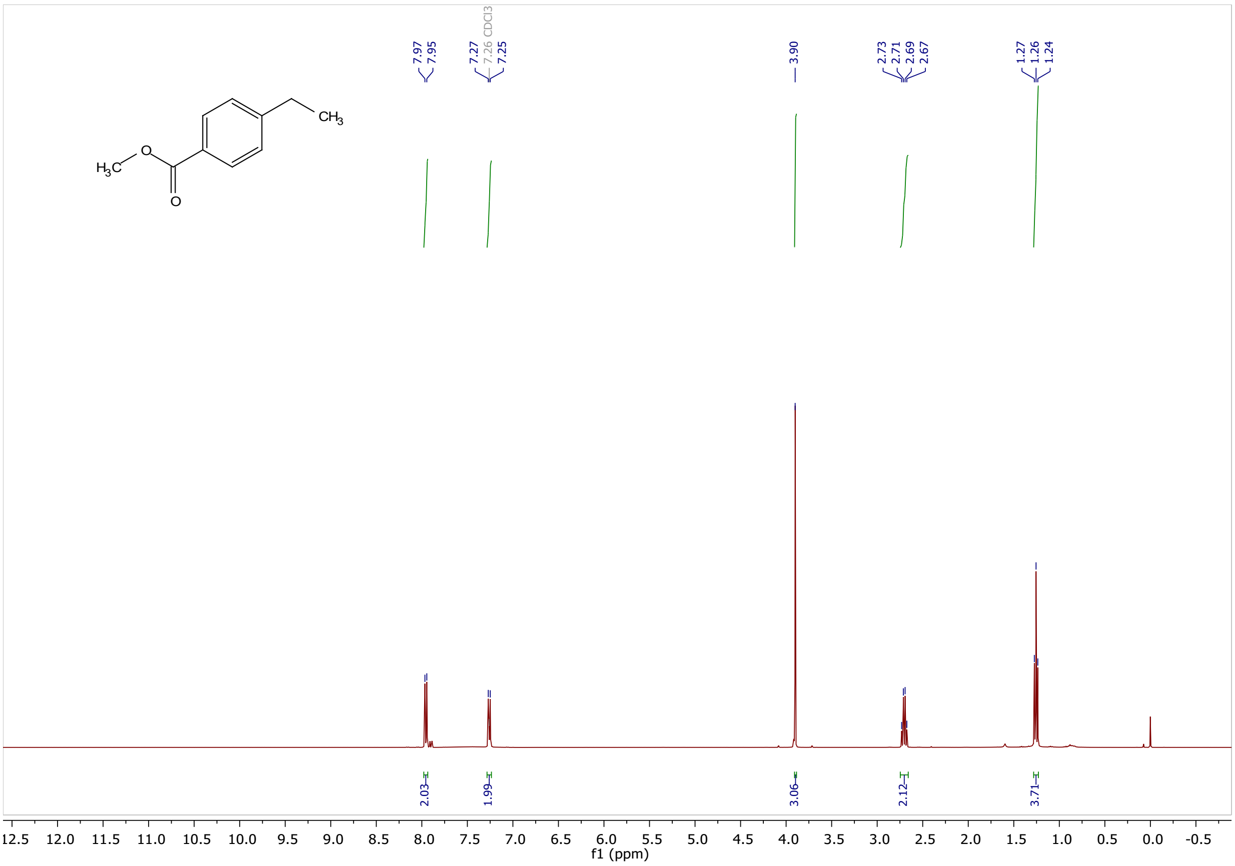

^{13}C NMR spectrum of **3h**

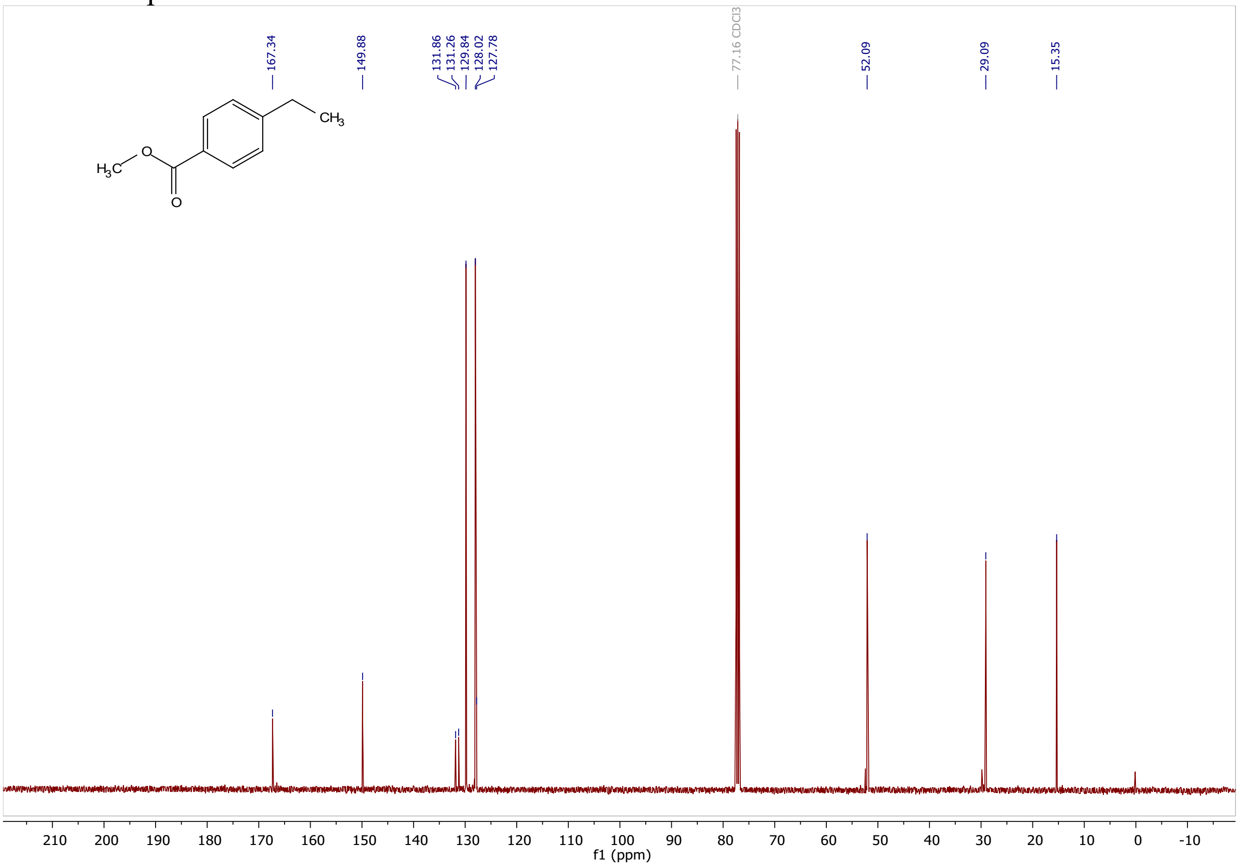

^{1}H NMR spectrum of **3i**

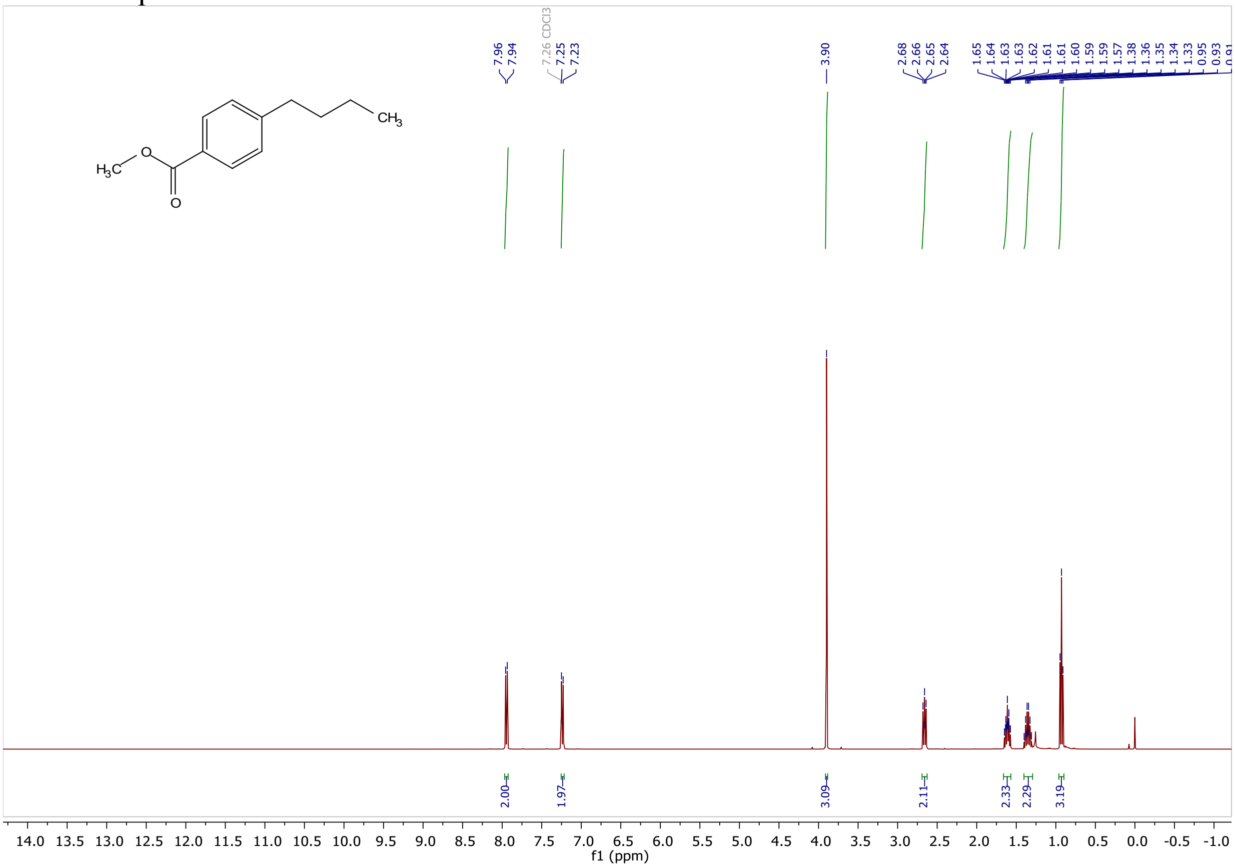

^{13}C NMR spectrum of **3i**

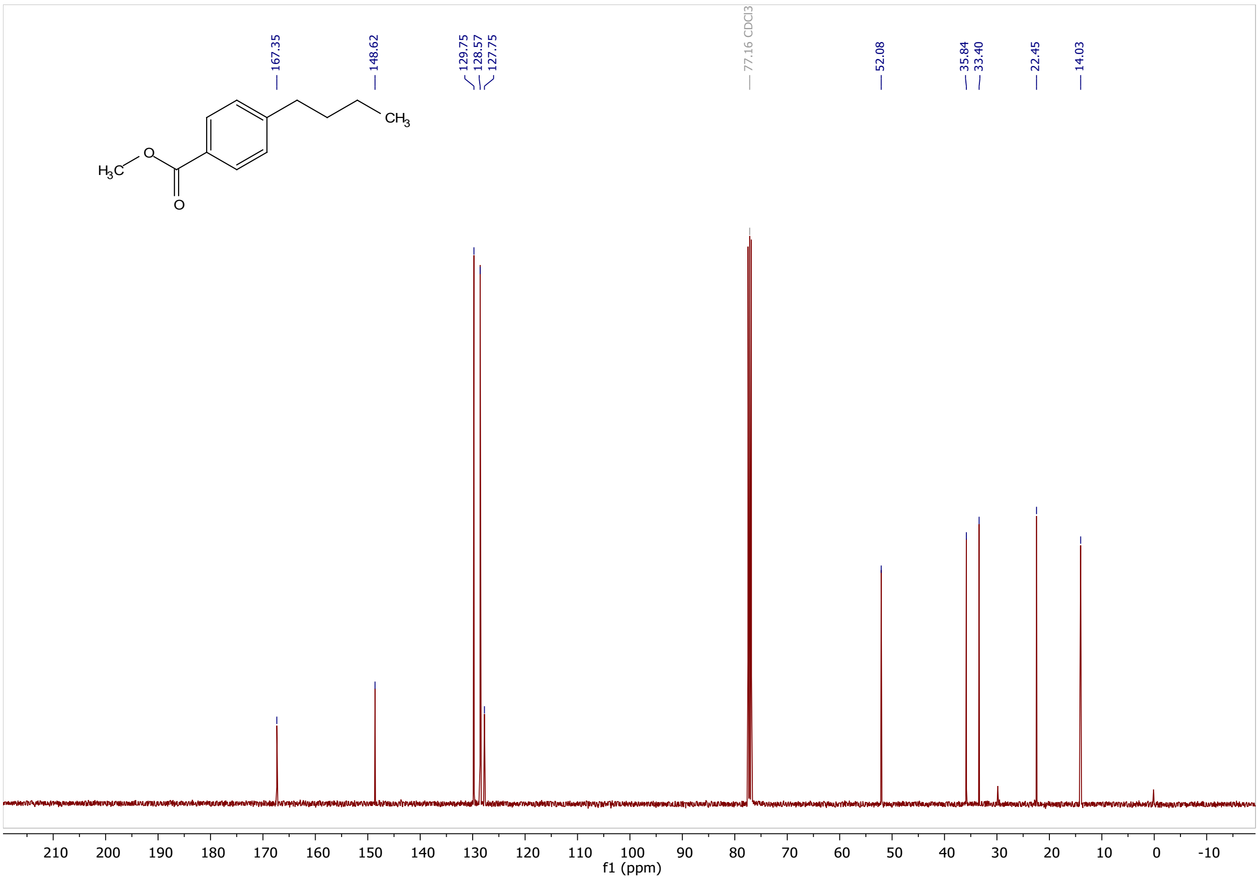

^{1}H NMR spectrum of **3j**

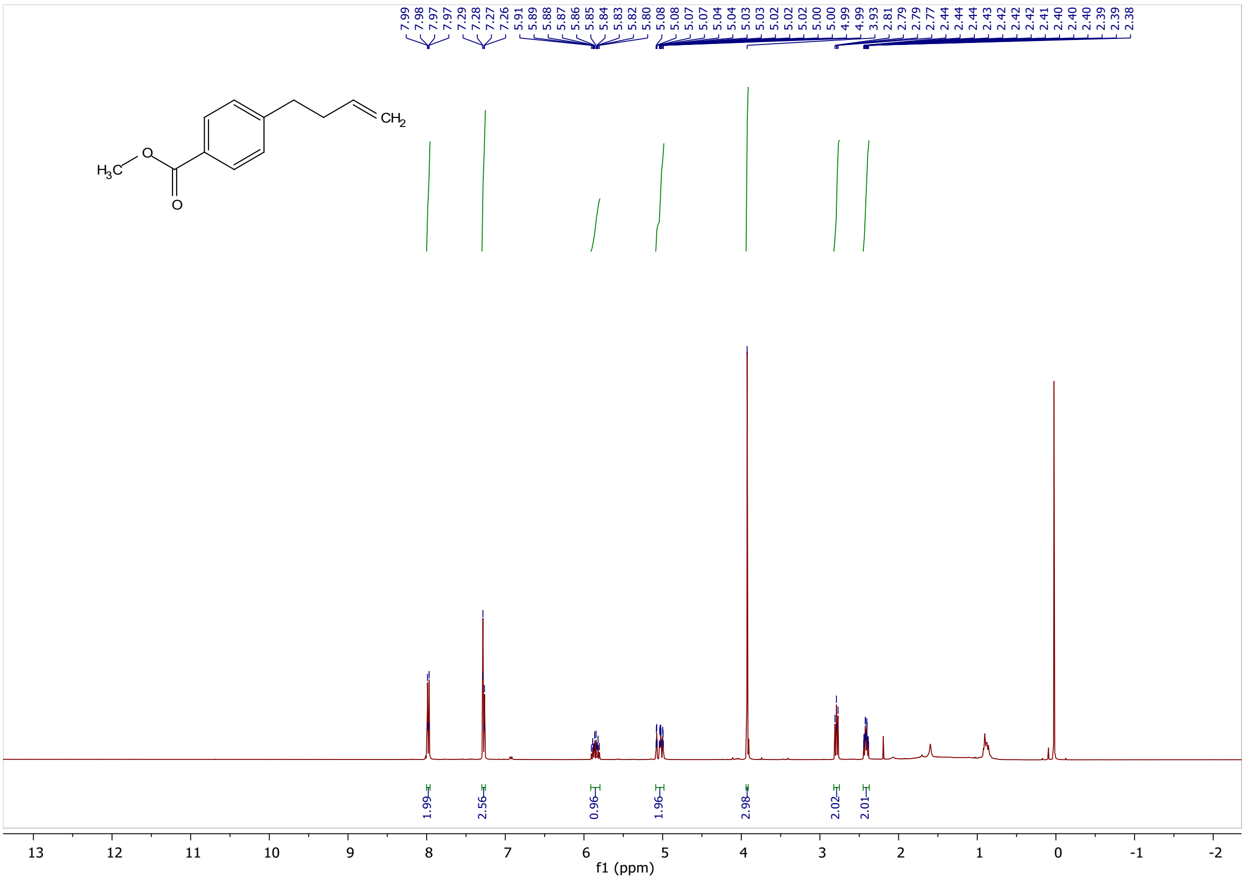

^{13}C NMR spectrum of **3j**

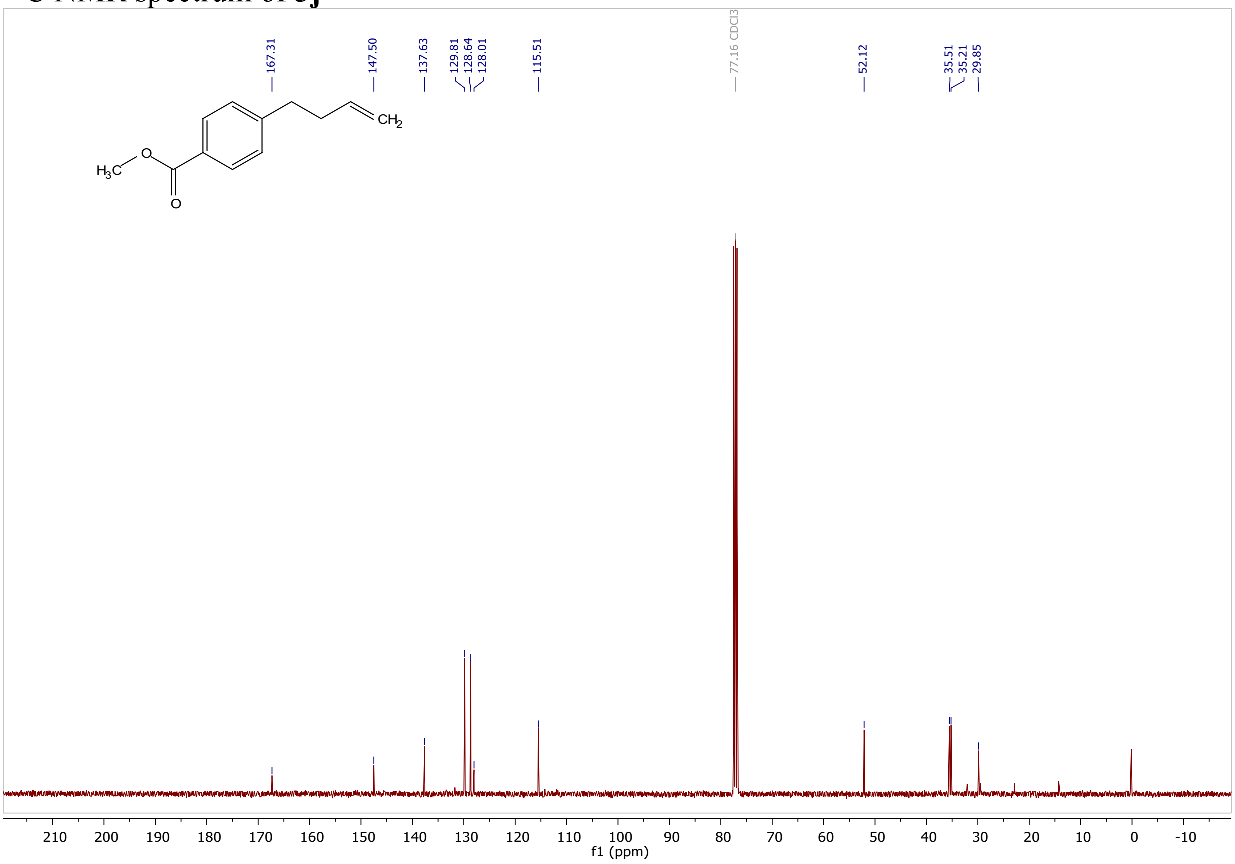

^{1}H NMR spectrum of **4a**

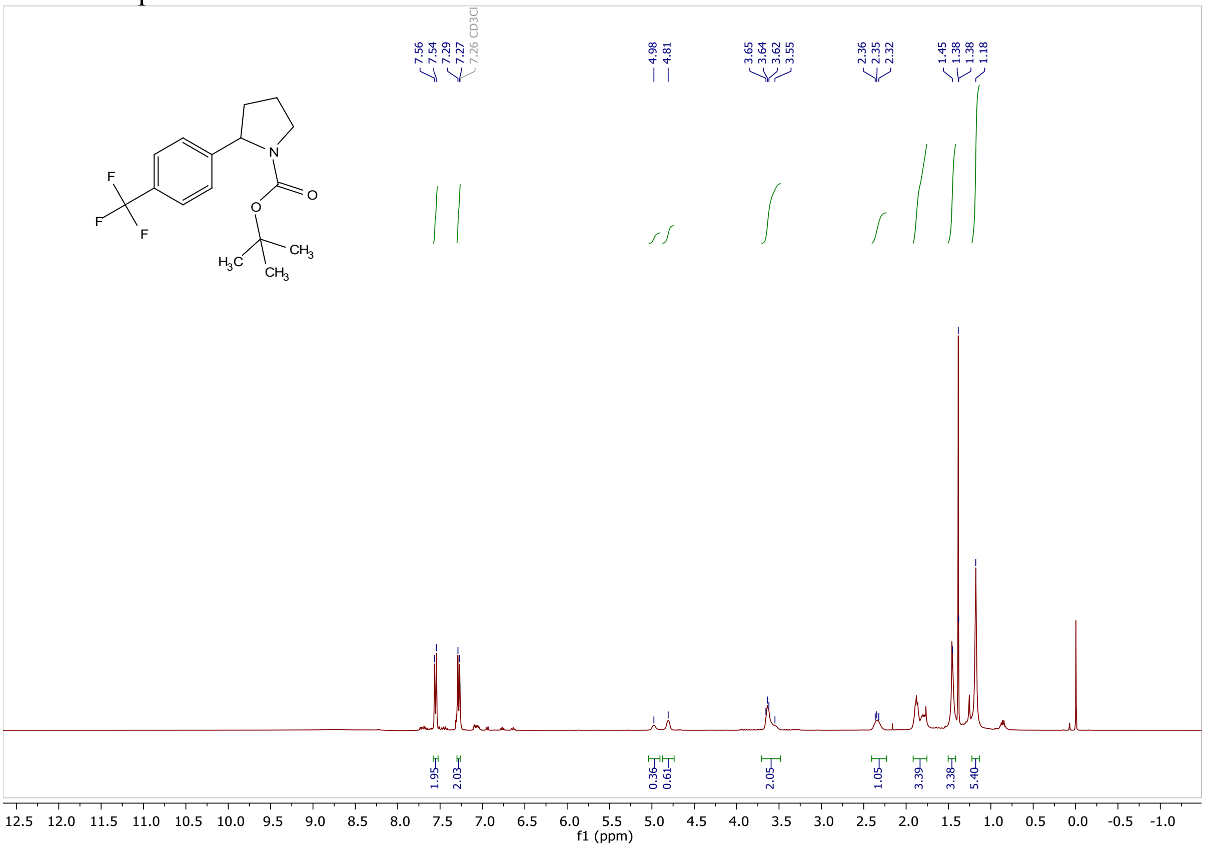

^{13}C NMR spectrum of **4a**

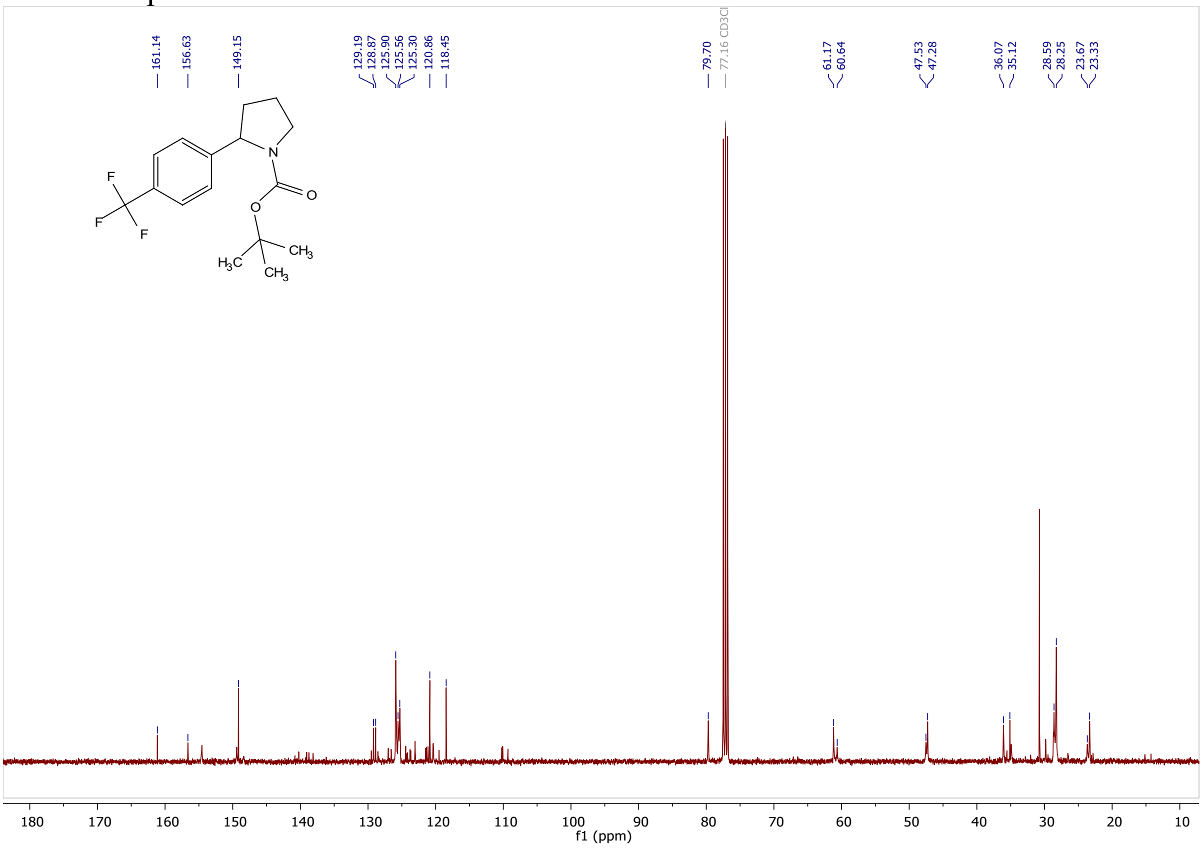

^{1}H NMR spectrum of **4b**

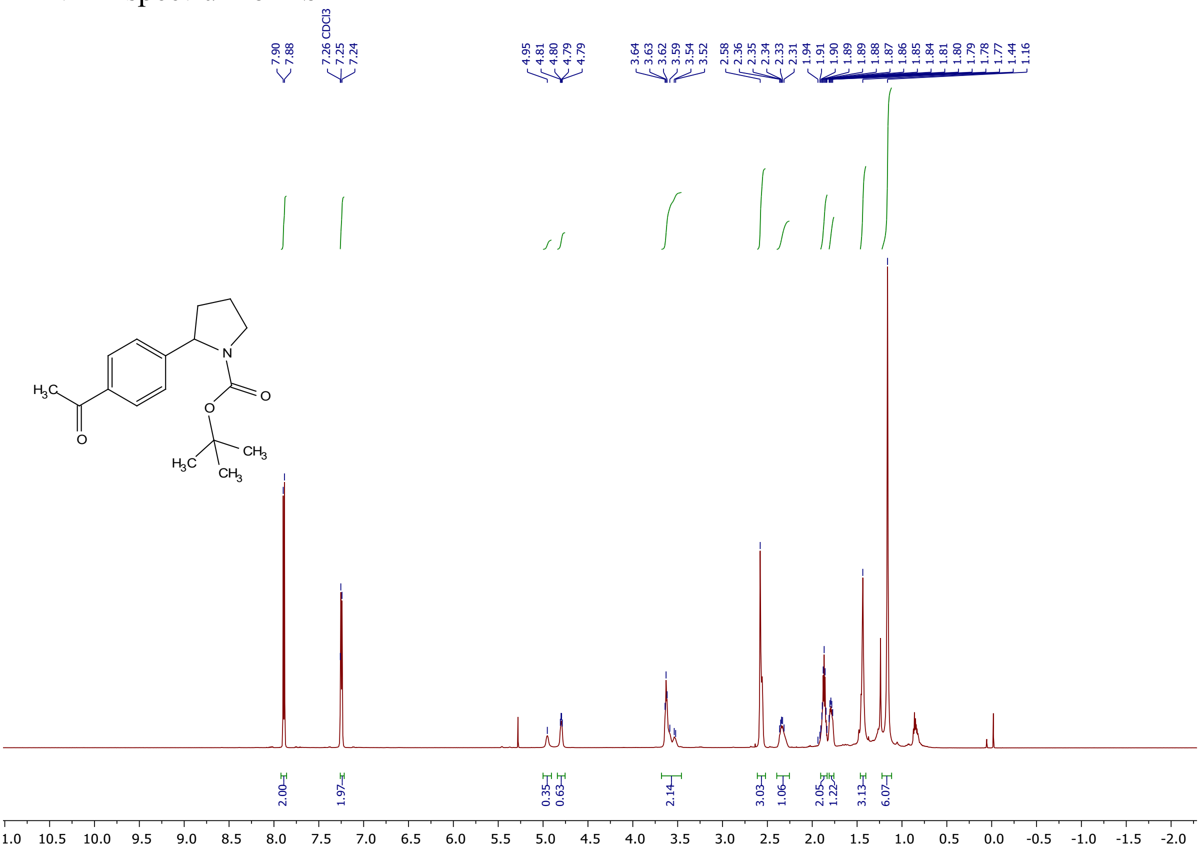

^{13}C NMR spectrum of **4b**

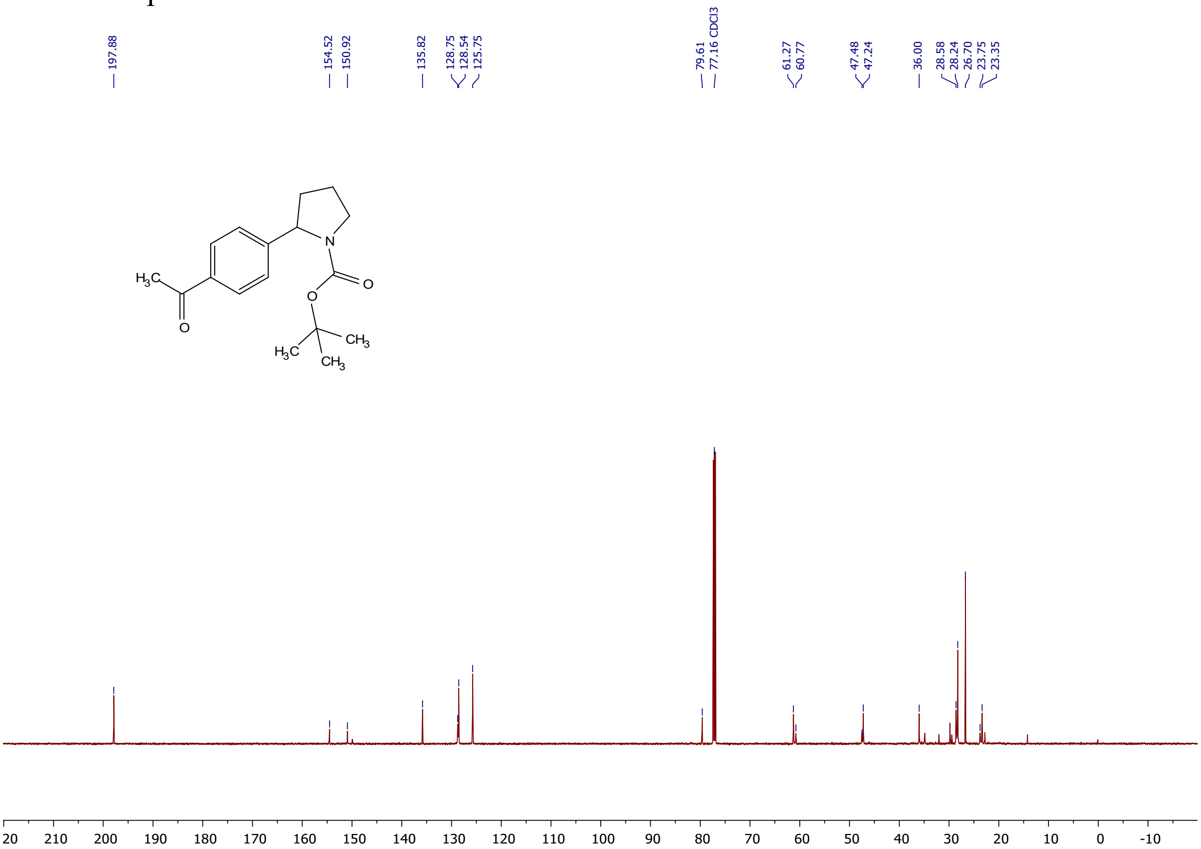

^{1}H NMR spectrum of **4c**

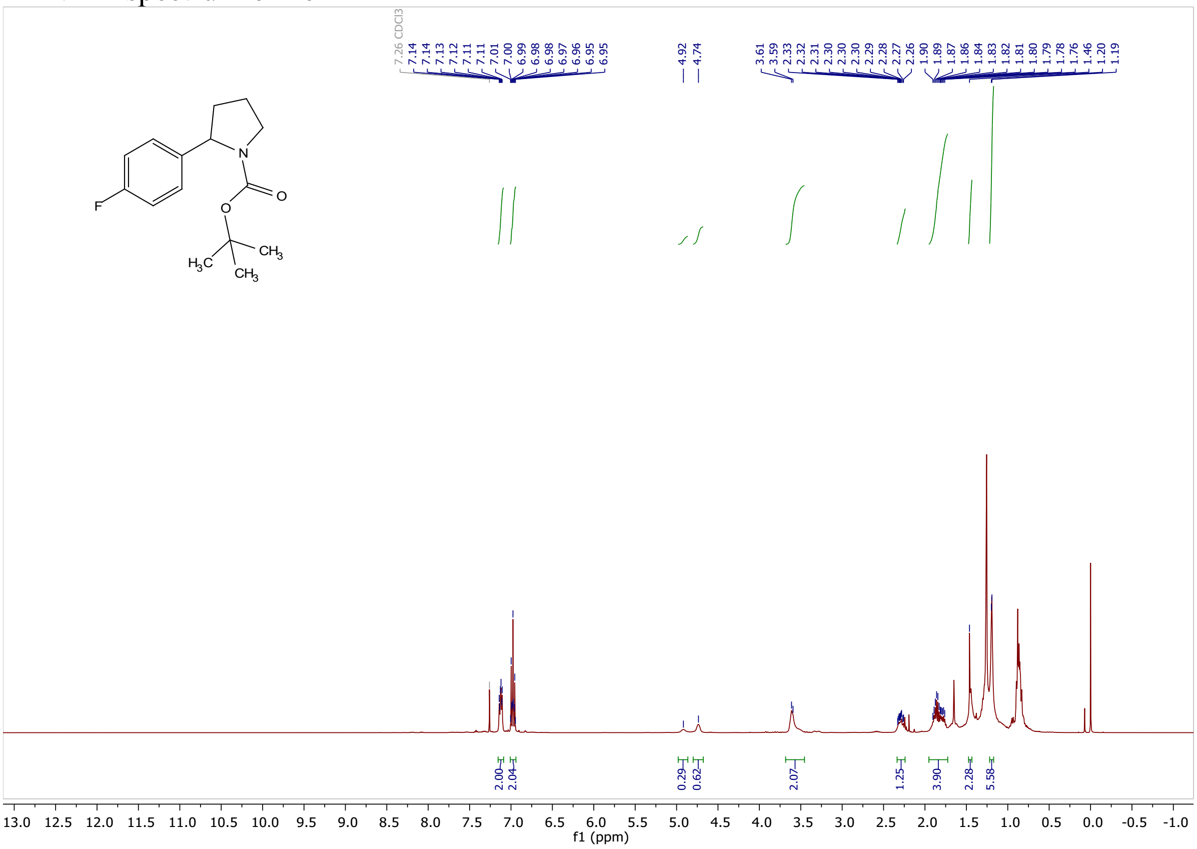

^{19}F NMR spectrum of **4c**

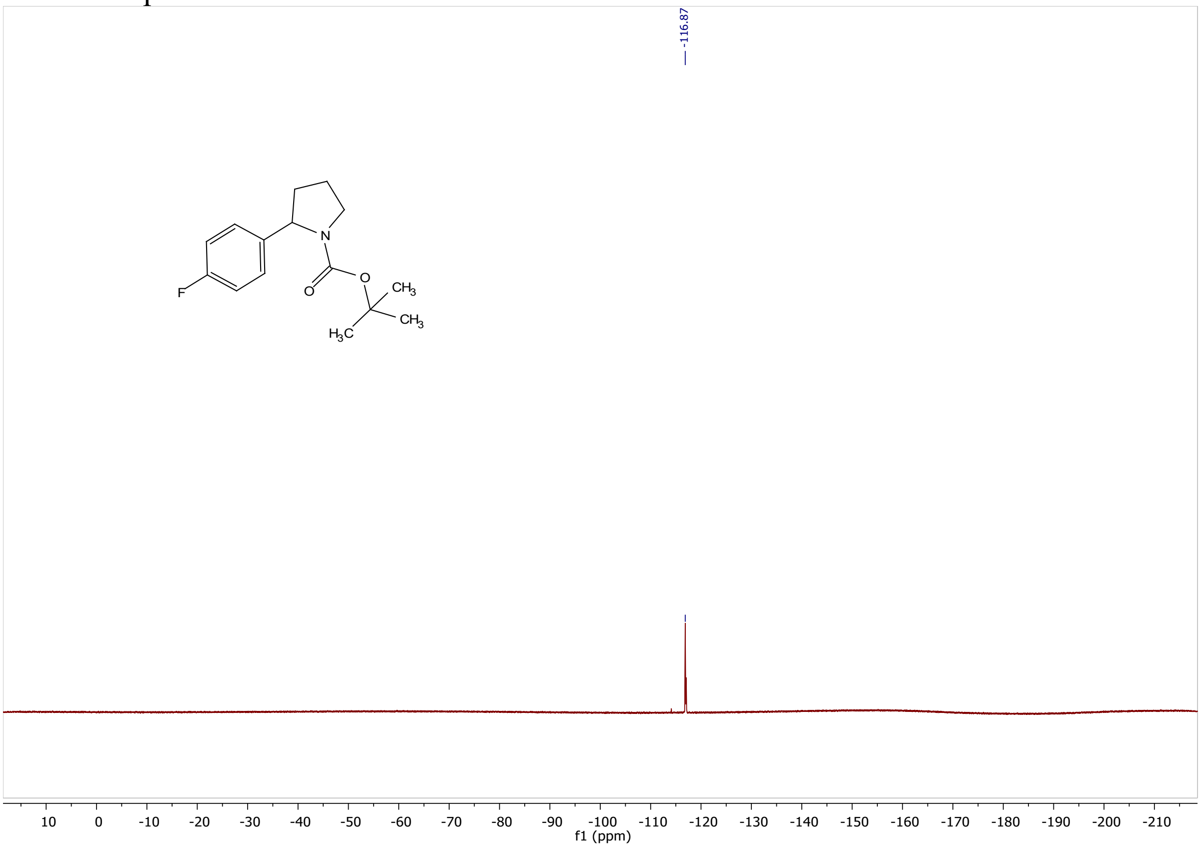

^{1}H NMR spectrum of **4d**

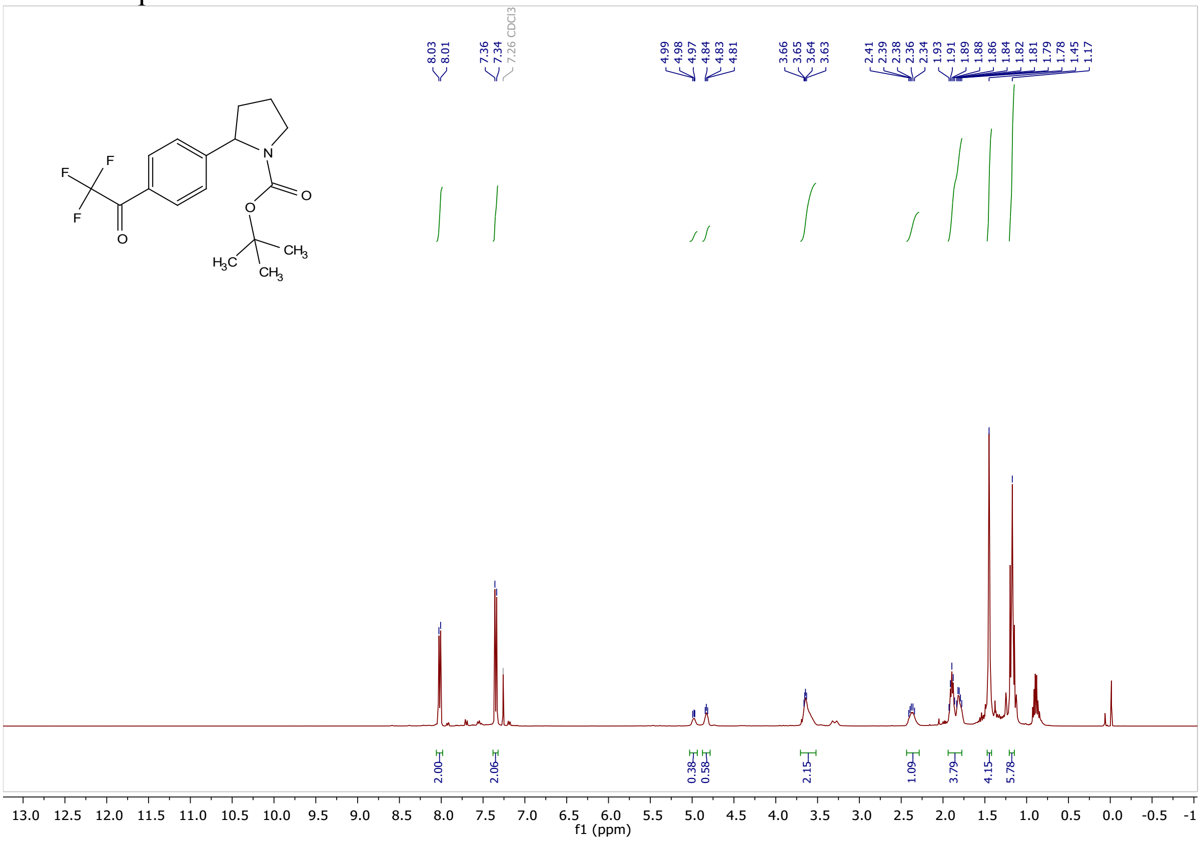

^{13}C NMR spectrum of **4d**

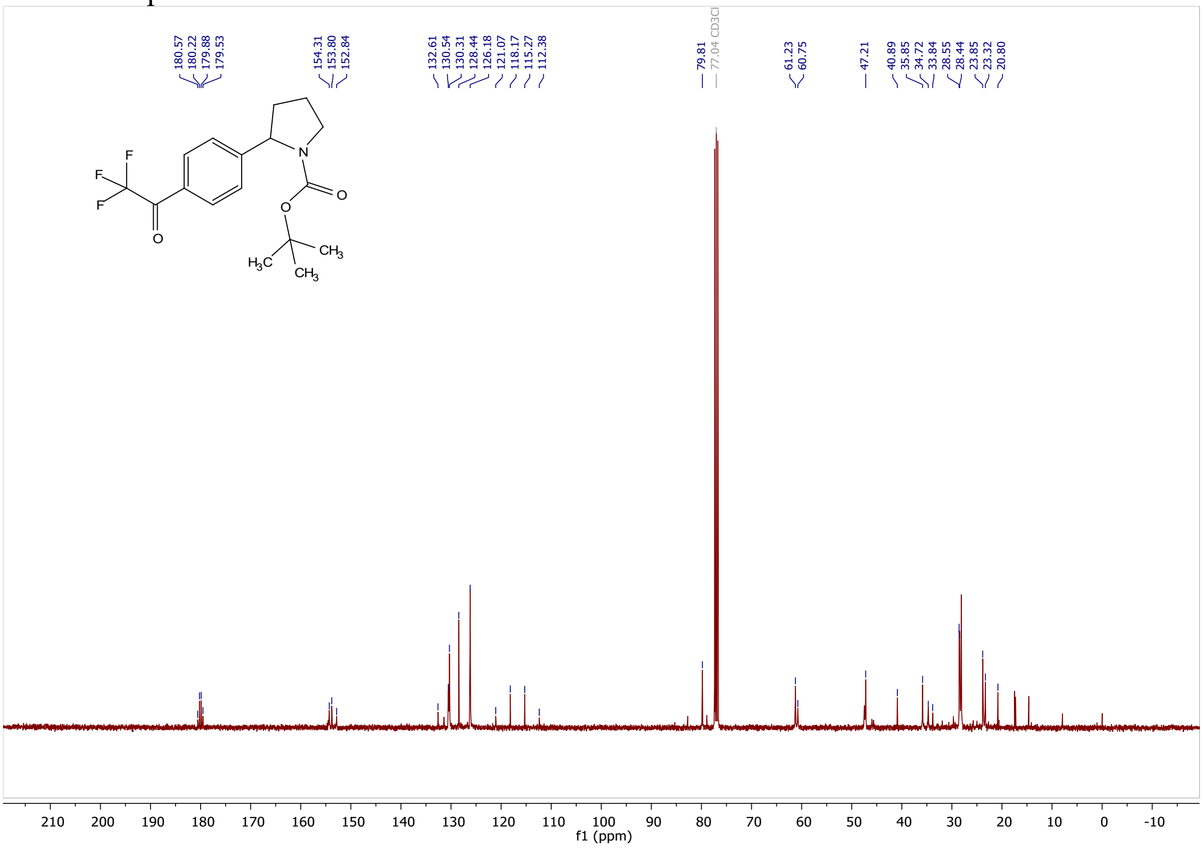

^{19}F NMR spectrum of **4d**

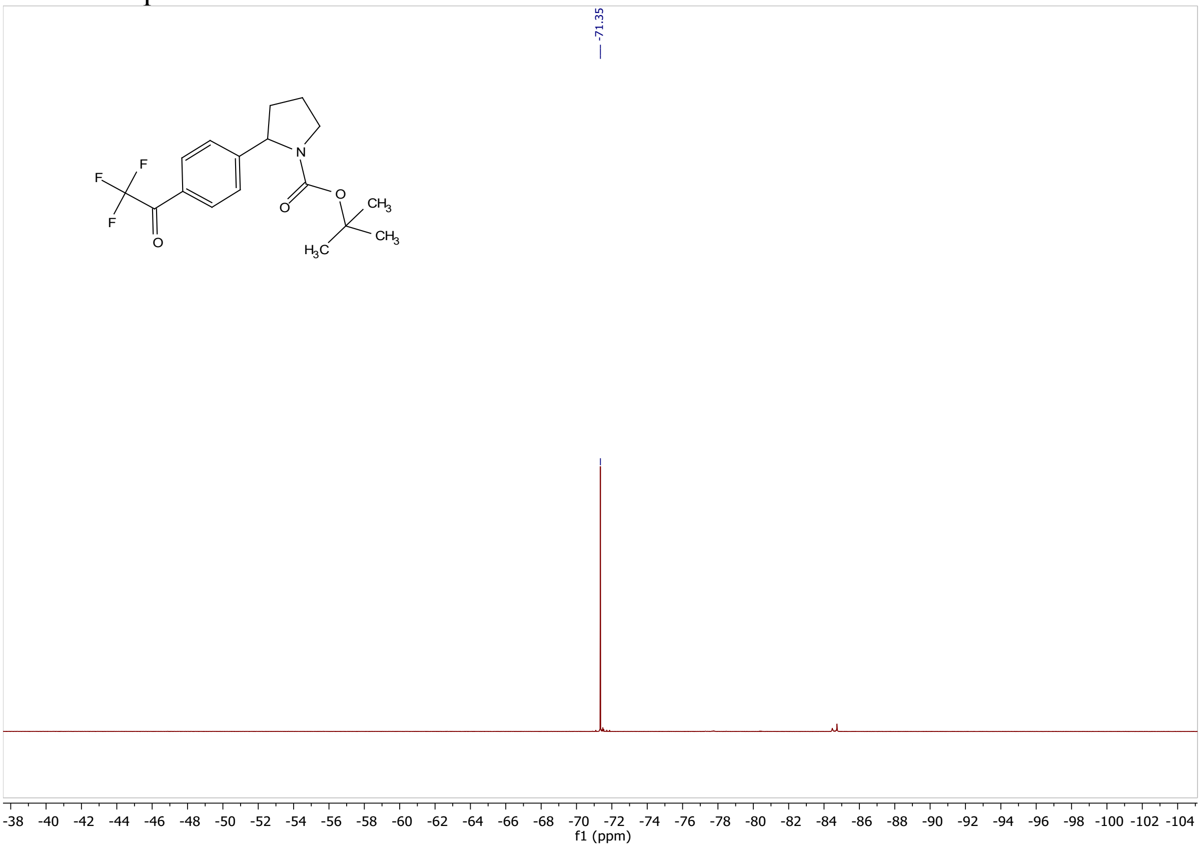

^{1}H NMR spectrum of **4e**

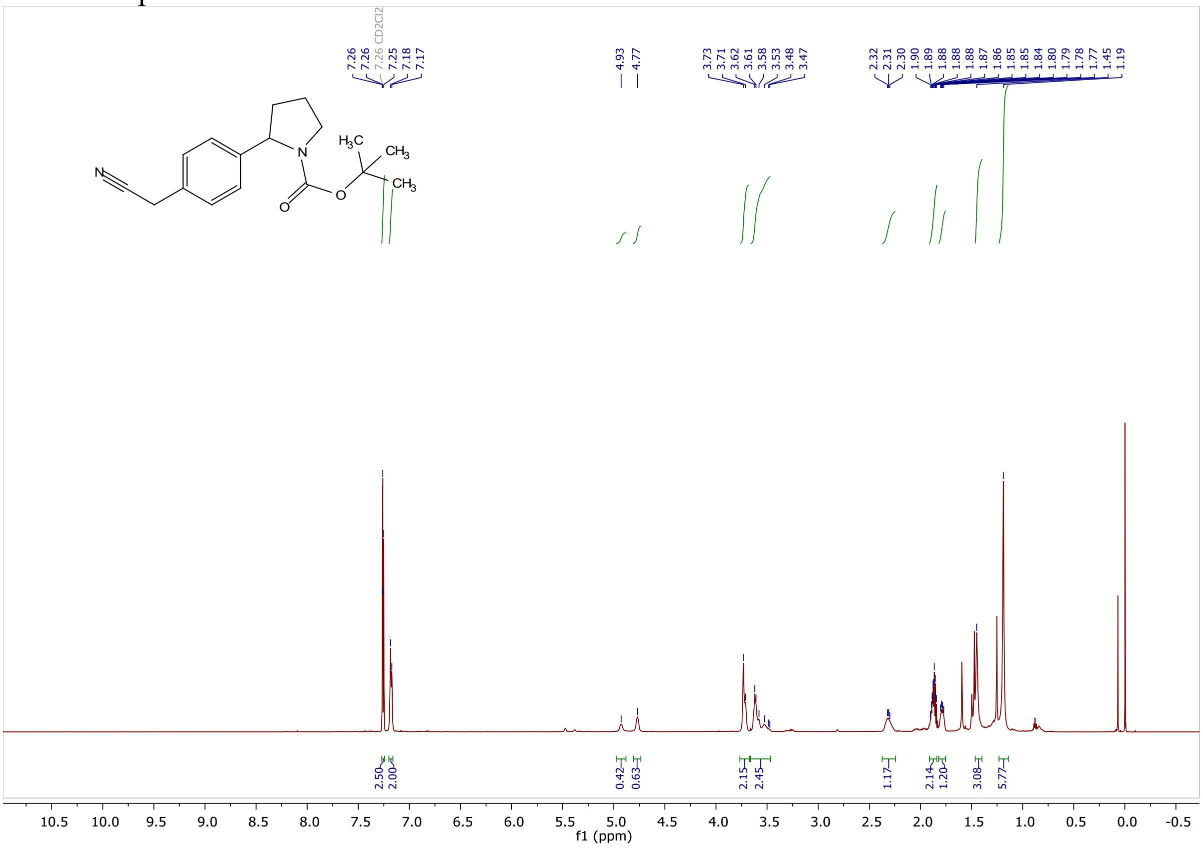

^{13}C NMR spectrum of **4e**

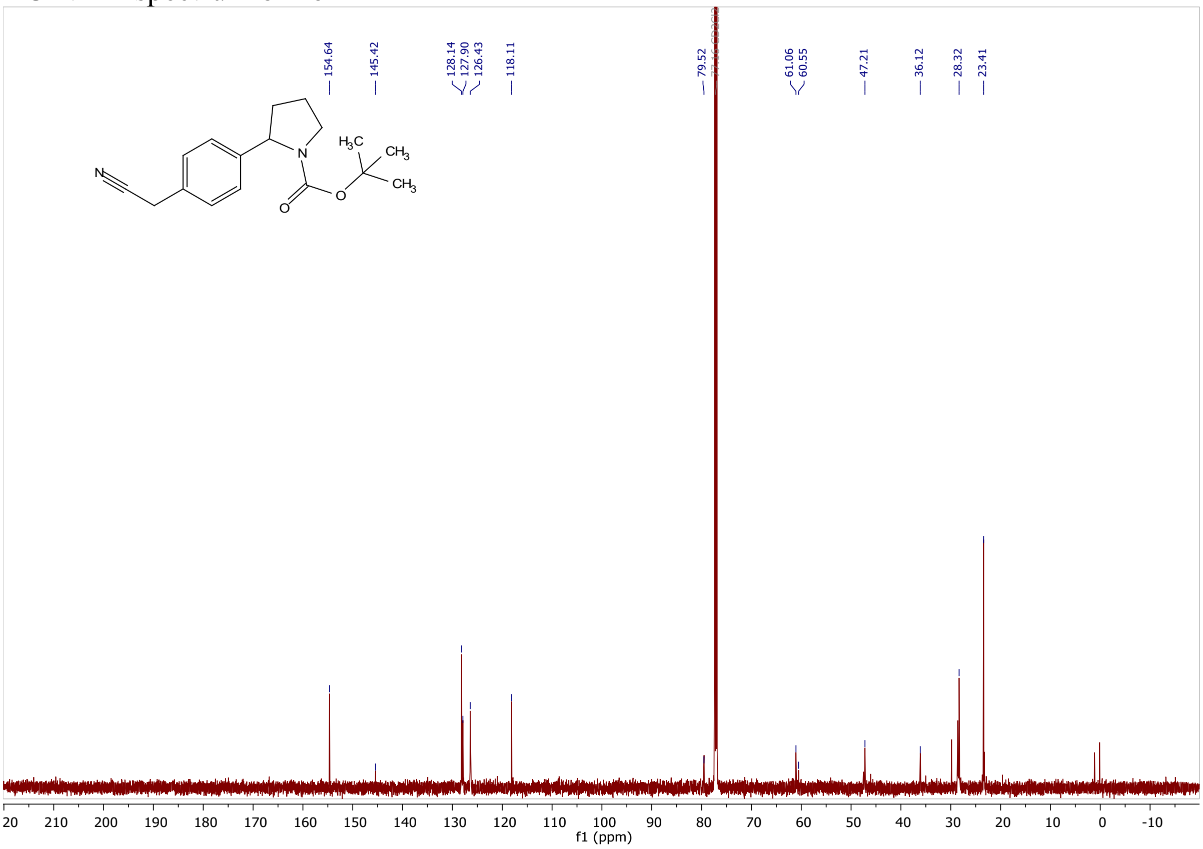

^{1}H NMR spectrum of **4g**

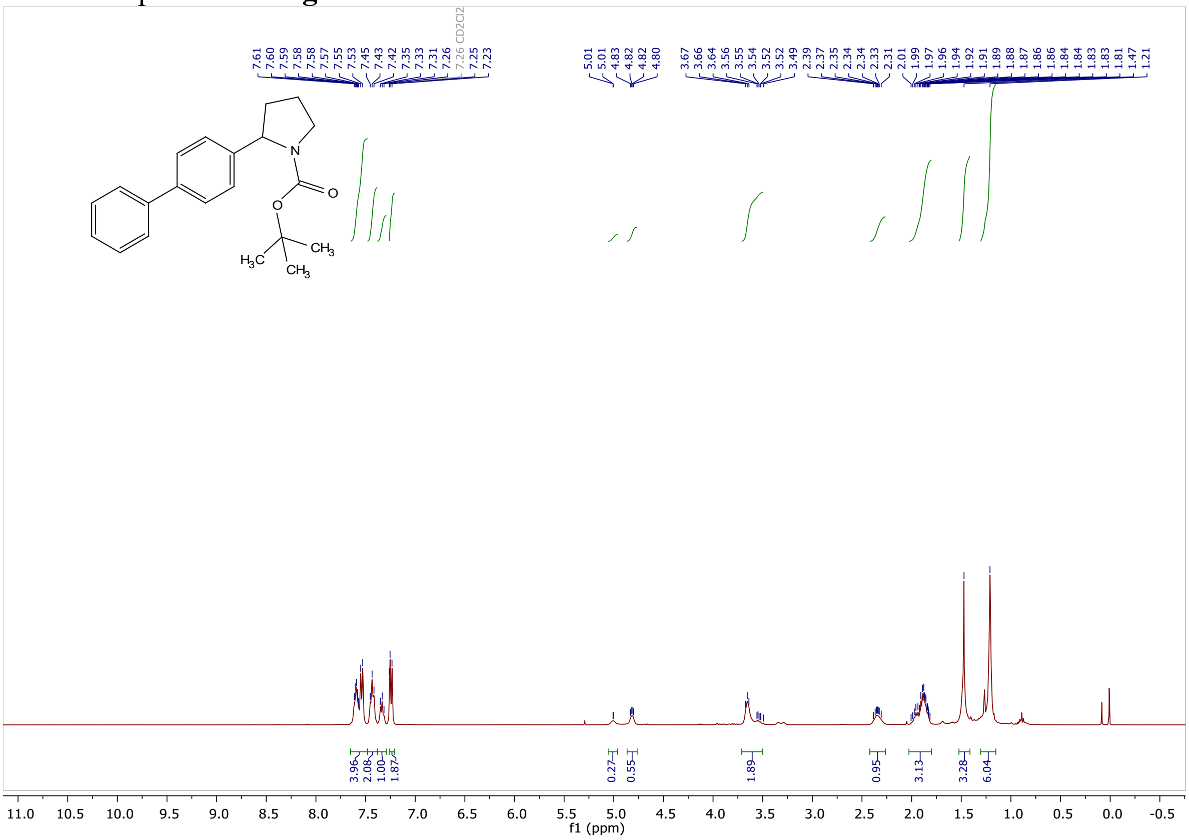

^{13}C NMR spectrum of **4g**

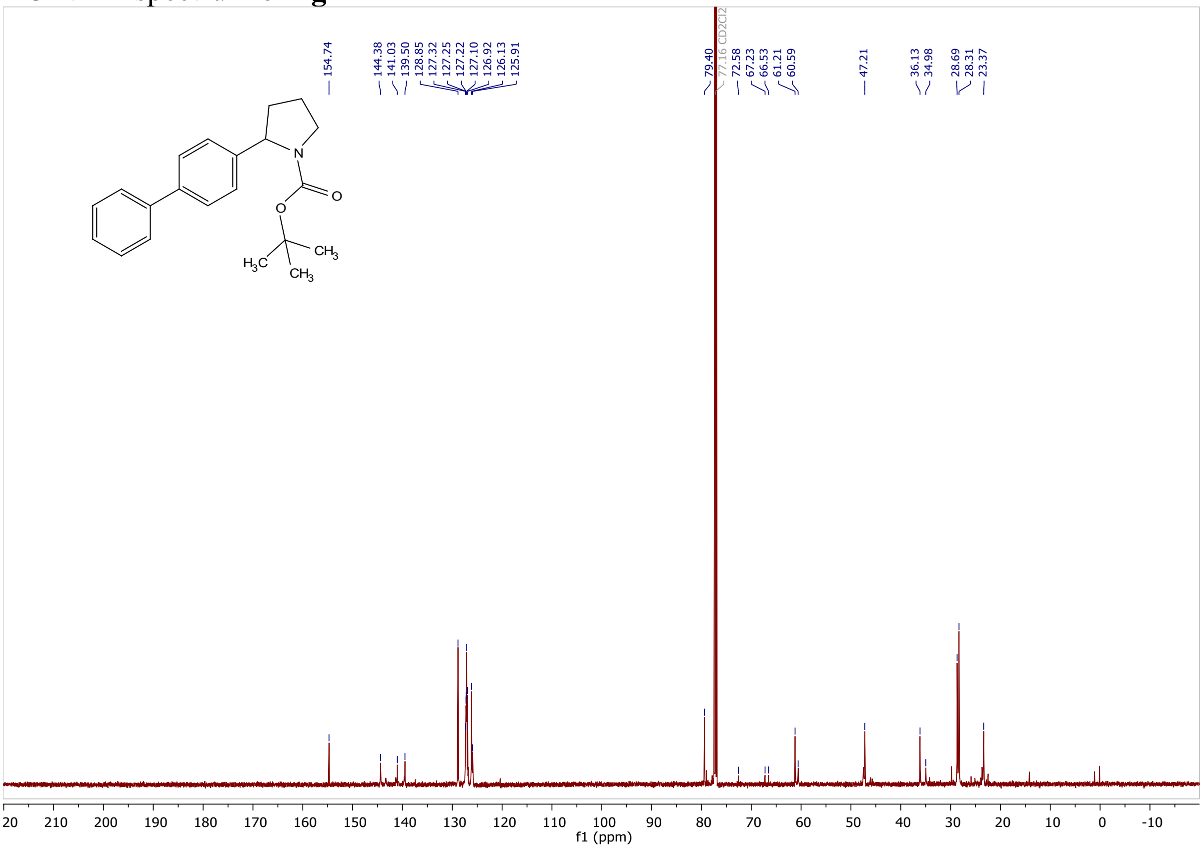

^{1}H NMR spectrum of **4i**

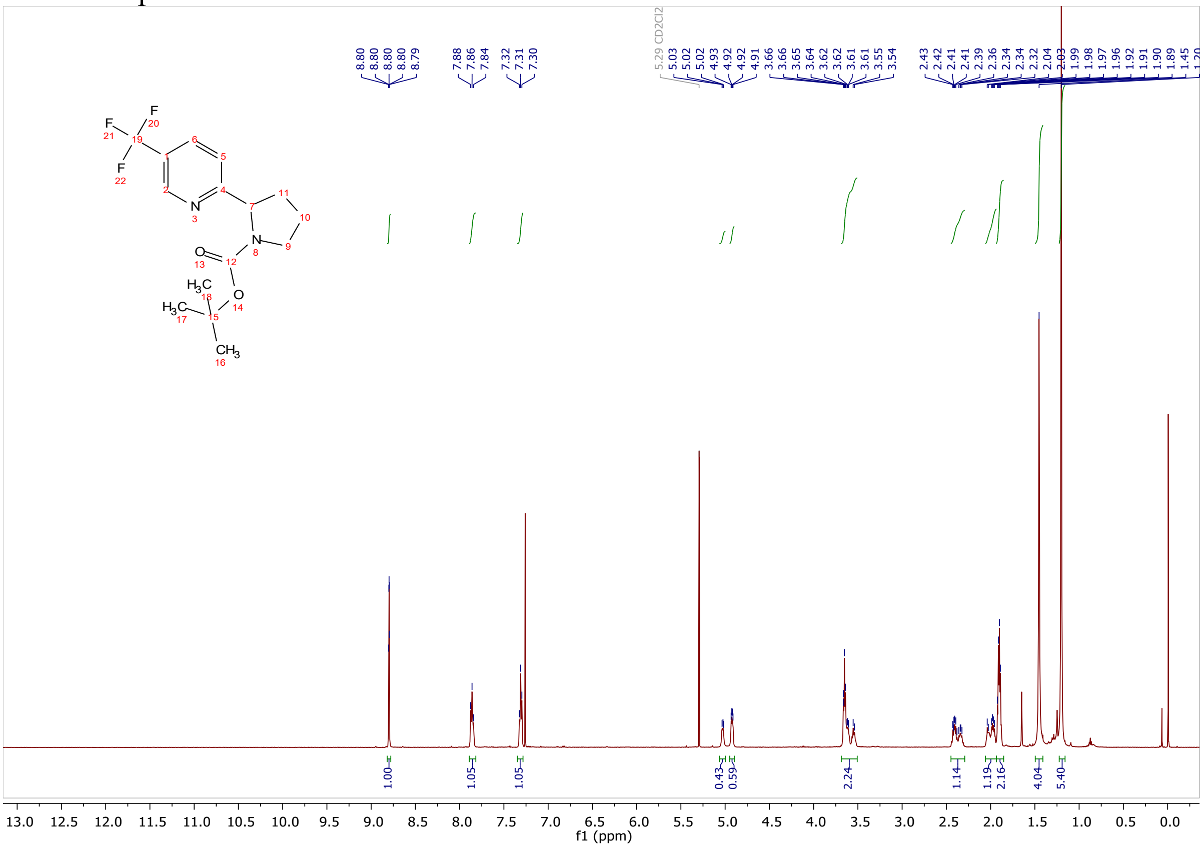

^{13}C NMR spectrum of **4i**

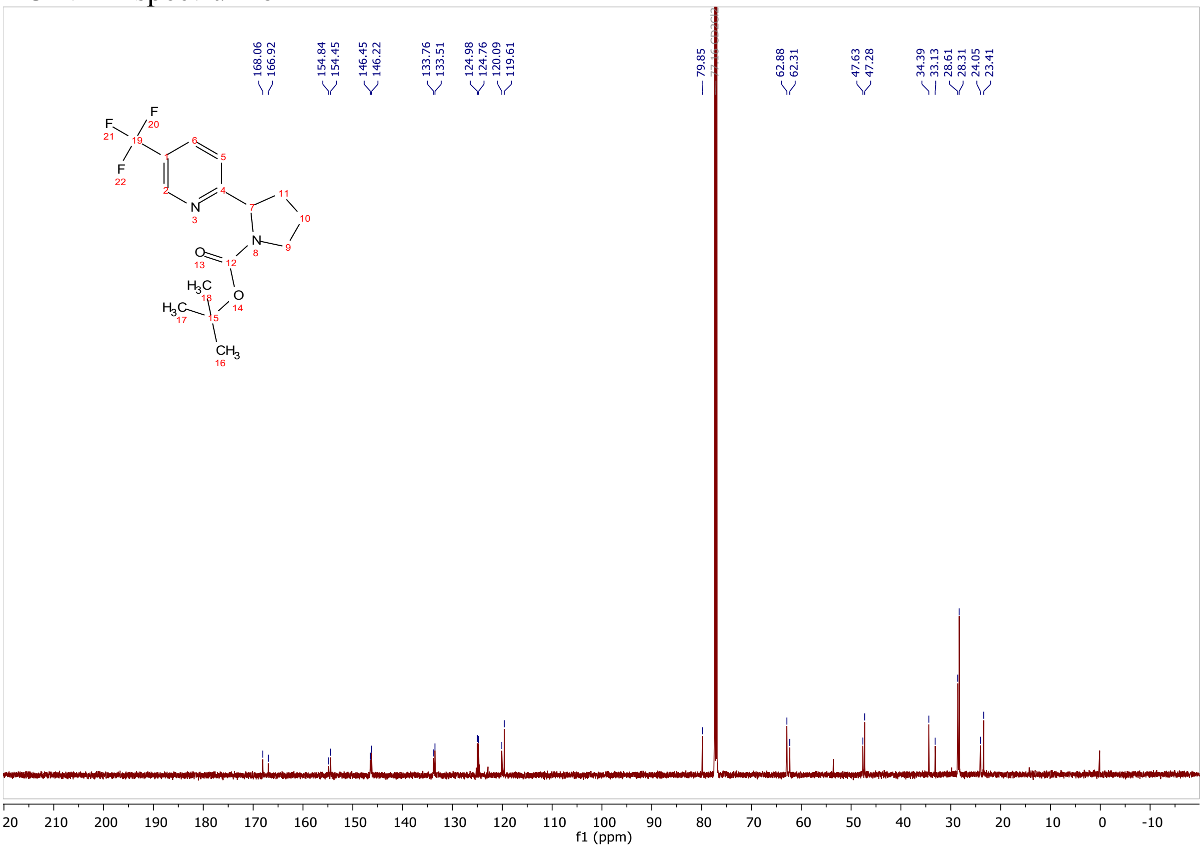

^{19}F NMR spectrum of **4i**

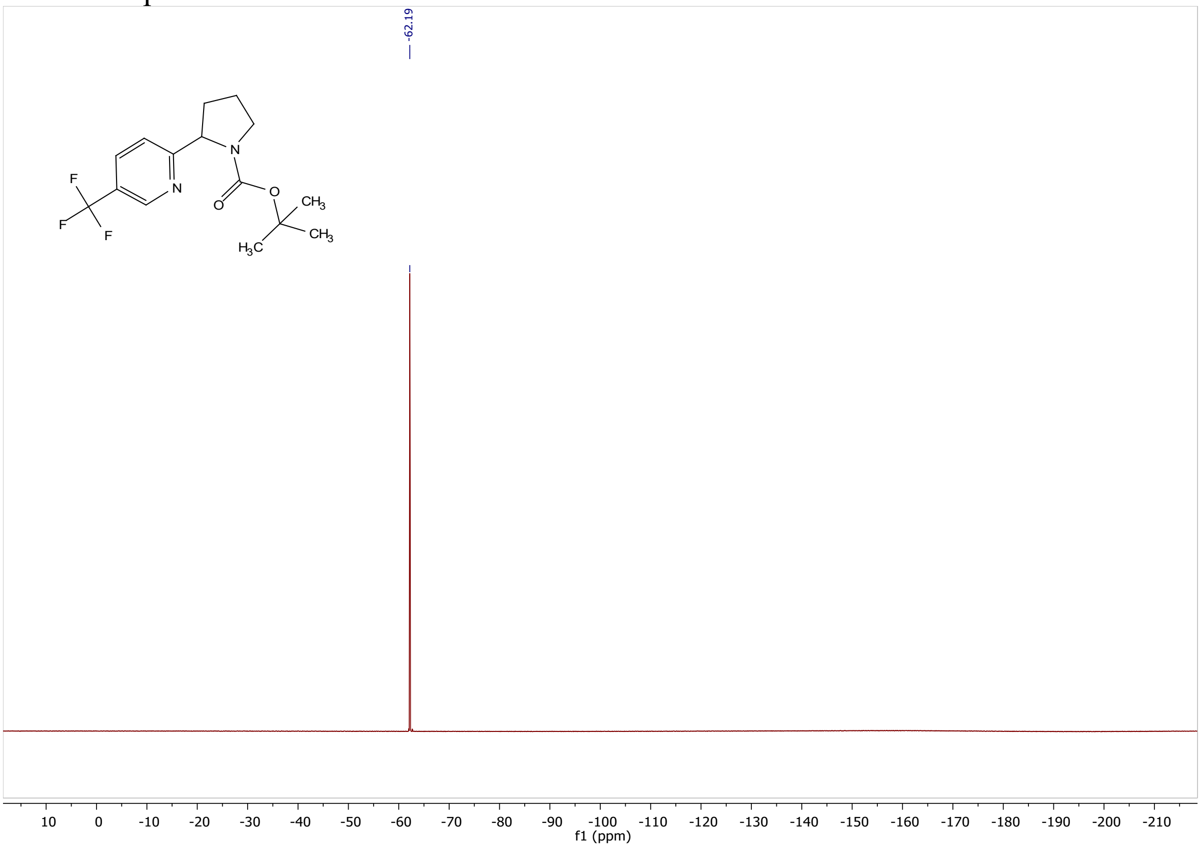

^{1}H NMR spectrum of **4k**

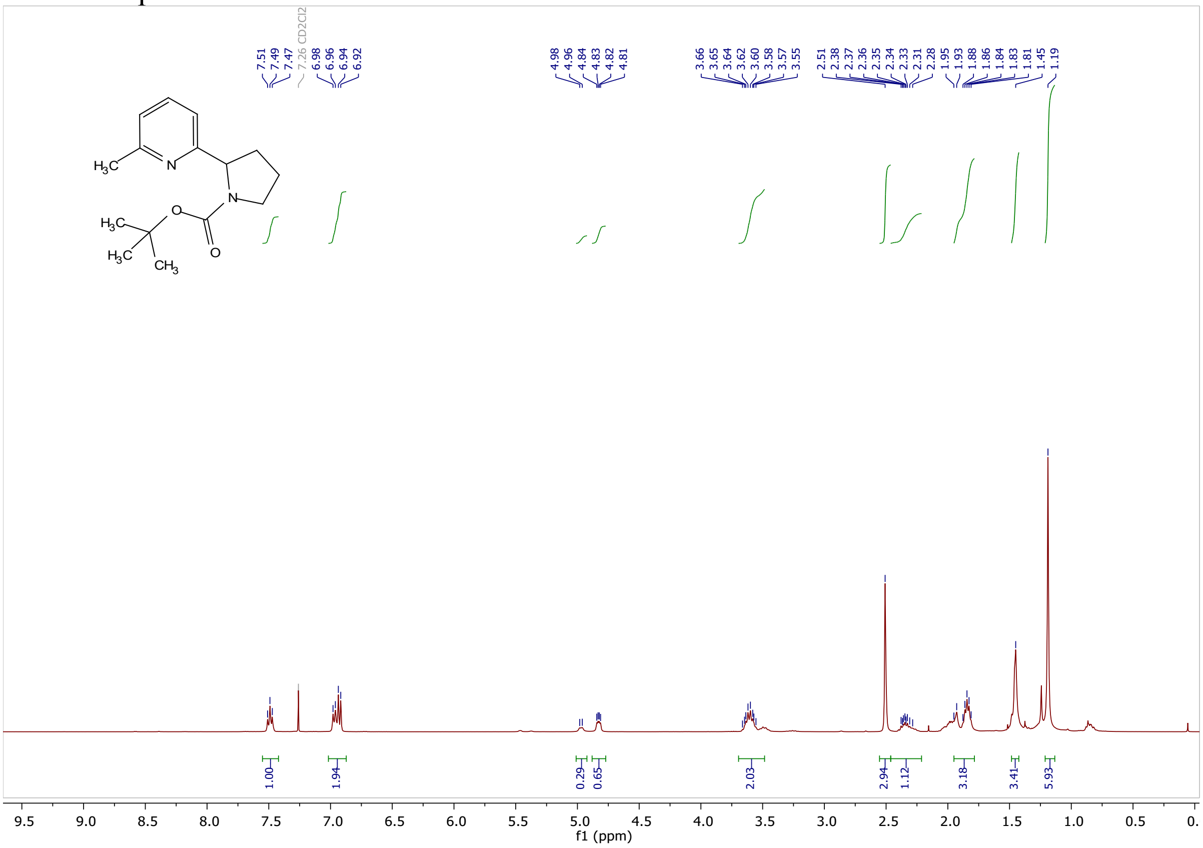

^{13}C NMR spectrum of **4k**

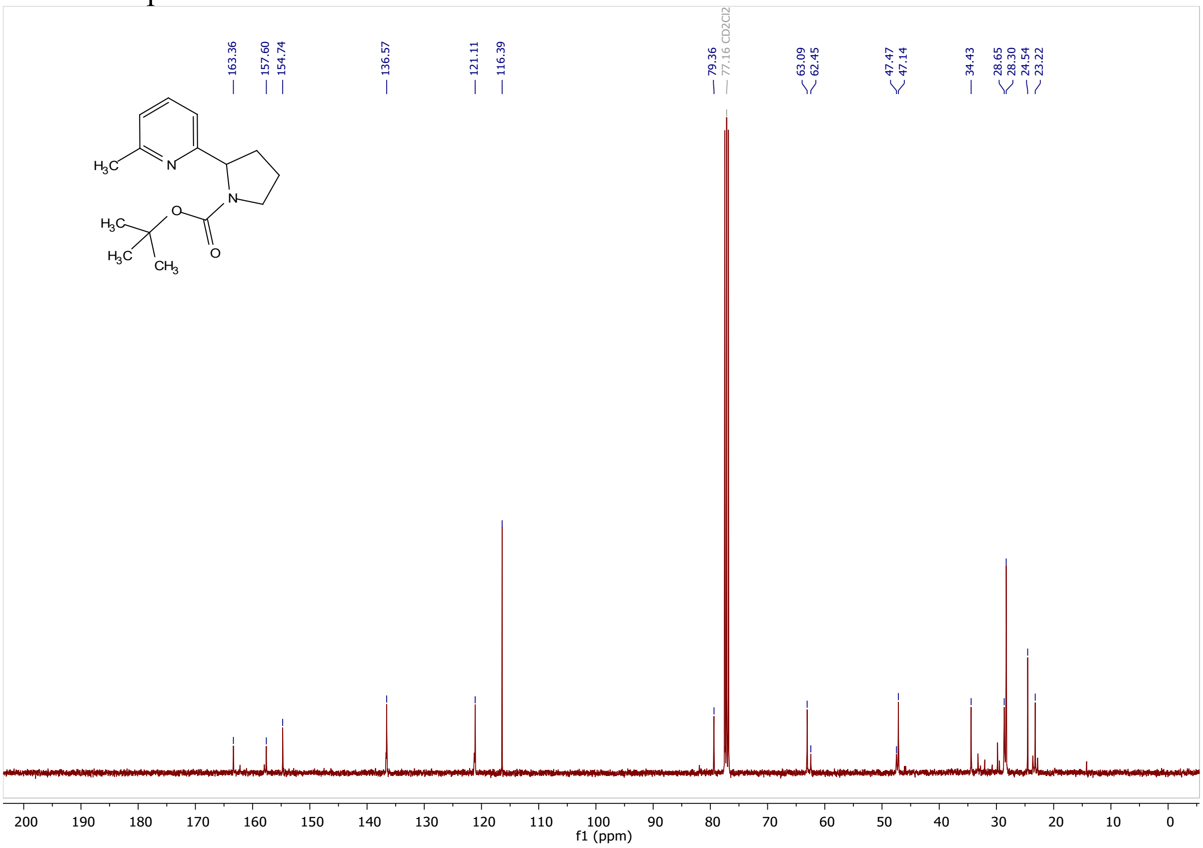

^{1}H NMR spectrum of **4l**

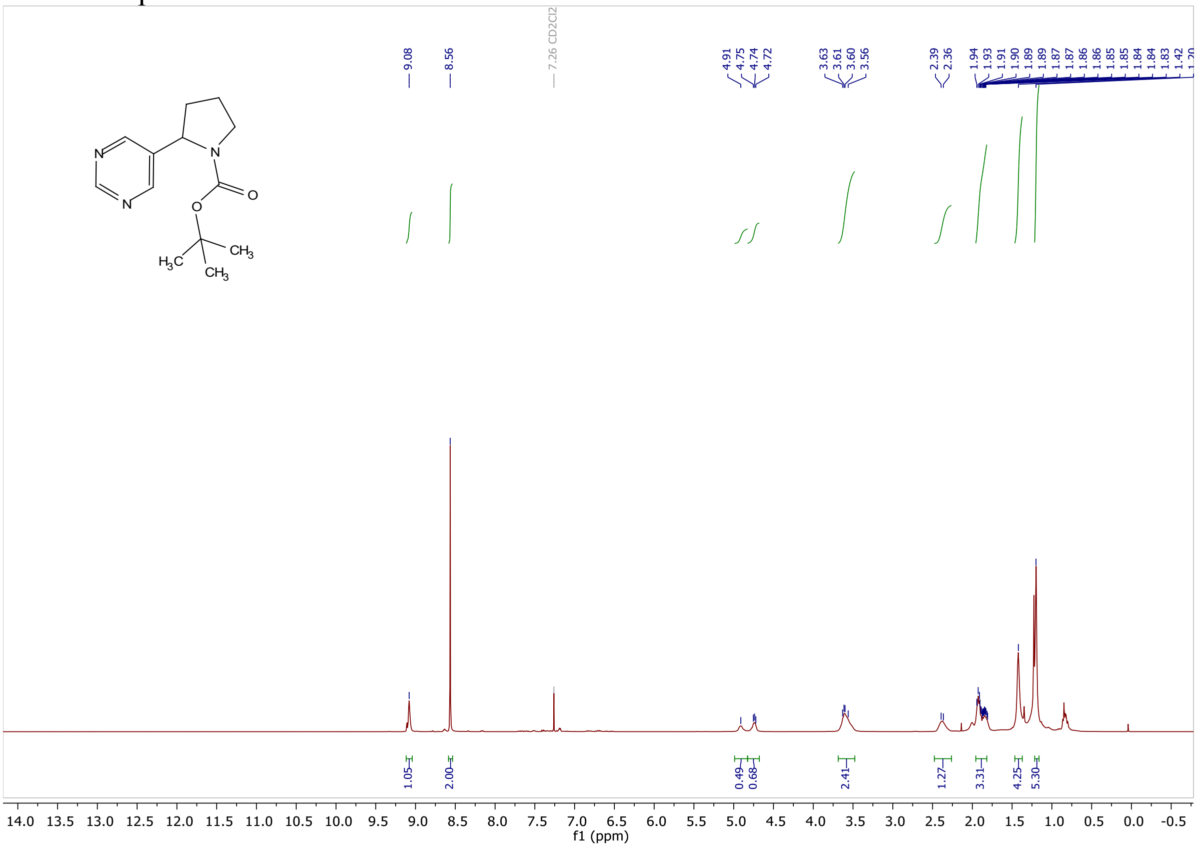

^{13}C NMR spectrum of **4l**

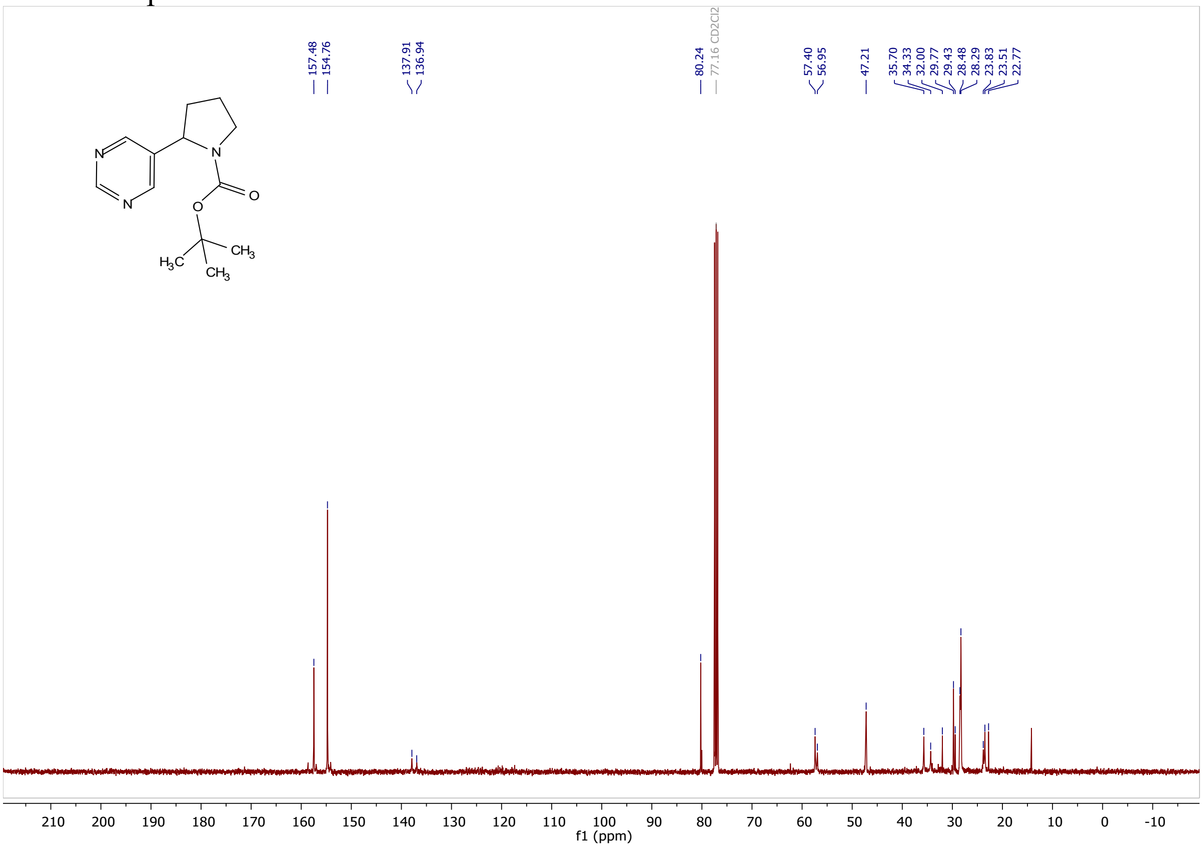

^{1}H NMR spectrum of **4m**

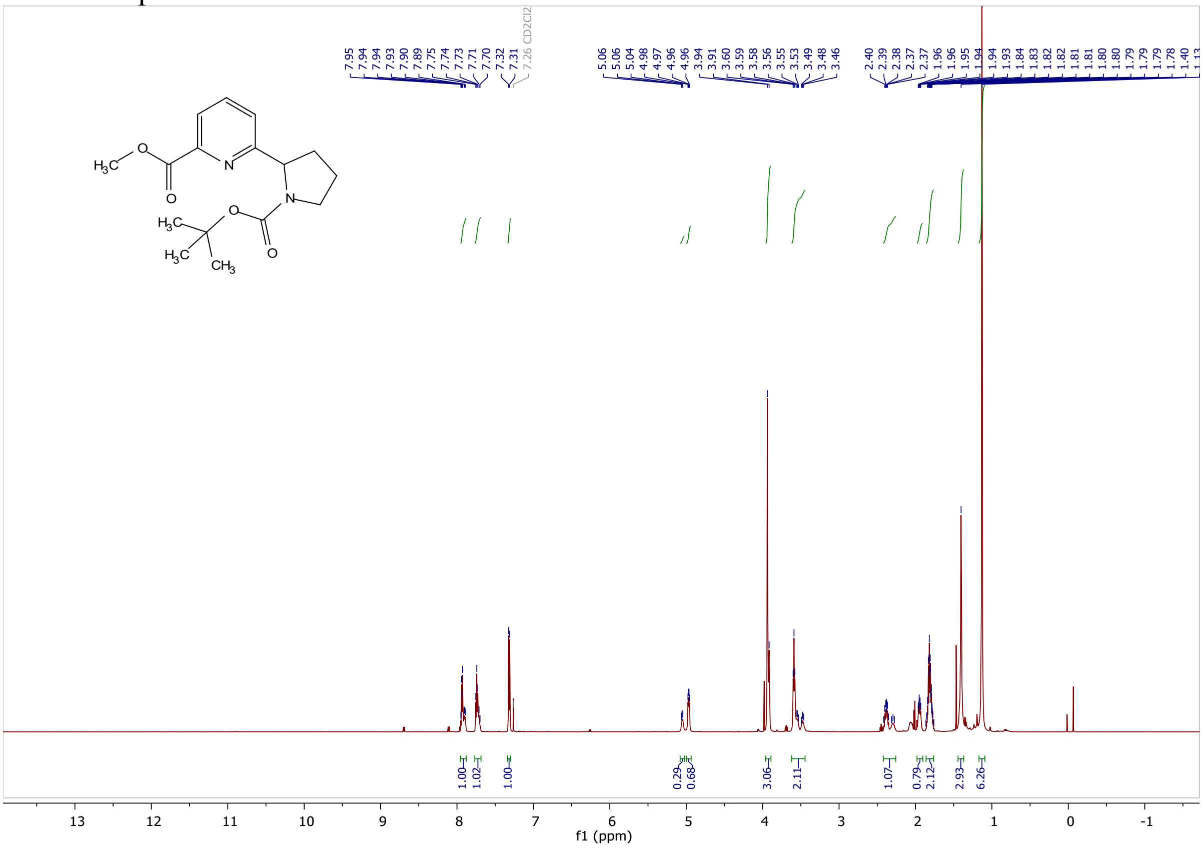

^{13}C NMR spectrum of **4m**

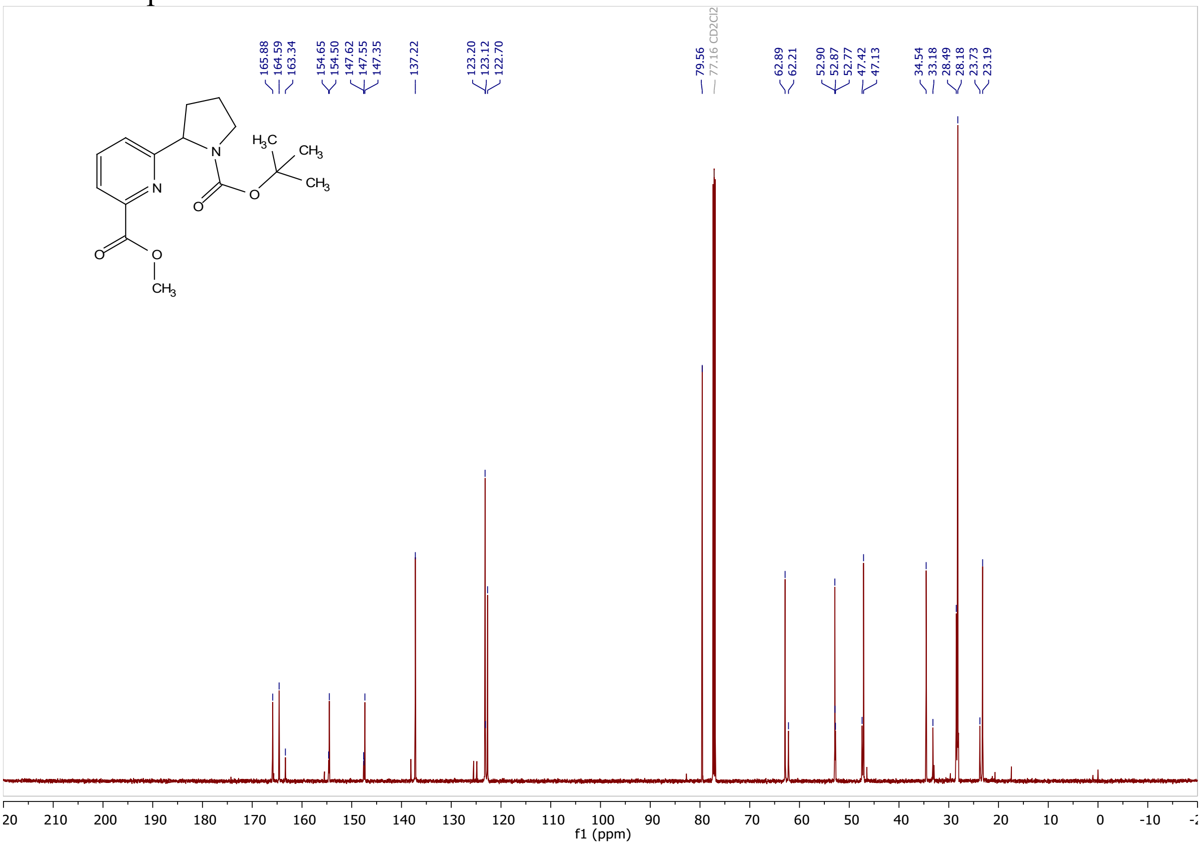

^{1}H NMR spectrum of **4n**

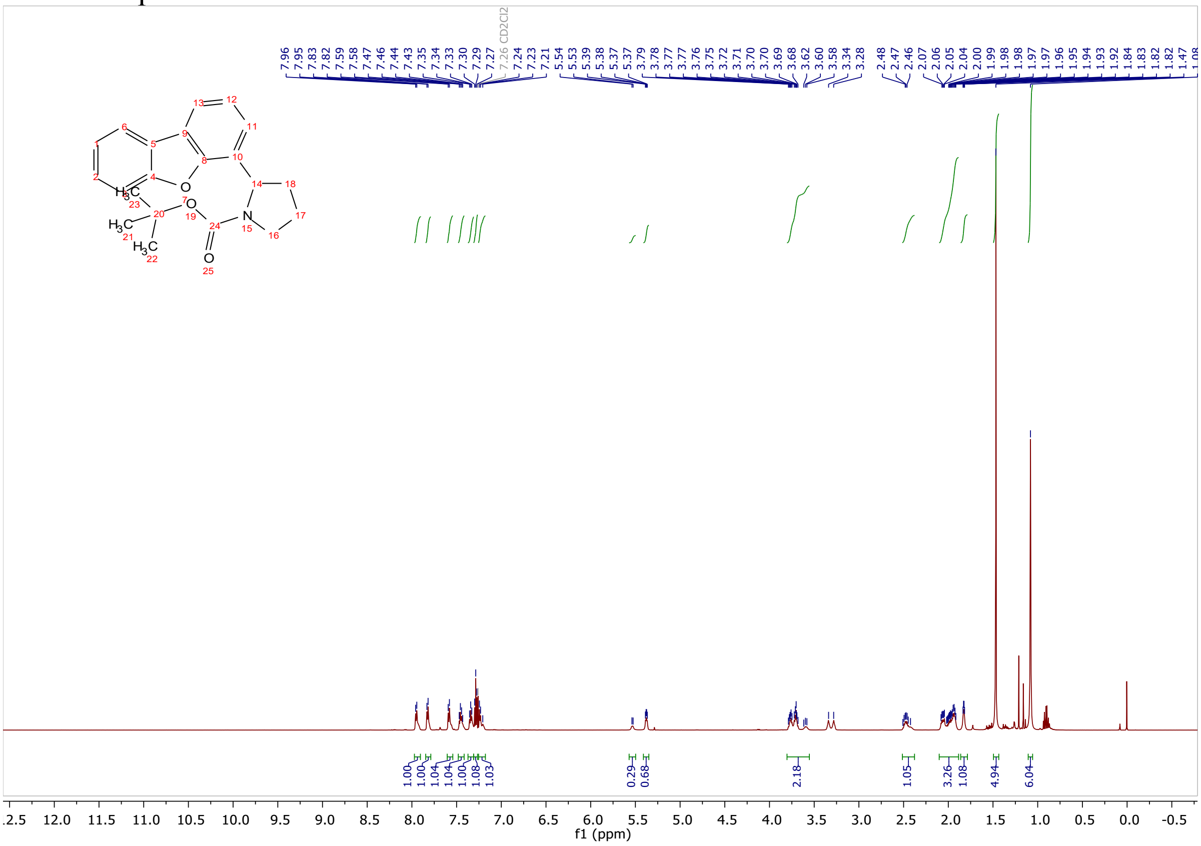

^{13}C NMR spectrum of **4n**

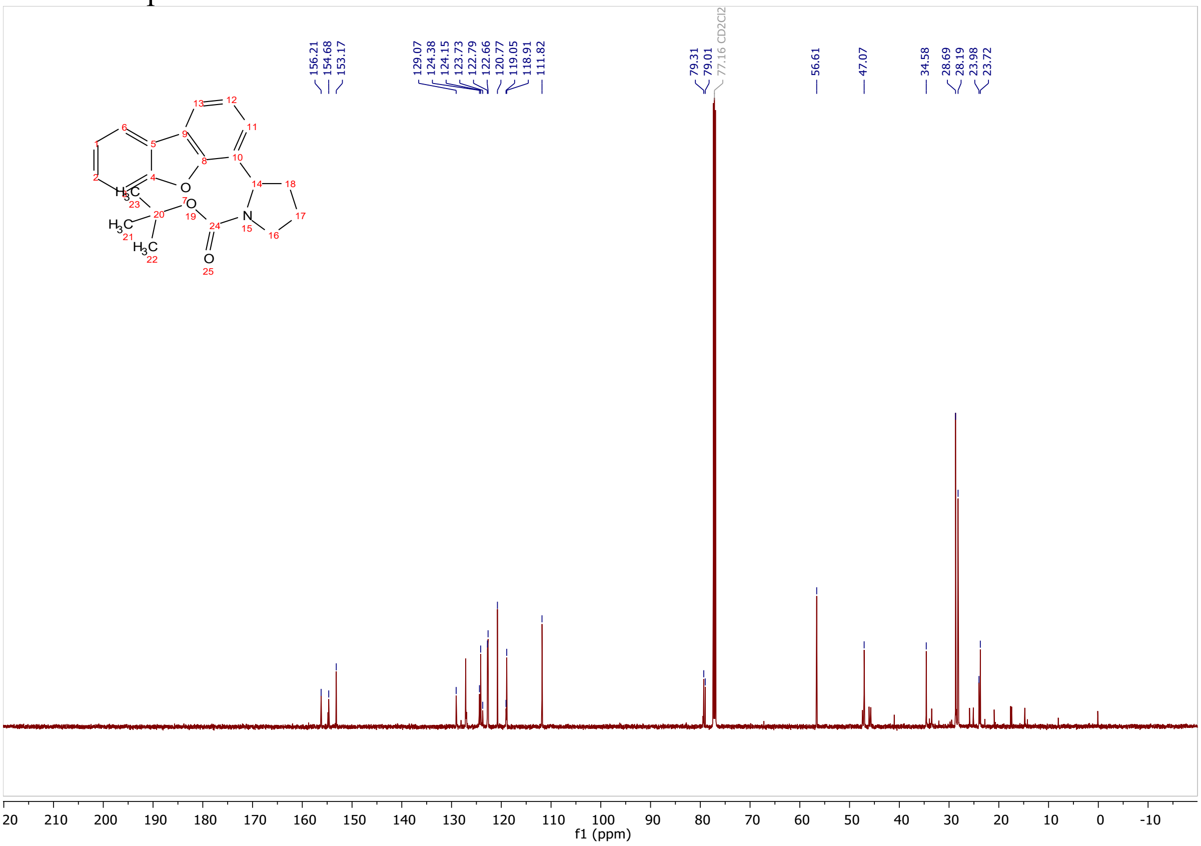

^{1}H NMR spectrum of **4o**

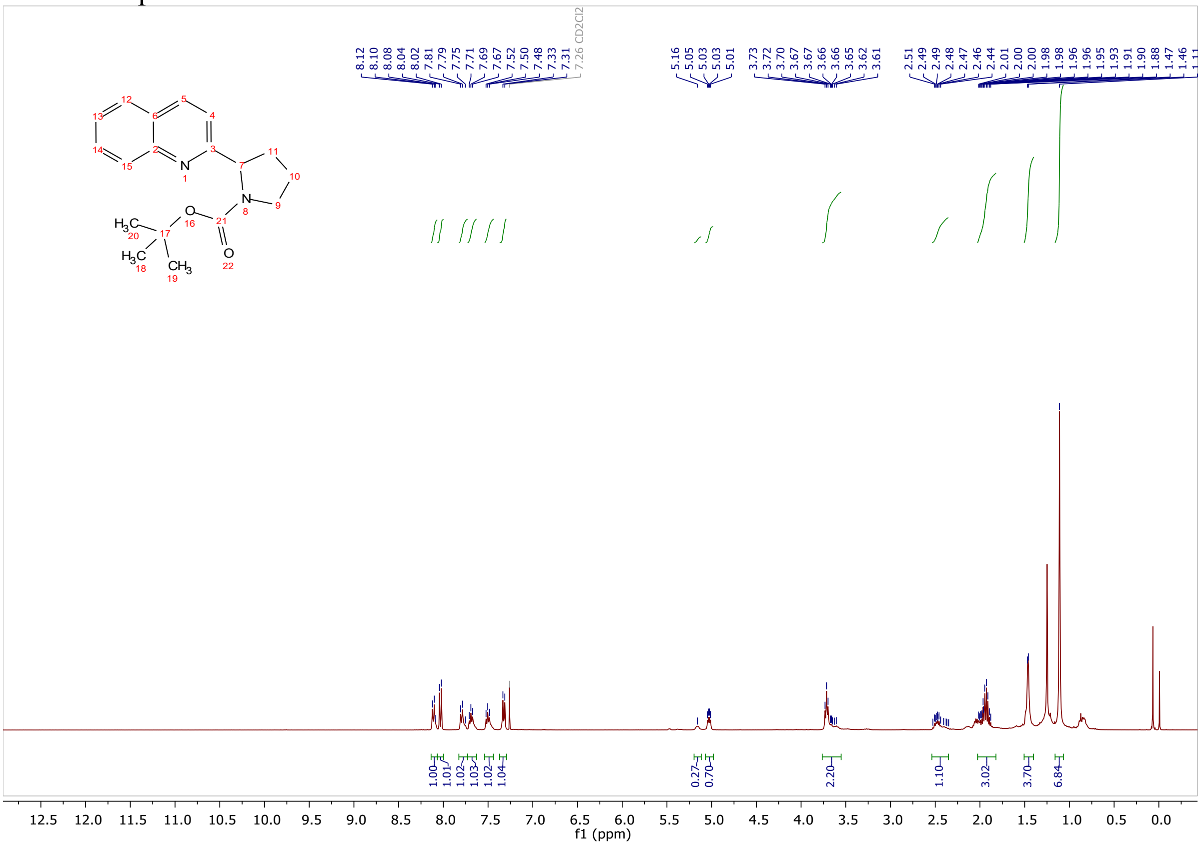

^{13}C NMR spectrum of **4o**

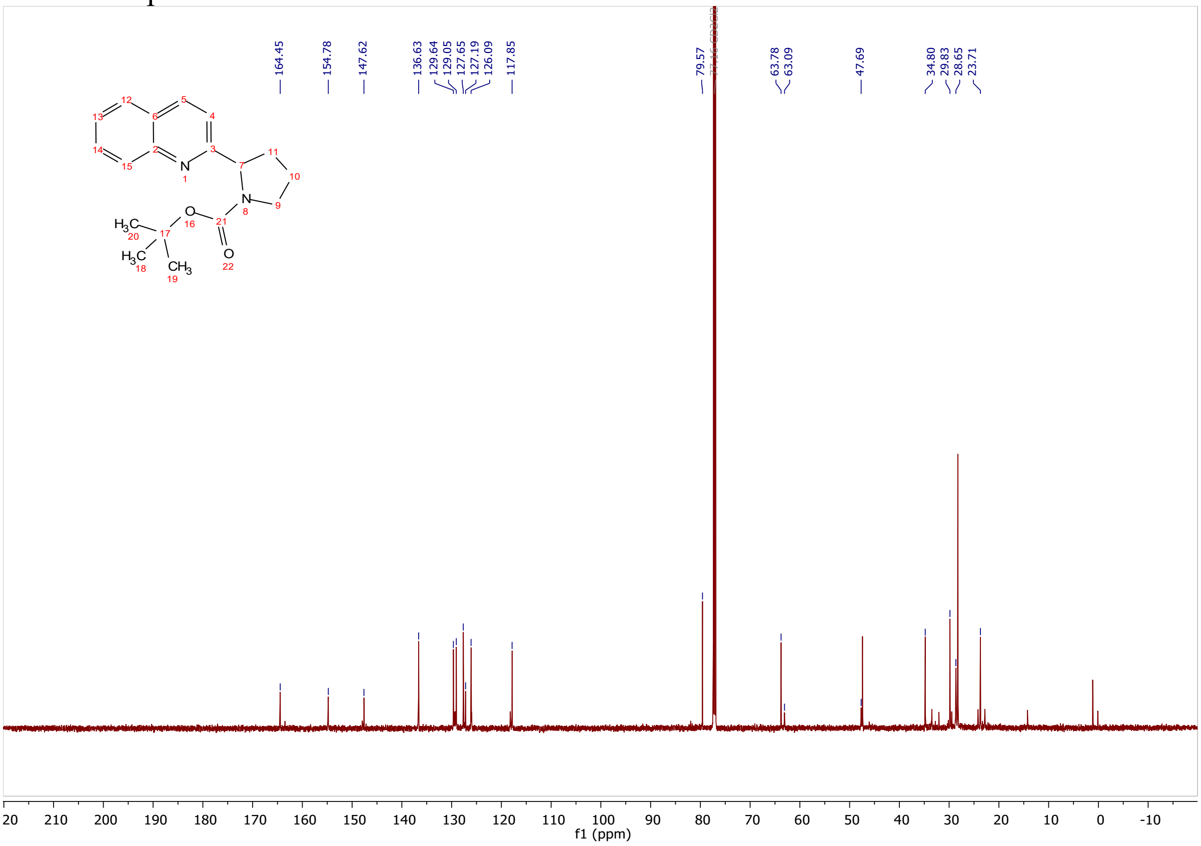

7. DFT calculations

7.1. General information

DFT calculations were performed using Gaussian 16.[21] Gas phase geometry optimizations were done using the B3LYP functional[22] with 6-31G(d)[23] as basis set. At this level of theory, all stationary points were characterized by frequency calculations. For all found maxima, only one imaginary frequency was obtained, and intrinsic reaction coordinate scans (IRC) were performed to verify the energy profiles connecting the transition structures to the correct associated local minima.

The gas-phase structures were reoptimized using B3LYP with added empirical dispersion (GD3BJ)[24] and implicit solvent description (IEFPCM) for 1,4-dioxane in combination with 6-311+G(d,p)[25] as basis set. The temperature for the final frequency calculations was set to 313 K to obtain the final free energy values.

All given values correspond to Gibbs Free (G) energies at this level of theory and are given in kcal mol^{-1}, unless stated otherwise. All relative free energies (ΔG) have been calculated in reference to separately computed, uncoordinated starting compounds (bromine radical, cyclopentylboronic acid).

7.2 Results

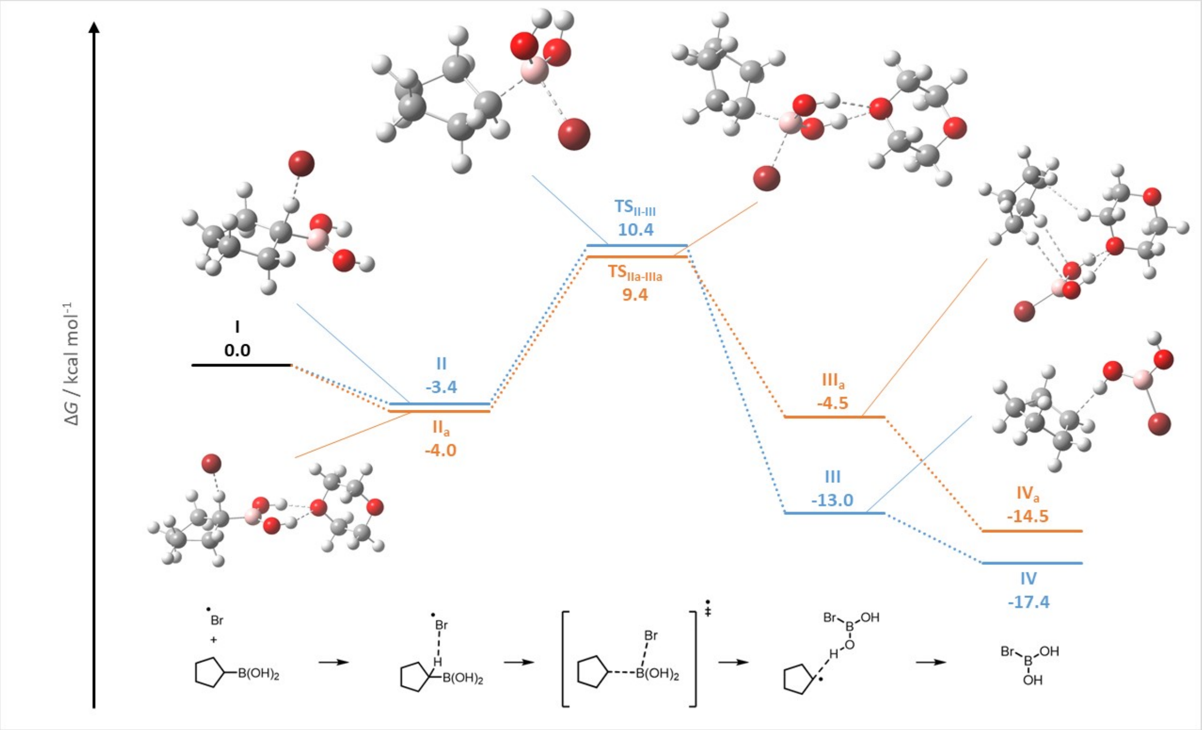

Scheme S1: Free energy diagram for the activation pathways for the boronic acid with (blue) and without (orange, index *a*) explicitly modelled solvent molecule taken into account.

For the activation of the boronic acids, using cyclopentylboronic acid as a prototype, several approaches were computationally explored. None of the attempts at ionic pathways involving a preformed complex of boronic acid and a bromide anion led to any feasible structures.

Complexes of boronic acid with either carbonate or an explicit solvent molecule have been investigated. All approaches involving a bromide anion in this context were unsuccessful.

Moreover, dissociation of these carbonate or dioxane complexes preceding the bromide approach has been examined but did not lead to any feasible transition structures, neither via radical nor ionic pathways, which is in agreement with the experimentally determined crucial need for the presence of bromide.

In contrast, feasible pathways were found for the involvement of a bromine radical. While the activation mechanism initiated by the reaction of a bromine radical with residual water was found at a high energy barrier (31.7 kcal mol^{-1}), the activation by a direct approach of a bromine radical to the starting boronic acid, both with and without an explicitly modelled dioxane molecule were found more promising. The energy diagram corresponding to that pathway is

depicted in Scheme S1 (indicated in blue: pathway with implicit solvent model only; indicated in orange and denoted with index *a*: pathway both with explicitly described dioxane molecule and implicit solvent model). In contrast to the bromine radical forming a boronate complex, the calculations suggest a favorable coordination towards one of the cyclopentyl hydrogens (intermediates **II** and $\mathbf{II_a}$). The activation barriers for the subsequent transition states $\mathbf{TS_{II\text{-}III}}$ and $\mathbf{TS_{IIa\text{-}IIIa}}$ were found to be reasonably low at 9.4 kcal mol^{-1} and 10.4 kcal mol^{-1}, respectively, to lead to bromoboronic acid **IV/IVa** via an overall clearly exothermic pathway.

The explicitly modelled solvent molecule was found to be coordinated to all boronic acid intermediates by H-bonding, but overall had only a very minor positive effect (about 1 kcal mol^{-1}) on the energies in the pathway, and the relevant geometries were not altered significantly in comparison to the ones obtained for the implicit description in the implicit polarizable continuum model. The only exception from this was found to be the intermediary secondary radical complex $\mathbf{III_a}$, where the solvent substitutes the stabilizing interaction between the hydrogen of the boronic acid and the radical by H-bonding to the solvent oxygen. Expectedly, this destabilization renders the secondary radical more reactive towards the Ni catalyst system (which was not taken into account for the calculations performed in this work, as this was considered a well-established and commonly known process). As the explicit solvent model seemed not to significantly impact the interactions of interest to this work, it was omitted in the main text and in the discussion of the results in the following.

To complement the study on the boronic acid, the transition state was calculated for the activation step employing pinacol cyclopentyl boronate, where the TS was found at a significantly elevated energetic barrier of 48.7 kcal mol^{-1}, which may to a large part be the result of a steric clash between the cyclopentyl ring and the pinacol moiety in the TS geometry needed to provide space for the bromine radical to approach. There are also some implications on an electronic level: As the analysis of the NBO charge distribution in the calculated TS shows, there are higher partial charges at the oxygens of the free boronic acid (-0.905 vs -0.801), which reflect also in a slightly higher partial charge the *ipso*-carbon atom (-0.347 vs. -0.351 for the pinacol boronate). Coordinating a dioxane molecule to the boronic acid has an enhancing effect in both regards (-0.924 and -0.332 for oxygens and carbon, respectively). Overall, these findings reflect the experimental results showing that the reaction works exclusively with boronic acids and not with their ester counterparts.

All found productive pathways for the activation of the cyclopentylboronic acid resulted in the formation of the bromoboronic acid intermediate **IV**. Several computational attempts to transform this intermediate into any more plausible reaction products were undertaken, such as elimination of HBr with and without base (both carbonate and hydroxide as generic base from residual water in the reaction media) to deliver borinic acid or the addition of hydroxide or carbonate to form an anionic compound prior to eliminating bromide. However, none of these investigations resulted in any useful transition structures or stable products.

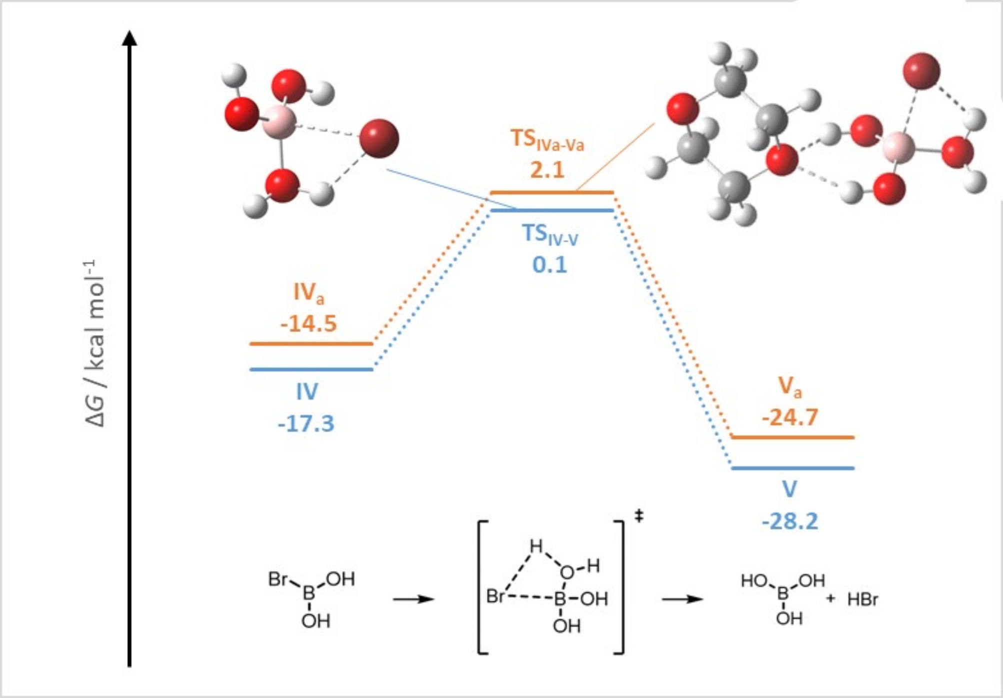

Scheme S2: Free energy diagram for the transformation of bromoboronic acid **IV** into boronic acid (**V**), with (blue) and without (orange, index *a*) explicitly modelled solvent molecule taken into account. Energies are relative to the uncoordinated, separately calculated starting materials (**I** in Scheme S1).

Finally, the addition of residual water was examined and resulted in a computationally feasible transition state in which the water molecule replaces the bromide and simultaneously transfers one of the protons to yield boronic acid and hydrogen bromide in one single step. The corresponding results are depicted in the energy diagram in Scheme 2. The relative barrier to the obtained $\mathbf{TS_{IV\text{-}V}}$ was found to be surprisingly high ($\Delta\Delta G$(**IV**, $\mathbf{TS_{IV\text{-}V}}$) = 17.4 kcal mol^{-1}) due

to the unexpected stability of the bromoboronic acid intermediate **IV** (-17.3 kcal mol^{-1}), however, for a process at 40 °C, this is to be considered quite plausible. As mentioned before, the consideration of an explicitly modelled solvent molecule did not provide any significant effect in terms of energy (16.6 kcal mol^{-1} activation barrier for $\mathbf{TS_{IVa\text{-}Va}}$).

The resulting boronic acid (**V**) and hydrogen bromide were calculated to be at a significantly lower energy level than any other intermediate in the found pathway (-28.2 kcal mol^{-1}) suggesting a strong overall driving force for entire pathway. The computationally suggested boronic acid as reaction byproduct could be experimentally proven by ^{11}B-NMR spectroscopy.

7.3 Energies and coordinates of calculated structures

Boronic acid

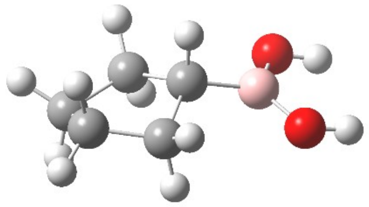

Method: IEFPCM(1,4-dioxane)-B3LYP-GD3BJ/6-311+G(d,p)

Electronic Energy (EE) = -372.71495 Hartree
Zero-point Energy Correction = 0.164182 Hartree
Thermal Correction to Energy = 0.173448 Hartree
Thermal Correction to Enthalpy = 0.174439 Hartree
Thermal Correction to Free Energy = 0.127509 Hartree
EE + Zero-point Energy = -372.55077 Hartree
EE + Thermal Energy Correction = -372.5415 Hartree
EE + Thermal Enthalpy Correction = -372.54051 Hartree
EE + Thermal Free Energy Correction = -372.58744 Hartree

B	1.55212300	-0.01596300	0.06694500
O	2.22903700	1.18098800	0.03683700
H	3.16476500	1.12854500	-0.18291300
O	2.15820100	-1.21741700	-0.21563300
H	3.09635400	-1.17338000	-0.42735900
C	0.03223600	-0.00854900	0.46173000
C	-0.77813200	1.24792000	0.01378700
C	-0.80632000	-1.20997200	-0.02603700
H	0.01273700	-0.03415900	1.56233600
C	-2.19596300	0.73367000	-0.36141700
H	-0.30723200	1.74092900	-0.83945700
H	-0.81596400	1.98903900	0.81467800
C	-2.25142500	-0.72338900	0.13330800
H	-0.59079100	-2.12580500	0.52834300
H	-0.59790200	-1.41533300	-1.08247600
H	-2.99081500	1.35114200	0.06346200
H	-2.32334000	0.75339200	-1.44817200
H	-2.53073700	-0.75153000	1.19294500
H	-2.97797000	-1.32968700	-0.41397400

Bromine radical

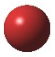

Method: IEFPCM(1,4-dioxane)-UB3LYP-GD3BJ/6-311+G(d,p)

Electronic Energy (EE) = -2574.1064 Hartree
Zero-point Energy Correction = 0 Hartree
Thermal Correction to Energy = 0.001487 Hartree
Thermal Correction to Enthalpy = 0.002478 Hartree
Thermal Correction to Free Energy = -0.017789 Hartree
EE + Zero-point Energy = -2574.1064 Hartree
EE + Thermal Energy Correction = -2574.1049 Hartree
EE + Thermal Enthalpy Correction = -2574.1039 Hartree
EE + Thermal Free Energy Correction = -2574.1241 Hartree

Br	0.00000000	0.00000000	0.00000000

1,4-Dioxane

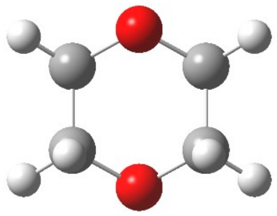

Method: IEFPCM(1,4-dioxane)-B3LYP-GD3BJ/6-311+G(d,p)

Electronic Energy (EE) = -307.77226 Hartree
Zero-point Energy Correction = 0.121997 Hartree
Thermal Correction to Energy = 0.12769 Hartree
Thermal Correction to Enthalpy = 0.128681 Hartree
Thermal Correction to Free Energy = 0.091866 Hartree
EE + Zero-point Energy = -307.65026 Hartree
EE + Thermal Energy Correction = -307.64457 Hartree
EE + Thermal Enthalpy Correction = -307.64358 Hartree
EE + Thermal Free Energy Correction = -307.68039 Hartree

C	1.17446400	-0.73535600	0.19410200
C	-1.17446500	-0.73535800	0.19409700
C	-1.17445900	0.73535900	-0.19410300
C	1.17445700	0.73536000	-0.19410900
H	-2.02740100	-1.25492300	-0.24594300
H	1.22305000	-0.83150000	1.28806100
H	2.02741500	-1.25491500	-0.24591800
H	-1.22303900	0.83152400	-1.28806100
H	-2.02739800	1.25493000	0.24592200
H	1.22301900	0.83150600	-1.28806800
H	2.02740700	1.25493200	0.24589600
H	-1.22306600	-0.83152300	1.28805400

O	0.00000100	1.38475700	0.29226800
O	0.00000300	-1.38476500	-0.29225100

II

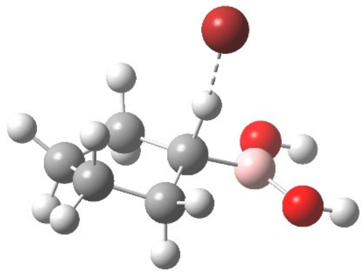

Method: IEFPCM(1,4-dioxane)-UB3LYP-GD3BJ/6-311+G(d,p)

Electronic Energy (EE) = -2946.836 Hartree
Zero-point Energy Correction = 0.160923 Hartree
Thermal Correction to Energy = 0.172503 Hartree
Thermal Correction to Enthalpy = 0.173494 Hartree
Thermal Correction to Free Energy = 0.118901 Hartree
EE + Zero-point Energy = -2946.675 Hartree
EE + Thermal Energy Correction = -2946.6634 Hartree
EE + Thermal Enthalpy Correction = -2946.6625 Hartree
EE + Thermal Free Energy Correction = -2946.7171 Hartree

B	1.19069300	1.58284100	0.03641800
O	1.41901100	2.20326700	-1.15982300
H	1.56858000	3.15377400	-1.12415600
O	1.18890900	2.20428800	1.25360500
H	1.33408700	3.15603100	1.24632900
C	0.88683300	0.03412500	0.01111800
C	1.28824100	-0.80957500	-1.20667900
C	1.16357800	-0.81243500	1.24904300
H	-0.31033400	0.12383400	-0.03208000
C	1.64070500	-2.21282500	-0.65270000
H	2.12793500	-0.34699700	-1.73296600
H	0.47066600	-0.85402400	-1.93050500
C	1.01573200	-2.25640000	0.75274400
H	0.52277200	-0.55479600	2.09334900
H	2.20009300	-0.62951300	1.56763200
H	1.28491800	-3.01681600	-1.29926000
H	2.72736900	-2.31602700	-0.57302900
H	-0.04739000	-2.50940300	0.68872400
H	1.49340000	-2.98631600	1.40946200
Br	-2.17598500	-0.00147800	-0.06191400

II$_a$

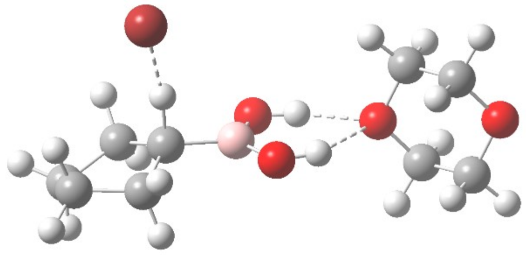

Method: IEFPCM(1,4-dioxane)-UB3LYP-GD3BJ/6-311+G(d,p)

Electronic Energy (EE) = -3254.6281 Hartree
Zero-point Energy Correction = 0.285292 Hartree
Thermal Correction to Energy = 0.304302 Hartree
Thermal Correction to Enthalpy = 0.305293 Hartree
Thermal Correction to Free Energy = 0.229708 Hartree
EE + Zero-point Energy = -3254.3428 Hartree
EE + Thermal Energy Correction = -3254.3238 Hartree
EE + Thermal Enthalpy Correction = -3254.3228 Hartree
EE + Thermal Free Energy Correction = -3254.3984 Hartree

B	0.24847100	-0.78068000	0.16935000
O	-0.27810100	-0.85641500	1.42790700
H	-1.24347400	-0.74044000	1.42253100
O	-0.51095500	-0.60089000	-0.95281300
H	-1.45286600	-0.50144100	-0.73296000
C	1.81752100	-0.87995000	0.00206900
C	2.67796100	-1.49264200	1.11363500
C	2.41467400	-1.33322400	-1.32434100
H	2.02303200	0.30874700	0.03955400
C	3.86625000	-2.18365800	0.39928300
H	2.08737000	-2.19087300	1.71365700
H	3.01872800	-0.71923100	1.80689600
C	3.89832900	-1.56350500	-1.00852600
H	2.22661400	-0.63164200	-2.13820300
H	1.93982000	-2.28390800	-1.60809400
H	4.80674900	-2.05950500	0.93903600
H	3.67555100	-3.25868900	0.32077100
H	4.41702400	-0.59967400	-0.98729500
H	4.40061100	-2.19688400	-1.74273900
Br	2.60141900	2.07835600	0.02349100
C	-3.40149200	1.01805700	0.69431100

C	-4.35657800	1.36462700	-0.43429600
C	-4.92771500	-0.89999900	-0.68005900
C	-3.98116700	-1.27799200	0.44633800
H	-4.80541300	2.34465000	-0.26815100
H	-3.90825400	1.08411000	1.66345100
H	-2.53026500	1.67383800	0.69544400
H	-5.79129100	-1.56615200	-0.69477200
H	-4.40661800	-0.97085300	-1.64514600
H	-4.50967400	-1.29176700	1.40613800
H	-3.52242300	-2.25199800	0.27159600
H	-3.81650400	1.37838500	-1.39113800
O	-2.90373600	-0.32483800	0.52226500
O	-5.42563700	0.42264200	-0.49610600

$TS_{II\text{-}III}$

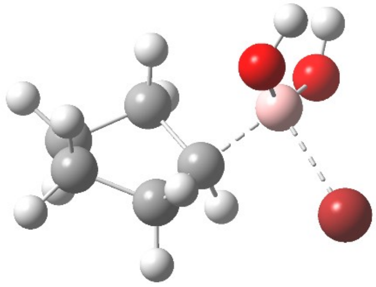

Method: IEFPCM(1,4-dioxane)-UB3LYP-GD3BJ/6-311+G(d,p)

Electronic Energy (EE) = -2946.8167 Hartree
Zero-point Energy Correction = 0.161953 Hartree
Thermal Correction to Energy = 0.173376 Hartree
Thermal Correction to Enthalpy = 0.174367 Hartree
Thermal Correction to Free Energy = 0.12177 Hartree
EE + Zero-point Energy = -2946.6548 Hartree
EE + Thermal Energy Correction = -2946.6434 Hartree
EE + Thermal Enthalpy Correction = -2946.6424 Hartree
EE + Thermal Free Energy Correction = -2946.695 Hartree

B	0.40856200	0.97904600	0.24679500
O	0.18628800	1.06294300	1.60513600
H	0.54798800	1.84295700	2.03626200
O	0.74499800	2.04684600	-0.56045300
H	0.77315900	2.90171200	-0.11944600
C	-0.80985500	-0.05072600	-0.52286900
C	-1.24039200	-1.28000800	0.29347400
C	-2.02023500	0.91558400	-0.58303600
H	-0.45823200	-0.29602100	-1.51997200
C	-2.75179100	-1.08336600	0.55682200
H	-0.68050400	-1.37058100	1.22352900
H	-1.04310100	-2.18605500	-0.28453400

C	-3.20151200	-0.06921700	-0.50719700
H	-1.99211000	1.52922800	-1.48309000
H	-2.05709800	1.57812000	0.28469800
H	-3.30789100	-2.02157800	0.51084700
H	-2.90436200	-0.65887400	1.55327100
H	-3.33593400	-0.56450600	-1.47414000
H	-4.13629800	0.43844900	-0.25536100
Br	1.97840000	-0.61600000	-0.15676200

$TS_{IIa\text{-}IIIa}$

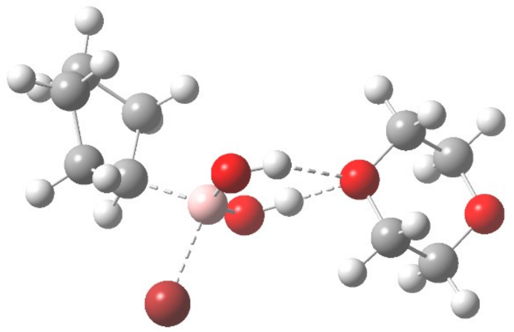

Method: IEFPCM(1,4-dioxane)-UB3LYP-GD3BJ/6-311+G(d,p)

Electronic Energy (EE) = -3254.609 Hartree
Zero-point Energy Correction = 0.286655 Hartree
Thermal Correction to Energy = 0.305287 Hartree
Thermal Correction to Enthalpy = 0.306279 Hartree
Thermal Correction to Free Energy = 0.232061 Hartree
EE + Zero-point Energy = -3254.3224 Hartree
EE + Thermal Energy Correction = -3254.3037 Hartree
EE + Thermal Enthalpy Correction = -3254.3027 Hartree
EE + Thermal Free Energy Correction = -3254.377 Hartree

B	-0.73922300	-0.07595000	-0.11865500
O	-0.41736000	0.28538800	1.16880100
H	0.52183900	0.52288800	1.24323700
O	0.12859700	0.03856400	-1.18005200
H	1.00946000	0.31763200	-0.87813400
C	-2.38333600	0.45801900	-0.55829600
C	-3.40933700	0.38233100	0.58257600
C	-2.14201200	1.95377800	-0.87206500
H	-2.67228100	-0.09742200	-1.44459900
C	-3.81044000	1.84671300	0.86608600
H	-3.00605700	-0.10613400	1.46864400
H	-4.26647300	-0.21290500	0.25576900
C	-3.47405900	2.59161200	-0.43493400
H	-1.88539700	2.10174600	-1.92101800
H	-1.33648200	2.37210400	-0.26425700

H	-4.85862400	1.94435300	1.15556900
H	-3.20352700	2.24685300	1.68371500
H	-4.24417200	2.40372400	-1.19024300
H	-3.38501400	3.67356500	-0.30674800
Br	-1.46421600	-2.25088200	-0.09198400
C	3.00586800	-0.70316000	0.76610100
C	4.11336800	-0.89182600	-0.25515300
C	4.32502100	1.43500400	-0.49371700
C	3.22069700	1.65657700	0.52587800
H	4.69888300	-1.78239200	-0.02386800
H	3.41568800	-0.67942000	1.78181800
H	2.25768500	-1.49335700	0.69491000
H	5.06373000	2.23574700	-0.43861000
H	3.89876400	1.41277000	-1.50659200
H	3.64142500	1.76380200	1.53209900
H	2.62813400	2.54019200	0.28547600
H	3.68240100	-1.00080500	-1.25982800
O	2.31392400	0.53889800	0.51720100
O	5.01347500	0.21507400	-0.23263600

III

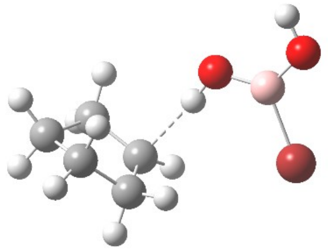

Method: IEFPCM(1,4-dioxane)-UB3LYP-GD3BJ/6-311+G(d,p)

Electronic Energy (EE) = -2946.8478 Hartree
Zero-point Energy Correction = 0.160974 Hartree
Thermal Correction to Energy = 0.173385 Hartree
Thermal Correction to Enthalpy = 0.174376 Hartree
Thermal Correction to Free Energy = 0.115517 Hartree
EE + Zero-point Energy = -2946.6868 Hartree
EE + Thermal Energy Correction = -2946.6744 Hartree
EE + Thermal Enthalpy Correction = -2946.6734 Hartree
EE + Thermal Free Energy Correction = -2946.7323 Hartree

B	-1.59207500	1.06124100	-0.06346000
O	-0.34206000	1.50041300	0.18459600
H	0.30769200	0.81110800	0.43251300
O	-2.59660500	1.89437800	-0.41815400
H	-2.31182500	2.81539600	-0.46739200
C	1.79077200	-0.49885000	0.83381700
C	1.93863100	-1.09440700	-0.53381400

C	2.89263400	0.48362400	1.11578400
H	1.17395200	-0.94160500	1.60827900
C	2.89950900	-0.12195200	-1.24723600
H	0.98674000	-1.23813000	-1.05426000
H	2.39531500	-2.09550000	-0.46017400
C	3.82133100	0.37261300	-0.11756500
H	3.40801000	0.27386700	2.05988700
H	2.50201600	1.50792800	1.21452900
H	3.44581800	-0.58840300	-2.06907800
H	2.33107400	0.71752100	-1.66080200
H	4.59597900	-0.37596300	0.07675200
H	4.32325100	1.31207000	-0.35593600
Br	-2.04987400	-0.84311400	0.07301500

IIIa

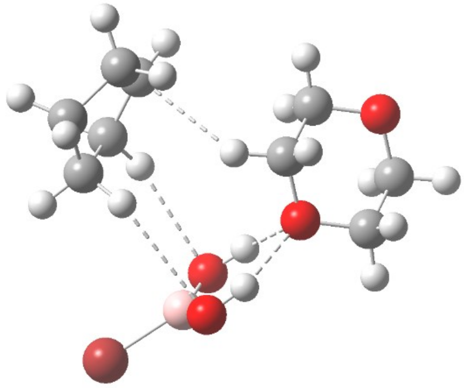

Method: IEFPCM(1,4-dioxane)-UB3LYP-GD3BJ/6-311+G(d,p)

Electronic Energy (EE) = -3254.6332 Hartree
Zero-point Energy Correction = 0.285237 Hartree
Thermal Correction to Energy = 0.30531 Hartree
Thermal Correction to Enthalpy = 0.306301 Hartree
Thermal Correction to Free Energy = 0.227625 Hartree
EE + Zero-point Energy = -3254.348 Hartree
EE + Thermal Energy Correction = -3254.3279 Hartree
EE + Thermal Enthalpy Correction = -3254.3269 Hartree
EE + Thermal Free Energy Correction = -3254.4056 Hartree

B	-1.47466100	-1.21495500	0.12790300
O	-1.04900200	-1.26600100	1.41279800
H	-0.07798600	-1.33697500	1.45498100
O	-0.68086500	-1.26716600	-0.96845200
H	0.25773200	-1.31316200	-0.70710100
C	0.97805900	2.93265000	-0.85056100
C	-0.35378300	2.30905600	-1.13314700
C	0.96463700	3.69873900	0.43753100
H	1.81521900	2.91687500	-1.53766300

C	-1.10307700	2.43282900	0.21160000
H	-0.29271100	1.27787000	-1.49548000
H	-0.89072900	2.86879800	-1.91710400
C	-0.52932800	3.71645200	0.83828800
H	1.39308800	4.70359700	0.34514600
H	1.56321600	3.19368100	1.21467500
H	-2.18763100	2.45811600	0.09523900
H	-0.86811500	1.57454100	0.84977400
H	-1.01457900	4.59100900	0.39320900
H	-0.68121600	3.76896700	1.91829800
Br	-3.39883700	-1.03871000	-0.17107900
C	2.57250400	-2.36145700	0.58565300
C	3.57980700	-2.17573300	-0.53630400
C	3.32463000	0.15553000	-0.41587600
C	2.31583400	0.00464900	0.70705800
H	4.34213400	-2.95482900	-0.49723900
H	3.07757900	-2.39659900	1.55703600
H	1.98570800	-3.27044100	0.44881900
H	3.90044500	1.07282900	-0.29028300
H	2.80317000	0.19473600	-1.38193800
H	2.81069000	0.04806700	1.68331200
H	1.55012500	0.77677400	0.64882000
H	3.07005800	-2.22584400	-1.50875500
O	1.63628800	-1.26442600	0.59005800
O	4.25366800	-0.92674200	-0.40827300

Secondary radical

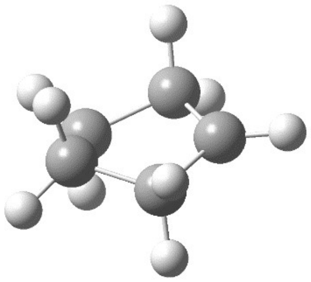

Method: IEFPCM(1,4-dioxane)-UB3LYP-GD3BJ/6-311+G(d,p)

Electronic Energy (EE) = -195.96783 Hartree
Zero-point Energy Correction = 0.125129 Hartree
Thermal Correction to Energy = 0.130833 Hartree
Thermal Correction to Enthalpy = 0.131824 Hartree
Thermal Correction to Free Energy = 0.094924 Hartree
EE + Zero-point Energy = -195.84271 Hartree
EE + Thermal Energy Correction = -195.837 Hartree
EE + Thermal Enthalpy Correction = -195.83601 Hartree
EE + Thermal Free Energy Correction = -195.87291 Hartree

C	-0.00004000	1.27658100	0.00006400
C	-1.23238200	0.43409500	0.12157700
C	1.23234600	0.43418200	-0.12158000
H	-0.00010700	2.35929600	-0.00018800
C	-0.73615900	-0.98805200	-0.22486000
H	-2.05194200	0.76912100	-0.52491300
H	-1.63112800	0.45913000	1.14945200
C	0.73623600	-0.98800200	0.22485300
H	2.05194100	0.76924000	0.52484900
H	1.63100100	0.45927000	-1.14949100
H	-1.32720300	-1.77487000	0.24879300
H	-0.79029500	-1.13964600	-1.30804700
H	0.79039300	-1.13960100	1.30803800
H	1.32733300	-1.77477100	-0.24881400

IV

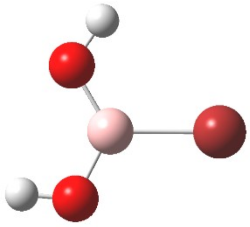

Method: IEFPCM(1,4-dioxane)-B3LYP-GD3BJ/6-311+G(d,p)

Electronic Energy (EE) = -2750.8704 Hartree
Zero-point Energy Correction = 0.034391 Hartree
Thermal Correction to Energy = 0.039308 Hartree
Thermal Correction to Enthalpy = 0.040299 Hartree
Thermal Correction to Free Energy = 0.00411 Hartree
EE + Zero-point Energy = -2750.836 Hartree
EE + Thermal Energy Correction = -2750.8311 Hartree
EE + Thermal Enthalpy Correction = -2750.8301 Hartree
EE + Thermal Free Energy Correction = -2750.8663 Hartree

B	-0.97070700	-0.00282900	0.00000100
O	-1.65860000	1.16491100	-0.00000400
H	-1.11193000	1.95680900	0.00003400
O	-1.59377900	-1.19910900	0.00001000
H	-2.55613800	-1.12258400	-0.00006000
Br	0.98687500	-0.01561400	-0.00000100

IV_a

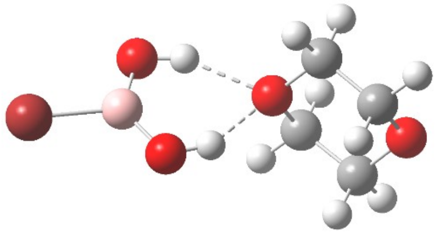

Method: IEFPCM(1,4-dioxane)-B3LYP-GD3BJ/6-311+G(d,p)

Electronic Energy (EE) = -3058.658 Hartree
Zero-point Energy Correction = 0.158914 Hartree
Thermal Correction to Energy = 0.171102 Hartree
Thermal Correction to Enthalpy = 0.172094 Hartree
Thermal Correction to Free Energy = 0.115851 Hartree
EE + Zero-point Energy = -3058.4991 Hartree
EE + Thermal Energy Correction = -3058.4869 Hartree
EE + Thermal Enthalpy Correction = -3058.4859 Hartree
EE + Thermal Free Energy Correction = -3058.5422 Hartree

B	-1.49714900	-0.00056000	-0.10176800
O	-1.05447900	-0.00400000	-1.38165400
H	-0.08044500	-0.00420500	-1.41291200
O	-0.71558700	0.00216500	1.00475500
H	0.22766400	0.00136300	0.75799900
Br	-3.43423600	0.00056300	0.17378400
C	2.43861200	1.19008600	-0.59836300
C	2.43952700	-1.19304700	-0.59284400
C	3.46970100	-1.17269000	0.52278500
C	3.46881000	1.17572800	0.51734700
H	1.75821100	-2.03852600	-0.49202400
H	2.92868500	1.22774500	-1.57726400
H	1.75662900	2.03549200	-0.50152800
H	2.96576200	-1.21489900	1.49845700
H	4.14312800	-2.02620300	0.43631200
H	2.96490600	1.22211800	1.49285600
H	4.14159100	2.02933400	0.42684700
H	2.92959400	-1.23489900	-1.57158200
O	4.27367600	0.00164600	0.44386100
O	1.62744100	-0.00164900	-0.53250100

H_2O

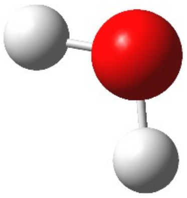

Method: IEFPCM(1,4-dioxane)-B3LYP-GD3BJ/6-311+G(d,p)

Electronic Energy (EE) = -76.462602 Hartree
Zero-point Energy Correction = 0.021244 Hartree
Thermal Correction to Energy = 0.024222 Hartree
Thermal Correction to Enthalpy = 0.025213 Hartree
Thermal Correction to Free Energy = 0.002526 Hartree
EE + Zero-point Energy = -76.441358 Hartree
EE + Thermal Energy Correction = -76.43838 Hartree
EE + Thermal Enthalpy Correction = -76.437389 Hartree
EE + Thermal Free Energy Correction = -76.460076 Hartree

O	0.00000000	0.00000000	0.11741500
H	0.00000000	-0.76310000	-0.46965900
H	0.00000000	0.76310000	-0.46965900

$TS_{IV\text{-}V}$

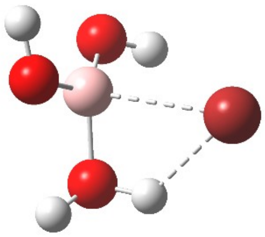

Method: IEFPCM(1,4-dioxane)-B3LYP-GD3BJ/6-311+G(d,p)

Electronic Energy (EE) = -2827.3246 Hartree
Zero-point Energy Correction = 0.058752 Hartree
Thermal Correction to Energy = 0.065533 Hartree
Thermal Correction to Enthalpy = 0.066524 Hartree
Thermal Correction to Free Energy = 0.026052 Hartree
EE + Zero-point Energy = -2827.2658 Hartree
EE + Thermal Energy Correction = -2827.259 Hartree
EE + Thermal Enthalpy Correction = -2827.258 Hartree
EE + Thermal Free Energy Correction = -2827.2985 Hartree

B	-1.26535800	0.11501500	0.00910900
O	-1.50670400	1.26646100	-0.65642600
H	-0.90887900	1.45281700	-1.38745300
O	-1.79977100	-0.21210000	1.20788700
H	-2.04201900	0.54758700	1.74781200
Br	1.31386000	0.04659300	0.08689300
O	-1.09065500	-1.18065100	-0.83137600
H	-1.45162700	-1.95368700	-0.36410300
H	-0.07874700	-1.24222000	-0.84372500

$TS_{IVa\text{-}Va}$

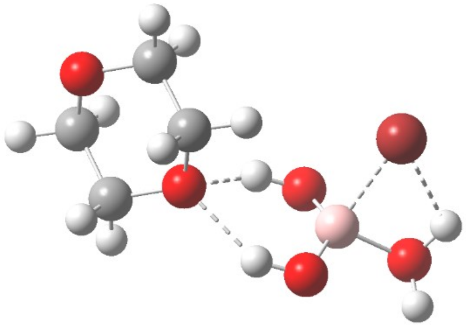

Method: IEFPCM(1,4-dioxane)-B3LYP-GD3BJ/6-311+G(d,p)

Electronic Energy (EE) = -3135.1143 Hartree
Zero-point Energy Correction = 0.18375 Hartree
Thermal Correction to Energy = 0.197926 Hartree
Thermal Correction to Enthalpy = 0.198917 Hartree
Thermal Correction to Free Energy = 0.138498 Hartree
EE + Zero-point Energy = -3134.9306 Hartree
EE + Thermal Energy Correction = -3134.9164 Hartree
EE + Thermal Enthalpy Correction = -3134.9154 Hartree
EE + Thermal Free Energy Correction = -3134.9758 Hartree

B	1.40422100	1.12764900	0.12375800
O	0.64382100	1.17577100	1.25304600
H	-0.27853200	0.97345900	1.02775400
O	1.03144100	1.51328500	-1.13541300
H	0.07671000	1.38078700	-1.25668600
Br	2.26624800	-1.12058400	-0.01331100
O	2.84638700	1.68832100	0.40552100
H	3.17597500	2.15495000	-0.38101600
H	3.38960100	0.87411700	0.53035200
C	-1.63939000	-0.80849600	-0.76361600
C	-2.83882000	1.21924100	-0.42098200
C	-3.72466200	0.50588200	0.58693300
C	-2.54060400	-1.49336800	0.24658300
H	-2.68066600	2.26211300	-0.14267200
H	-2.04097500	-0.91876200	-1.77716500
H	-0.62411600	-1.20475700	-0.72564200
H	-3.31286300	0.62967000	1.59871400
H	-4.73566300	0.91532200	0.56539800
H	-2.08562500	-1.44900800	1.24558100
H	-2.69220900	-2.53872500	-0.02450900
H	-3.28326500	1.17363900	-1.42195300
O	-3.82967900	-0.87976800	0.27480600
O	-1.54038200	0.60112100	-0.45853200

HBr

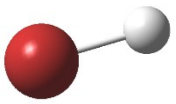

Method: IEFPCM(1,4-dioxane)-B3LYP-GD3BJ/6-311+G(d,p)

Electronic Energy (EE) = -2574.7551 Hartree
Zero-point Energy Correction = 0.005926 Hartree
Thermal Correction to Energy = 0.008404 Hartree
Thermal Correction to Enthalpy = 0.009395 Hartree
Thermal Correction to Free Energy = -0.014432 Hartree
EE + Zero-point Energy = -2574.7491 Hartree
EE + Thermal Energy Correction = -2574.7467 Hartree
EE + Thermal Enthalpy Correction = -2574.7457 Hartree
EE + Thermal Free Energy Correction = -2574.7695 Hartree

Br	0.00000000	0.00000000	0.03967000
H	0.00000000	0.00000000	-1.38844500

Boronic acid (V)

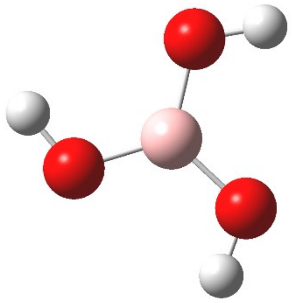

Method: IEFPCM(1,4-dioxane)-B3LYP-GD3BJ/6-311+G(d,p)

Electronic Energy (EE) = -252.5948 Hartree
Zero-point Energy Correction = 0.048649 Hartree
Thermal Correction to Energy = 0.053424 Hartree
Thermal Correction to Enthalpy = 0.054415 Hartree
Thermal Correction to Free Energy = 0.020616 Hartree
EE + Zero-point Energy = -252.54615 Hartree
EE + Thermal Energy Correction = -252.54138 Hartree
EE + Thermal Enthalpy Correction = -252.54039 Hartree
EE + Thermal Free Energy Correction = -252.57419 Hartree

B	-0.00006700	-0.00002400	0.00002000
O	0.30966100	1.33429300	0.00001300

H	1.25788600	1.49850100	-0.00017200
O	-1.31046300	-0.39902200	-0.00003100
H	-1.92698000	0.33990000	0.00013100
O	1.00080700	-0.93522100	0.00001900
H	0.66939700	-1.83869100	-0.00006100

Va

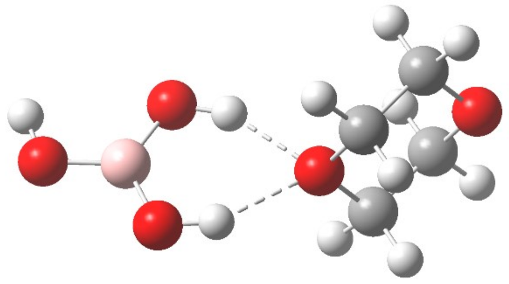

Method: IEFPCM(1,4-dioxane)-B3LYP-GD3BJ/6-311+G(d,p)

Electronic Energy (EE) = -560.38027 Hartree
Zero-point Energy Correction = 0.173285 Hartree
Thermal Correction to Energy = 0.185316 Hartree
Thermal Correction to Enthalpy = 0.186308 Hartree
Thermal Correction to Free Energy = 0.131184 Hartree
EE + Zero-point Energy = -560.20699 Hartree
EE + Thermal Energy Correction = -560.19496 Hartree
EE + Thermal Enthalpy Correction = -560.19397 Hartree
EE + Thermal Free Energy Correction = -560.24909 Hartree

B	-2.72011500	0.00001300	-0.09877500
O	-2.31095400	-0.00028000	1.20832600
H	-1.34394200	-0.00028400	1.27366700
O	-1.84911900	0.00024300	-1.16652400
H	-0.92584900	0.00015900	-0.86780000
C	1.24651400	-1.18965900	0.60300600
C	1.24646600	1.18942800	0.60343100
C	2.33952800	1.17450600	-0.45104000
C	2.33958600	-1.17431800	-0.45145000
H	0.57150700	2.03501200	0.46623700
H	1.68080000	-1.23140300	1.60822800
H	0.57159000	-2.03522000	0.46550400
H	1.89193300	1.21934000	-1.45374900
H	3.00682000	2.02782100	-0.32352900
H	1.89200900	-1.21883300	-1.45418100
H	3.00691900	-2.02764400	-0.32422200
H	1.68075700	1.23082800	1.60866400
O	3.13908000	0.00009300	-0.33113800
O	0.44143700	-0.00011200	0.49347300
O	-4.07009700	0.00007600	-0.33561100

H	-4.26730400	0.00026800	-1.27682600

Tentative TS for the reaction of residual water with a bromine radical

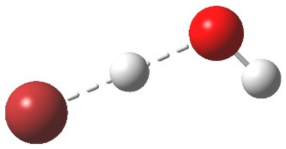

Method: IEFPCM(1,4-dioxane)-UB3LYP-GD3BJ/6-311+G(d,p)

Electronic Energy (EE) = -2650.5218 Hartree
Zero-point Energy Correction = 0.01555 Hartree
Thermal Correction to Energy = 0.019134 Hartree
Thermal Correction to Enthalpy = 0.020125 Hartree
Thermal Correction to Free Energy = -0.01186 Hartree
EE + Zero-point Energy = -2650.5063 Hartree
EE + Thermal Energy Correction = -2650.5027 Hartree
EE + Thermal Enthalpy Correction = -2650.5017 Hartree
EE + Thermal Free Energy Correction = -2650.5337 Hartree

O	0.02055800	2.34073900	0.00000000
H	-0.89918400	2.67102800	0.00000000
H	0.01518300	0.89862500	0.00000000
Br	0.02055800	-0.63701600	0.00000000

Pinacol cyclopentyl boronate

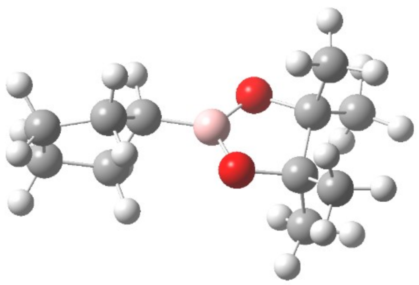

Method: IEFPCM(1,4-dioxane)-B3LYP-GD3BJ/6-311+G(d,p)

Electronic Energy (EE) = -607.47124 Hartree
Zero-point Energy Correction = 0.311136 Hartree
Thermal Correction to Energy = 0.327687 Hartree
Thermal Correction to Enthalpy = 0.328678 Hartree
Thermal Correction to Free Energy = 0.264618 Hartree
EE + Zero-point Energy = -607.1601 Hartree
EE + Thermal Energy Correction = -607.14355 Hartree
EE + Thermal Enthalpy Correction = -607.14256 Hartree
EE + Thermal Free Energy Correction = -607.20662 Hartree

B	-0.25078100	0.31045900	-0.11322000
O	0.30819200	-0.65401700	0.69212800
O	0.69460500	1.02382900	-0.80934900
C	-1.79171500	0.52989100	-0.24034300
C	-2.50573700	-0.60398600	-1.01686600
C	-2.56527600	0.52603800	1.09602800
H	-1.98585300	1.47613900	-0.76069900
C	-4.00260400	-0.42440500	-0.67897000
H	-2.14976500	-1.57156500	-0.64742100
H	-2.30862800	-0.57150300	-2.09115600
C	-4.04171600	0.29566100	0.69900000
H	-2.42295600	1.44506600	1.67013200
H	-2.20653300	-0.30233000	1.71479500
H	-4.48692800	0.19135200	-1.44150900
H	-4.52954800	-1.38127900	-0.66128900
H	-4.56361300	1.25217800	0.61115100
H	-4.57420400	-0.28768800	1.45381400
C	1.72069900	-0.75979200	0.34398900
C	2.00833600	0.64619200	-0.29590200
C	2.51524300	-1.06542200	1.60508400
H	2.24063500	-2.05353400	1.97976900
H	3.58722400	-1.06866900	1.39091300
H	2.31739100	-0.33619800	2.38972600
C	1.84165600	-1.91290800	-0.65396200
H	2.88297600	-2.09695700	-0.92614400
H	1.43948400	-2.81825100	-0.19556600
H	1.27368000	-1.71060600	-1.56414400
C	2.39933900	1.70841100	0.73286300
H	3.40183100	1.53021000	1.12752200
H	2.38758100	2.68740000	0.25024400
H	1.69496100	1.73047700	1.56665900
C	3.00061200	0.63507800	-1.44940700
H	3.12751000	1.64955800	-1.83265100
H	3.97606500	0.27440000	-1.11235900
H	2.65719500	0.00246400	-2.26700800

TS employing pinacol pentenyl boronate

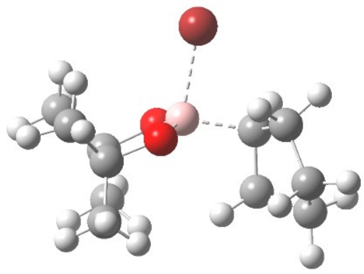

Method: IEFPCM(1,4-dioxane)-UB3LYP-GD3BJ/6-311+G(d,p)

Electronic Energy (EE) = -3181.5124 Hartree
Zero-point Energy Correction = 0.304926 Hartree
Thermal Correction to Energy = 0.32048 Hartree
Thermal Correction to Enthalpy = 0.321471 Hartree
Thermal Correction to Free Energy = 0.259203 Hartree
EE + Zero-point Energy = -3181.2074 Hartree
EE + Thermal Energy Correction = -3181.1919 Hartree
EE + Thermal Enthalpy Correction = -3181.1909 Hartree
EE + Thermal Free Energy Correction = -3181.2532 Hartree

B	0.01453900	0.17693400	-0.18024500
O	-0.26452300	-0.28622800	1.08439300
O	-0.82276000	-0.00132000	-1.25725200
C	1.71848700	-0.10990000	-0.62274100
C	2.71720900	0.04727800	0.53391100
C	1.68271100	-1.60753600	-1.00976700
H	1.93609600	0.52153800	-1.47800100
C	3.30972900	-1.36212100	0.75343100
H	2.24587700	0.43377400	1.43658300
H	3.48867800	0.76716400	0.24687300
C	3.08545600	-2.08148000	-0.58556300
H	1.45546300	-1.73757700	-2.06782900
H	0.93667100	-2.15901300	-0.43305000
H	4.35953100	-1.33228400	1.05184500
H	2.75662500	-1.87913900	1.54320400
H	3.82840400	-1.75569000	-1.32092500
H	3.14190300	-3.17053200	-0.50966000
Br	0.44015900	2.42555100	-0.04158700
C	-2.05454400	-0.60286700	-0.85012900
C	-3.22894400	0.34875700	-1.14474500
H	-3.29120100	0.52283100	-2.19865300
H	-4.14096200	-0.09340100	-0.80178800
H	-3.07064700	1.27803200	-0.63849500
C	-2.15675900	-1.84133300	-1.75971900
H	-3.07234700	-2.35714500	-1.55838000
H	-2.13854200	-1.53376700	-2.78440000
H	-1.33040800	-2.49358100	-1.56836400

C	-1.58793300	-0.82280100	1.15905600
C	-1.55030600	-2.27851500	1.66013600
H	-1.28301700	-2.29232000	2.69612200
H	-2.51507400	-2.72393300	1.53473800
H	-0.82668400	-2.83072700	1.09770200
C	-2.26660700	0.06690400	2.21708600
H	-3.29485700	-0.21333700	2.31231200
H	-1.77387100	-0.05934300	3.15845400
H	-2.20263900	1.09156200	1.91561800

References

1. Primer, D. N., Karakaya, I., Tellis, J. C. & Molander, G. A. Single-electron transmetalation: An enabling technology for secondary alkylboron cross-coupling. *J Am Chem Soc* **137**, 2195–2198 (2015).

2. Zuo, Z. & Macmillan, D. W. C. Decarboxylative arylation of α-Amino acids via photoredox catalysis: A one-step conversion of biomass to drug pharmacophore. *J Am Chem Soc* **136**, 5257–5260 (2014).

3. Luridiana, A. *et al.* The Merger of Benzophenone HAT Photocatalysis and Silyl Radical-Induced XAT Enables Both Nickel-Catalyzed Cross-Electrophile Coupling and 1,2-Dicarbofunctionalization of Olefins. *ACS Catal* **12**, 11216–11225 (2022).

4. Campbell, P. S., Jamieson, C., Simpson, I. & Watson, A. J. B. Practical synthesis of pharmaceutically relevant molecules enriched in sp3 character. *Chemical Communications* **54**, 46–49 (2017).

5. Yadav, C., Maka, V. K., Payra, S. & Moorthy, J. N. Multifunctional porous organic polymers (POPs): Inverse adsorption of hydrogen over nitrogen, stabilization of Pd(0) nanoparticles, and catalytic cross-coupling reactions and reductions. *J Catal* **384**, 61–71 (2020).

6. Huang, L., Kancherla, R. & Rueping, M. Nickel Catalyzed Regiodivergent Cross-Coupling Alkylation of Aryl Halides with Redox-Active Imines. *ACS Catal* **12**, 11563–11572 (2022).

7. Lv, X. Y., Abrams, R. & Martin, R. Dihydroquinazolinones as adaptative C(sp3) handles in arylations and alkylations via dual catalytic C–C bond-functionalization. *Nature Communications 2022 13:1* **13**, 1–8 (2022).

8. Meng, Q. Y., Schirmer, T. E., Katou, K. & König, B. Controllable Isomerization of Alkenes by Dual Visible-Light-Cobalt Catalysis. *Angewandte Chemie - International Edition* **58**, 5723–5728 (2019).

9. Cong, F., Lv, X. Y., Day, C. S. & Martin, R. Dual Catalytic Strategy for Forging sp2-sp3and sp3-sp3Architectures via β -Scission of Aliphatic Alcohol Derivatives. *J Am Chem Soc* **142**, 20594–20599 (2020).

10. Abdiaj, I. & Alcázar, J. Improving the throughput of batch photochemical reactions using flow: Dual photoredox and nickel catalysis in flow for C(sp2)C(sp3) cross-coupling. *Bioorg Med Chem* **25**, 6190–6196 (2017).

11. Gong, Y. *et al.* Nickel-Catalyzed Thermal Redox Functionalization of C(sp3)−H Bonds with Carbon Electrophiles. *Angewandte Chemie International Edition* **61**, e202201662 (2022).

12. Qin, J., Zhou, Z., Cui, T., Hemming, M. & Meggers, E. Enantioselective intramolecular C–H amination of aliphatic azides by dual ruthenium and phosphine catalysis. *Chem Sci* **10**, 3202–3207 (2019).

13. Luo, J. & Zhang, J. Donor-Acceptor Fluorophores for Visible-Light-Promoted Organic Synthesis: Photoredox/Ni Dual Catalytic C(sp3)-C(sp2) Cross-Coupling. *ACS Catal* **6**, 873–877 (2016).

14. Chen, Y., Wang, X., He, X., An, Q. & Zuo, Z. Photocatalytic Dehydroxymethylative Arylation by Synergistic Cerium and Nickel Catalysis. *J Am Chem Soc* **143**, 4896–4902 (2021).

15. Shaw, M. H., Shurtleff, V. W., Terrett, J. A., Cuthbertson, J. D. & MacMillan, D. W. C. Native functionality in triple catalytic cross-coupling: sp3 C-H bonds as latent nucleophiles. *Science (1979)* **352**, 1304–1308 (2016).

16. Llaveria, J., Leonori, D. & Aggarwal, V. K. Stereospecific Coupling of Boronic Esters with N-Heteroaromatic Compounds. *J Am Chem Soc* **137**, 10958–10961 (2015).

17. Yin, H., Zhao, C., You, H., Lin, K. & Gong, H. Mild ketone formation via Ni-catalyzed reductive coupling of unactivated alkyl halides with acid anhydrides. *Chemical Communications* **48**, 7034–7036 (2012).

18. Xi, X. *et al.* From Esters to Ketones via a Photoredox-Assisted Reductive Acyl Cross-Coupling Strategy. *Angewandte Chemie International Edition* **61**, e202114731 (2022).

19. Allwood, D. M., Blakemore, D. C. & Ley, S. V. Preparation of unsymmetrical ketones from tosylhydrazones and aromatic aldehydes via formyl C-H bond insertion. *Org Lett* **16**, 3064–3067 (2014).

20. Liu, W. C. *et al.* Energy-Transfer-Enabled Radical Acylation Using Free Alkyl Boronic Acids through Photo and NHC Dual Catalysis. *ACS Catal* **14**, 3181–3190 (2024).

21. Gaussian 16, Revision C.01, Frisch, M. J.; Trucks, G. W.; Schlegel, H. B.; Scuseria, G. E.; Robb, M. A.; Cheeseman, J. R.; Scalmani, G.; Barone, V.; Petersson, G. A.; Nakatsuji, H.; Li, X.; Caricato, M.; Marenich, A. V.; Bloino, J.; Janesko, B. G.; Gomperts, R.; Mennucci, B.; Hratchian, H. P.; Ortiz, J. V.; Izmaylov, A. F.; Sonnenberg, J. L.; Williams-Young, D.; Ding, F.; Lipparini, F.; Egidi, F.; Goings, J.; Peng, B.; Petrone, A.; Henderson, T.; Ranasinghe, D.; Zakrzewski, V. G.; Gao, J.; Rega, N.; Zheng, G.; Liang, W.; Hada, M.; Ehara, M.; Toyota, K.; Fukuda, R.; Hasegawa, J.; Ishida, M.; Nakajima, T.; Honda, Y.; Kitao, O.; Nakai, H.; Vreven, T.; Throssell, K.; Montgomery, Jr., J. A.; Peralta, J. E.; Ogliaro, F.; Bearpark, M. J.; Heyd, J. J.; Brothers, E. N.; Kudin, K. N.; Staroverov, V. N.; Keith, T. A.; Kobayashi, R.; Normand, J.; Raghavachari, K.; Rendell, A. P.; Burant, J. C.; Iyengar, S. S.; Tomasi, J.; Cossi, M.; Millam, J. M.; Klene, M.; Adamo, C.; Cammi, R.; Ochterski, J. W.; Martin, R. L.; Morokuma, K.; Farkas, O.; Foresman, J. B.; Fox, D. J. Gaussian, Inc., Wallingford CT, **2019**.

22. Becke, A. D. *J. Chem. Phys.*, **1993**, *98*, 5648-5652.

23. Ditchfield, R.; Hehre, W. J.; Pople, J. A. *J. Chem. Phys.*, **1971**, *54*, 724-728; Hehre, W. J. Ditchfield, R.; Pople, J. A. *J. Chem. Phys.*, **1972**, *56*, 2257-2261; Hariharan, P. C.; Pople, J.

A. *Theor. Chem. Acc.*, **1973**, *28*, 213-222.

24. Raghavachari, K.; Binkley, J. S.; Seeger, R.; Pople, J. A. *J. Chem. Phys.*, **1980**, *72*, 650-654, Clark, T.; Chandrasekhar, J.; Spitznagel, G. W.; Schleyer, P. V. R. *J. Comp. Chem.*, **1983**, *4*, 294-301; Frisch, M. J.; Pople, J. A.; Binkley, J. S. *J. Chem. Phys.*, **1984**, *80*, 3265-3269.

25. S. Grimme, S.; Ehrlich, S.; Goerigk, L. *J. Comp. Chem.*, **2011**, ***32,*** 1456-1465.
